# Tau isoform mapping across tissues and biofluids reveals a signature distinguishing Alzheimer’s disease from amyotrophic lateral sclerosis

**DOI:** 10.1038/s41467-026-78390-3

**Published:** 2026-10-22

**Authors:** Samir Abu-Rumeileh, Henning Großkopf, Arthur Guthknecht, Alexander Mensch, Dirk Osterloh, Antonia Ratti, Julian Conrad, Caroline Stapf, Julia Krippner, Patrick Oeckl, Gisela Stoltenburg-Didinger, Alessio Maranzano, Petra Steinacker, Christopher M. Weise, Lorenzo Barba, Lucio D’Anna, Hans-Wolfgang Klafki, Jens Wiltfang, Vincenzo Silani, Lutz Froelich, Nicola Ticozzi, Lucrezia Hausner, Dietmar Rudolf Thal, Jochen Hans Weishaupt, Ingolf Lachmann, Federico Verde, Markus Otto

**Affiliations:** 1 Department of Neurology, Martin-Luther-University Halle-Wittenberg, Halle (Saale), Germany; 2 BG University Hospital Bergmannsheil, Heimer Institute for Muscle Research, Bochum, Germany; 3 Roboscreen GmbH, Leipzig, Germany; 4 Department of Neurology and Laboratory of Neuroscience, IRCCS Istituto Auxologico Italiano, Milan, Italy; 5 Department of Medical Biotechnology and Translational Medicine, Università degli Studi di Milano, Milan, Italy; 6 Division for Neurodegenerative Diseases, Department of Neurology, Mannheim Center for Translational Medicine, University Medicine Mannheim, Heidelberg University, Mannheim, Germany; 7 Department of Neurology, Ulm University Hospital, Ulm, Germany; 8 German Center for Neurodegenerative Diseases (DZNE e.V.), Ulm, Germany; 9 Institute of Cell Biology and Neurobiology, Charité, Universitätsmedizin Berlin, Berlin, Germany; 10 Department of Brain Sciences, Imperial College London, London, UK; 11 Department of Stroke and Neuroscience, Charing Cross Hospital, Imperial College, Healthcare NHS Trust, London, UK; 12 Department of Psychiatry and Psychotherapy, University Medical Center Goettingen (UMG), Georg-August University, Goettingen, Germany; 13 Department of Pathophysiology and Transplantation, “Dino Ferrari” Center, Università degli Studi di Milano, Milan, Italy; 14 Department of Geriatric Psychiatry, Central Institute of Mental Health, Medical Faculty Mannheim, Heidelberg University, Mannheim, Germany; 15 Laboratory for Neuropathology, Department of Imaging and Pathology and Leuven Brain Institute (LBI), Leuven, Belgium; 16 Department of Pathology, University Hospitals Leuven, Leuven, Belgium

**Keywords:** Alzheimer's disease, Amyotrophic lateral sclerosis, Diagnostic markers, Amyotrophic lateral sclerosis, Neurodegeneration

## Abstract

Blood phosphorylated tau 217 (p-tau217) accurately detects Alzheimer’s disease (AD) pathology but is also elevated in amyotrophic lateral sclerosis (ALS), likely reflecting peripheral (big) tau released from muscle. Assays selectively measuring brain-derived (BD-) tau and BD-p-tau217 are therefore needed. We analysed serum BD-tau and p-tau217 in ALS (*n* = 139), AD (*n* = 101) and controls (*n* = 75) using direct immunoassays and after BD-Tau immunoprecipitation (BD-Tau-IP), and tau species in brain (*n* = 16), spinal cord (*n* = 11) and muscle (*n* = 10) by targeted mass spectrometry. With the direct immunoassays, BD-tau and p-tau217 were increased in AD and ALS versus controls. After BD-Tau-IP, BD-tau and BD-p-tau217 were elevated in AD only, improving diagnostic accuracy. Mass spectrometry revealed a central-to-peripheral shift from BD-tau to big tau predominance, with exclusively big p-tau217 detected in muscle. Selective detection of BD-tau species improves the specificity of blood AD biomarkers by reducing peripheral tau confounding.

## Abstract (web summary)

Blood p-tau217 is a leading Alzheimer’s test but is also raised in ALS. Here, the authors show that muscle makes its own form of tau which leaks into the blood, and that detecting brain-derived p-tau217 improves the test’s accuracy.

## Introduction

Following the recent approval of anti-amyloid treatments as disease-modifying therapies for Alzheimer’s disease (AD), there is an urgent need for cost-effective and widely applicable biomarkers that can identify suitable candidates for these therapies and enhance patient screening, recruitment, and monitoring in clinical trials^1,2^. Phosphorylated tau 217 (p-tau217) and brain-derived tau (BD-tau) have emerged as presumably central nervous system (CNS)-specific and highly accurate cerebrospinal fluid (CSF) and blood biomarkers for the detection of AD pathology, with p-tau217 showing the highest diagnostic performance across different analytical assays^3–5^. Similarly, CSF and blood BD-tau levels have been shown to track amyloid-β-associated neurodegeneration in AD and to discriminate autopsy-confirmed AD from other neurodegenerative conditions^6,7^.

Nevertheless, recent data have challenged earlier assumptions regarding blood p-tau217 being exclusively brain-derived. Both our group and others have reported increased p-tau217 levels in the blood, but not in the CSF, of patients with amyotrophic lateral sclerosis (ALS)^2,8,9^. Furthermore, we identified p-tau217 species in muscle biopsy samples from both ALS patients and control subjects, with elevated serum concentrations likely reflecting protein release from denervated muscle fibres^8^. These findings reflect the distinct biology of tau: in the CNS, tau is predominantly expressed as low-molecular-weight (LMW) isoforms (37–46 kDa, BD-tau or small tau), whereas peripheral tissues mainly express high-molecular-weight (HMW) isoforms (~110 kDa, big tau)^2,10–12^. This difference arises from alternative splicing, as big tau includes additional exons, most notably the 250-amino-acid exon 4a, while BD-tau isoforms lack exon 4a^11,12^. A recent study demonstrated that big tau variants are physiologically distributed throughout the nervous system, with expression increasing from the CNS to the peripheral nervous system (PNS)^12^. Accordingly, BD-tau species constitute approximately 99% of total tau in CSF, whereas a substantial fraction of circulating tau in blood consists of big tau^12^.

Most commercially available p-tau217 immunoassays (e.g. ALZpath p-Tau 217) target an epitope shared by both BD- and big tau variants, without discriminating between brain-derived and peripheral species^2,8,12^, such as phosphorylated tau isoforms from muscle fibres in ALS. To date, only one assay, the p-tau217 assay developed by Lilly Research laboratories^13^, has demonstrated reduced cross-reactivity with big tau, resulting in fewer false positives while still detecting moderately increased p-tau217 levels in ALS compared with controls^2^. In contrast, the original prototype BD-tau assay, as well as the subsequently released commercial Simoa BD-tau assay (BD-tau Advantage PLUS) have been described as targeting a CNS-enriched tau epitope, which has been proposed to preferentially reflect BD-tau species in CSF and blood^6,7,14^. However, to date only one study, using the prototype assay, has examined the distribution of blood BD-tau in ALS compared with AD, reporting a small non-significant increase in plasma BD-tau in the former compared with controls, while observing no difference between AD and ALS^2^.

Taken together, these observations highlight the necessity to clarify this issue and to search for highly selective assays targeting BD-tau and BD-p-tau217 to limit the influence of peripheral big tau and to optimize diagnostic accuracy. In two previous AD biomarker studies, we adopted a two-step immunoassay approach, consisting of magnetic bead–based tau immunoprecipitation (Tau-IP) followed by p-tau quantification using conventional immunoassays^15,16^. The pre-analytical Tau-IP employed both a pan-tau antibody and a phosphorylation-insensitive BD-tau antibody targeting an epitope at the exon 4–exon 5 junction, and was intended to reduce potential matrix interferences. Using this approach, an improved diagnostic performance was observed for blood p-tau181, but not for p-tau217, in predicting CSF amyloid-β positivity^15,16^. Importantly, in these studies the co-enrichment of big tau species could not be excluded due to the inclusion of a pan-tau antibody. Consequently, the potential of a BD-tau–specific immunoprecipitation (BD-Tau-IP) using exclusively anti–BD-tau–functionalized magnetic beads for selective BD-tau enrichment has not yet been investigated.

In this multicentre study, we investigated serum and CSF BD-tau as well as serum p-tau217 levels in patients with ALS compared with those with AD. We additionally included disease controls and healthy controls. In a large cohort of well-characterised ALS cases, we investigated associations between BD-tau concentrations (in serum and CSF) and clinical or demographic variables.

Furthermore, we developed a two-step approach involving a selective BD-Tau-IP from serum, followed by quantification of BD-tau and p-tau217 using commercially available Simoa immunoassays. Briefly, the BD-Tau-IP represents a pre-analytical enrichment step that selectively captures BD-tau isoforms from serum using antibody-functionalized magnetic beads, thereby concentrating BD-tau species while removing interfering peripheral tau isoforms. The captured BD-tau species are subsequently eluted from the beads by heat denaturation, and the resulting eluates are used for quantification of BD-tau and p-tau217 on the Simoa platform. We then compared the diagnostic performance of this two-step approach with that of serum BD-tau and p-tau217 measured directly using the commercial immunoassays (one-step approach).

Finally, we analysed the presence of specific BD-tau and peripheral (big) tau peptides in brain tissue from AD, ALS, and disease controls, as well as in spinal cord and muscle samples from ALS patients and controls, using pre-analytical pan-tau or p-tau217 immunoprecipitation (pan-tau-IP and p-tau217-IP) followed by targeted mass spectrometry.

## Results

### One-step approach: serum and CSF tau biomarkers in the diagnostic groups using direct Simoa immunoassays

**Fig. 1:**
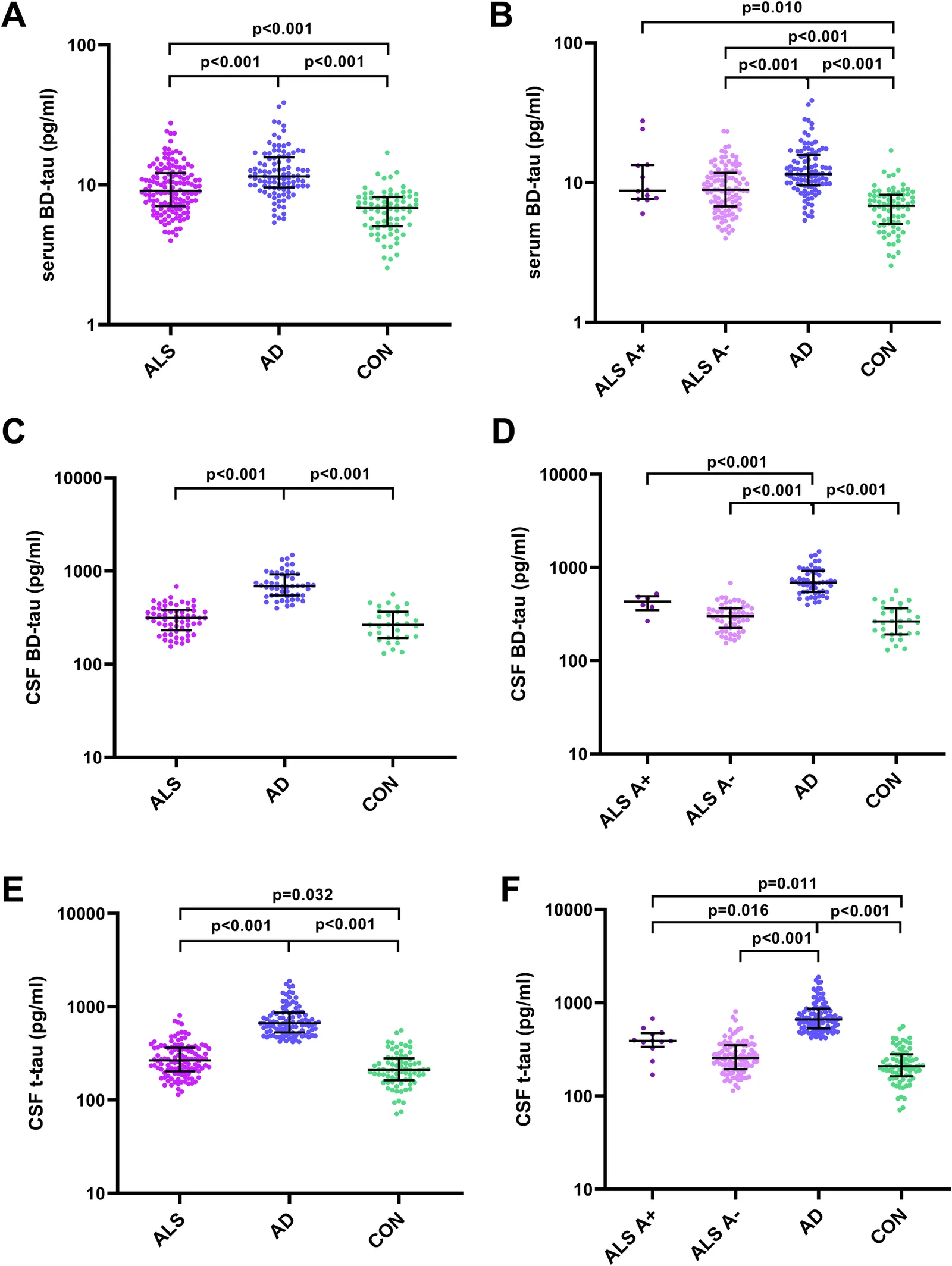
Distribution of serum BD-tau, CSF BD-tau and CSF t-tau in the diagnostic groups (one-step approach). **A** Distribution of serum BD-tau in the total cohort of ALS (*n *= 139), AD cases (*n *= 101) and disease controls (CON, *n *= 75). **B** Distribution of serum BD-tau in the same diagnostic groups after stratification of ALS cases according to the presence (ALS A+) or absence (ALS A−) of AD co-pathology. **C** Distribution of CSF BD-tau in the total cohort of ALS (*n *= 61), AD cases (*n *= 51) and disease controls (CON, *n *= 31). **D** Distribution of CSF BD-tau in the same diagnostic groups after stratification of ALS cases according to the presence (ALS A+) or absence (ALS A−) of AD co-pathology. **E** Distribution of CSF t-tau in the total cohort of ALS (*n *= 116), AD cases (*n *= 101) and disease controls (CON, *n *= 75). **F** Distribution of CSF t-tau in the same diagnostic groups after stratification of ALS cases according to the presence (ALS A+) or absence (ALS A−) of AD co-pathology. Y-axis shown on a logarithmic scale. Dots represent single data points. Horizontal lines represent the median values, the lower and upper lines correspond to the first and third quartiles, and the vertical line is the interquartile range. Biomarker differences between groups were assessed by Mann–Whitney or Kruskal–Wallis followed by Dunn’s post hoc test (adjustment for multiple comparisons). Two-sided *p*-values are reported. AD Alzheimer’s disease, ALS amyotrophic lateral sclerosis, ALS A+ amyotrophic lateral sclerosis with AD co-pathology, ALS A- amyotrophic lateral sclerosis without AD co-pathology, BD-tau brain-derived tau protein, CON disease controls, t-tau total tau protein. Source data are provided as a Source Data file.

Demographic characteristics and serum (BD-tau and p-tau217) and CSF biomarker (BD-tau and t-tau) distributions in the multicentre cohort measured by the one-step approach (direct Simoa immunoassays) are displayed in Table 1, Fig. 1, and Supplementary Table 1. Serum and CSF tau biomarkers across diagnostic groups in the individual cohorts are reported in Supplementary Tables 2–5.

**Table 1:** Demographic characteristics and biomarker distribution in the whole sample

Whole cohort	ALS	AD	Disease controls	*p*-value
*N* with serum samples	139^a^	101	75	
Age (years) mean ± SD	62.23 ± 11.75	74.52 ± 7.46	60.89 ± 13.80	<0.001
Sex: Female (%)	31.7	57.4	54.7	<0.001
Serum BD-tau (pg/mL) Median (IQR)	9.07 (7.04–12.15)	11.49 (9.58–15.76)	6.90 (5.10–8.20)	<0.001
Serum p-tau217 (pg/mL) Median (IQR)	0.370 (0.190–0.550)	0.750 (0.420–0.930)	0.150 (0.110–0.200)	<0.001
CSF BD-tau (pg/mL) Median (IQR)	314.13 (231.51–382.92)	687.90 (546.19–922.04)	263.59 (191.02–364.62)	<0.001
CSF t-tau (pg/mL) Median (IQR)	267 (202–363)	666 (530–866)	209 (163–281)	<0.001

Age, sex and biomarkers (serum BD-tau, serum p-tau217, CSF BD-tau and CSF t-tau) in the three diagnostic groups from all cohorts are displayed as mean ± standard deviation (SD), median and interquartile range (IQR) or as percentage. Depending on the type and distribution of the data, two-sided *p*-values of Kruskal–Wallis, ANOVA or Chi-squared test are reported. Dunn’s post-hoc tests (adjustments for multiple comparisons) to compare biomarker levels: serum BD-tau: AD vs. controls *p* < 0.001; AD vs. ALS *p* < 0.001; ALS vs. disease controls *p* < 0.001; serum p-tau217: AD vs. controls *p* < 0.001; AD vs. ALS *p* < 0.001; ALS vs. disease controls *p* < 0.001; CSF BD-tau: AD vs. controls *p* < 0.001; AD vs. ALS *p* < 0.001; ALS vs. disease controls *p* = 0.794; CSF t-tau: AD vs. ALS *p* < 0.001, ALS vs. controls *p* = 0.032, AD vs. controls *p* < 0.001.

*AD* Alzheimer’s disease, *ALS* amyotrophic lateral sclerosis, *BD-tau* brain-derived tau protein, *CSF* cerebrospinal fluid, *IQR* interquartile range, *N* number of cases, *p-tau217* phosphorylated tau protein 217, *SD* standard deviation, *t-tau* total tau protein.

^a^116 ALS cases with both serum and CSF samples.

In the whole sample, AD cases (*n *= 101) were older than ALS cases (*n *= 139, *p* < 0.001) and disease controls (*n *= 75, *p* < 0.001), whereas ALS participants were slightly older than disease controls (*p* = 0.042). Healthy controls (*n *= 22) were significantly younger than all other groups (all *p* < 0.001). Sex distribution differed significantly across groups (*p* < 0.001) (Table 1). We found significant positive associations between serum and CSF BD-tau, CSF t-tau, serum p-tau217 and age in the whole cohort and in the single diagnostic groups (Supplementary Results). Serum p-tau217 levels were significantly higher in males than in females (*p* = 0.005), whereas no significant sex-related differences were observed for the other investigated serum and CSF biomarkers (Supplementary Table 6). Taking these findings into account, comparisons of serum and CSF BD-tau and CSF t-tau between diagnostic groups were age-adjusted, whereas p-tau217 comparisons were age- and sex-adjusted. Furthermore, as CSF t-tau was measured in different centres, the corresponding comparison analyses were also centre-adjusted.

In the whole sample, patients with AD (*n *= 101) and ALS (*n *= 139) showed higher serum BD-tau levels (one-step approach) compared with disease controls (*n *= 75; both *p* < 0.001), with higher levels in AD than in ALS (*p* < 0.001) (Table 1, Fig. 1A). These results were replicated after age-adjustment (AD, *n *= 101 vs. disease controls, *n *= 75: β = 0.494, *p* < 0.001; ALS, *n *= 139 vs. disease controls, *n *= 75: β = 0.373, *p* < 0.001; AD, *n *= 101 vs. ALS, *n *= 139: β = 0.154, *p* = 0.027), in the largest individual cohorts (Supplementary Tables 2–3) and after including healthy controls in the analysis (Supplementary Results). Both ALS without AD co-pathology (ALS A−, *n *= 104) and ALS patients with AD co-pathology (ALS A+, *n *= 12) showed higher serum BD-tau concentrations than disease controls (*n *= 75; *p* < 0.001 and *p * = 0.010, respectively) (Fig. 1B).

In the whole cohort, patients with AD (*n *= 101) and ALS (*n *= 139) yielded higher serum p-tau217 values compared to disease controls (*n *= 75, both *p* < 0.001), consistent with our previous study^8^. Moreover, serum p-tau217 concentrations were higher in AD compared to ALS (*p* < 0.001)^8^. These group-level findings were confirmed after age- and sex-adjustment (AD, *n *= 101 vs. disease controls, *n *= 75: β = 0.712, *p* < 0.001; ALS, *n *= 139 vs. disease controls, *n *= 75: β = 0.435, *p* < 0.001; AD, *n *= 101 vs. ALS, *n *= 139: β = 0.405, *p* < 0.001).

AD participants (*n *= 51) had higher CSF BD-tau concentrations compared with ALS cases (*n *= 61) and disease controls (*n *= 31; both *p* < 0.001), with no difference between the latter two groups (Table 1, Fig. 1C). These results were largely confirmed in the Halle and Milan cohorts (Supplementary Tables 2–3) and after age-adjustment (AD, *n *= 51 vs. disease controls, *n *= 31: β = 0.705, *p* < 0.001; AD, *n *= 51 vs. ALS, *n *= 61: β = 0.689, *p* < 0.001). ALS A+ (*n *= 6), but not ALS A− (*n *= 55), showed higher CSF BD-tau levels than disease controls (*n *= 31; unadjusted *p* = 0.044); however, this finding did not survive multiple-comparison correction (Fig. 1D).

CSF t-tau levels were higher in both AD (*n *= 101) and ALS (*n *= 116) compared with disease controls (*n *= 75; *p* < 0.001 and *p* = 0.032, respectively), with higher levels in the former than in the latter group (*p* < 0.001) (Table 1, Fig. 1E). These findings were confirmed in the individual cohorts (Supplementary Tables 2–5) and after adjustment for age and centre (AD, *n *= 101 vs. disease controls, *n *= 75: β = 0.806, *p* < 0.001; ALS, *n *= 116 vs. disease controls, *n* = 75: β = 0.261, *p* < 0.001; AD, *n* = 101 vs. ALS, *n* = 116: β = 0.698, *p* < 0.001). ALS A+ (*n* = 12), but not ALS A− (*n* = 104), showed higher CSF t-tau levels than disease controls (*n* = 75; *p* = 0.011) (Fig. 1F).

Other biomarker (serum t-tau, serum p-tau181, CSF p-tau181, serum NfL) distribution and comparisons among diagnostic groups in the whole original and in the individual cohorts have been previously described^8^.

Serum and CSF BD-tau showed a moderate correlation in the whole cohort (*n* = 143, Spearman’s r = 0.432, *p* < 0.001), with no association observed in the single diagnostic groups. Strong correlations were observed between CSF BD-tau and CSF t-tau in the whole cohort (*n* = 143, r = 0.963, *p* < 0.001), ALS (*n* = 61, r = 0.902, *p* < 0.001), AD (*n* = 51, r = 0.879, *p *< 0.001), and disease control groups (*n *= 31, r = 0.921, *p* < 0.001). Other correlations between serum and CSF biomarkers in the diagnostic groups are reported in Supplementary Results.

The diagnostic performances of serum and CSF BD-tau in the whole cohort using the direct one-step approach are reported in Supplementary Results whereas that of p-tau217 has been previously described^8^. Generally, direct Simoa measurement of both serum BD-tau and serum p-tau217 did not discriminate ALS from disease controls or AD from ALS with high accuracy, as both conditions yielded elevated serum levels.

### Two-step approach: serum BD-tau and BD-p-tau217 measurements after pre-analytical semi-automated BD-Tau-IP

**Fig. 2:**
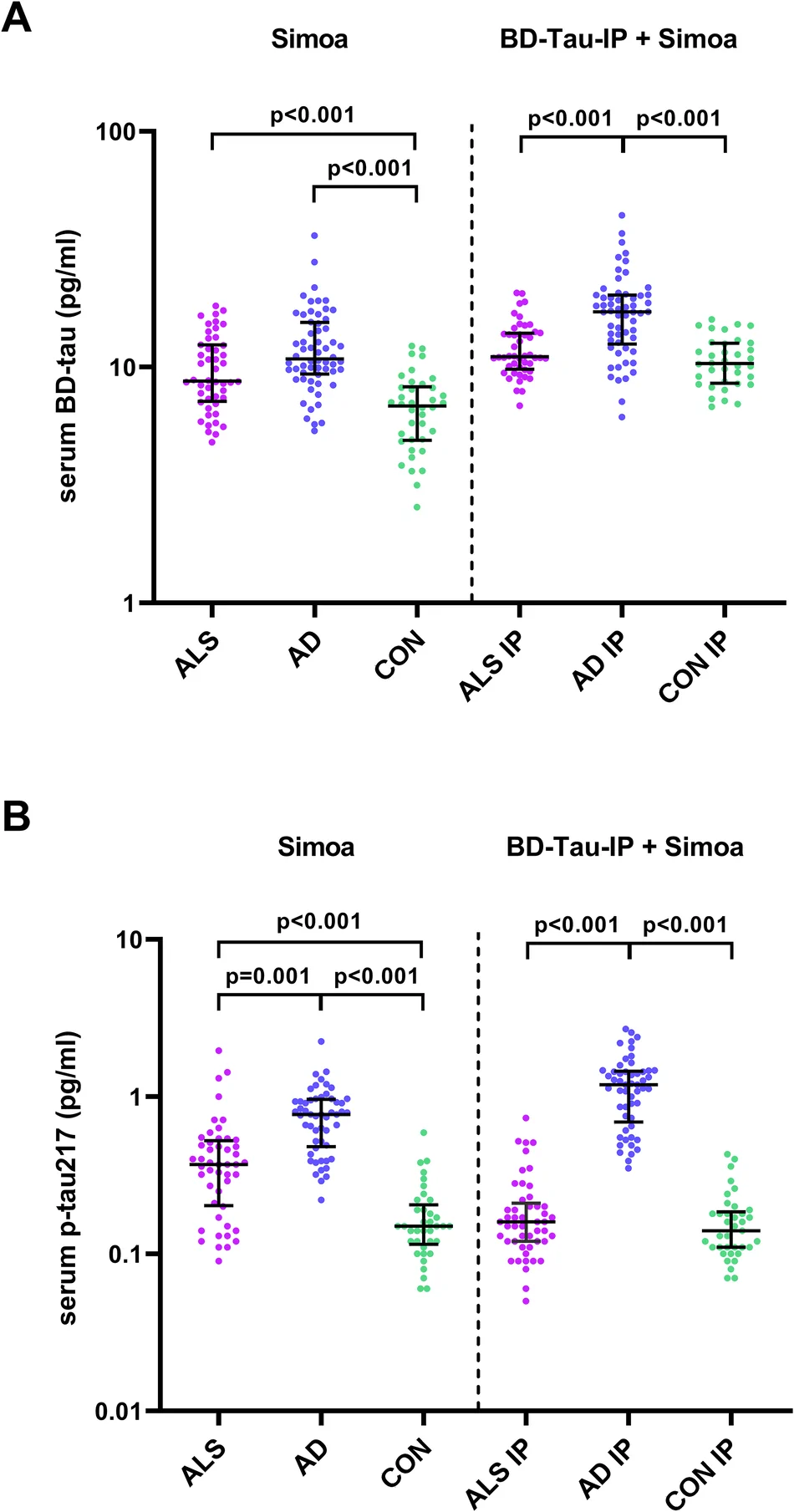
Distribution of serum BD-tau and p-tau217 across diagnostic groups: one-step approach using the Simoa platform vs. two-step approach using semi-automated BD-Tau-IP followed by Simoa. **A**. Distribution of serum BD-tau in a sub-cohort of ALS (*n* = 50), AD subjects (*n* = 60) and disease controls (CON, *n* = 39): one-step approach using the Simoa platform for direct measurement vs. two-step approach using semi-automated BD-Tau-IP followed by Simoa. **B**. Distribution of serum p-tau217 in a sub-cohort of ALS (*n* = 50), AD subjects (*n* = 60) and disease controls (CON, *n* = 39): one-step approach using the Simoa platform for direct measurement vs. two-step approach using semi-automated BD-Tau-IP followed by Simoa. Y-axis shown on a logarithmic scale. Dots represent single data points. Horizontal lines represent the median values, the lower and upper lines correspond to the first and third quartiles, and the vertical line is the interquartile range. Biomarker differences between groups were assessed by Mann–Whitney or Kruskal–Wallis followed by Dunn’s post hoc test (adjustment for multiple comparisons). Two-sided *p*-values are reported. AD Alzheimer’s disease, ALS amyotrophic lateral sclerosis, BD-tau brain-derived tau protein, BD-Tau-IP brain-derived tau immunoprecipitation, CON disease controls, p-tau217 phosphorylated tau protein 217. Source data are provided as a Source Data file.

Serum BD-tau and BD-p-tau217 concentrations after BD-Tau-IP were associated with age but not with sex (Supplementary Results). After semi-automated BD-Tau-IP (*n* = 149), serum levels of BD-tau and BD-p-tau217 were significantly elevated only in the AD group (*n* = 60) compared with ALS (*n* = 50; *p* < 0.001 and *p* < 0.001, respectively) and disease controls (*n* = 39; *p* < 0.001 and *p *< 0.001, respectively), with no significant difference between ALS and disease controls for either biomarker (Table 2, Fig. 2). These findings were replicated even after age-adjustment (AD, *n* = 60 vs. disease controls, *n* = 39: BD-tau, β = 0.482, *p* < 0.001; BD-p-tau217, β = 0.679, *p* < 0.001; ALS, *n* = 50 vs. disease controls, *n* = 39: BD-tau, not significant; BD-p-tau217, not significant; AD, *n* = 60 vs. ALS, *n* = 50: BD-tau, β = 0.310, *p* = 0.003; BD-p-tau217, β = 0.607, *p* < 0.001).

**Table 2:** Serum BD-tau and p-tau217 distribution across diagnostic groups: one-step approach using the Simoa platform vs. two-step approach using semi-automated BD-Tau-IP followed by Simoa

	ALS	AD	Disease controls	*p*-value
*N*	50	60	39	
**Serum BD-tau**				
Simoa (pg/mL) Median (IQR)	8.80 (7.27–13.30)	10.85 (9.34–15.48)	6.95 (4.92–8.48)	<0.001
BD-Tau-IP + Simoa (pg/mL) Median (IQR)	11.13 (9.87–14.03)	17.15 (12.55–20.23)	10.40 (8.60–12.69)	<0.001
Concentration Factor Median (IQR)	1.27 (1.06–1.49)	1.36 (1.18–1.65)	1.60 (1.38–1.83)	<0.001
**Serum p-tau217**				
Simoa (pg/mL) Median (IQR)	0.38 (0.21–0.54)	0.76 (0.39–0.93)	0.15 (0.11–0.22)	<0.001
BD-Tau-IP + Simoa (pg/mL) (BD-p-tau217) Median (IQR)	0.16 (0.12–0.22)	1.11 (0.53–1.44)	0.13 (0.11–0.18)	<0.001
Concentration Factor Median (IQR)	0.42 (0.28–0.71)	1.44 (1.18–1.78)	1.07 (0.67–1.33)	<0.001
**Ratio BD-tau/BD-p-tau217 (BD-Tau-IP + Simoa)** Median (IQR)	72.80 (52.33–92.86)	17.49 (12.04–22.92)	76.82 (64.09–97.67)	<0.001

Biomarkers (serum BD-tau, serum BD-tau after BD-Tau-IP, serum p-tau217, serum BD-p-tau217 and the ratio BD-tau/BD-p-tau217) in the three diagnostic groups are displayed as median and interquartile range (IQR). Two-sided *p*-values of Kruskal–Wallis are reported. Dunn’s post-hoc tests (adjustments for multiple comparisons) to compare biomarker levels: serum BD-tau (Simoa): AD vs. controls *p* < 0.001; AD vs. ALS *p* = 0.091; ALS vs. disease controls *p* < 0.001; serum BD-tau after BD-Tau-IP: AD vs. controls *p* < 0.001; AD vs. ALS *p* < 0.001; ALS vs. disease controls *p* = 0.366; serum p-tau217 (Simoa): AD vs. controls *p* < 0.001; AD vs. ALS *p* = 0.001; ALS vs. disease controls *p* < 0.001; Serum BD-p-tau217: AD vs. ALS *p* < 0.001, ALS vs. controls *p* = 1.000, AD vs. controls *p* < 0.001, ratio BD-tau/BD-p-tau217: AD vs. ALS *p* < 0.001, ALS vs. controls *p* = 1.000, AD vs. controls *p* < 0.001.

*AD* Alzheimer’s disease, *ALS* amyotrophic lateral sclerosis, *BD-p-tau217* brain-derived phosphorylated tau protein 217, *BD-tau* brain-derived tau protein, *BD-Tau-IP* brain-derived tau immunoprecipitation, *IQR* interquartile range, *N* number of cases, *p-tau217* phosphorylated tau protein 217.

Strong correlations were observed between serum concentrations of BD-tau and p-tau217 measured with the one-step approach and the corresponding values with the two-step approach (Supplementary Results).

We calculated the ratio of serum BD-tau and BD-p-tau217 concentrations^17^ obtained from BD-Tau-IP eluates. Participants with AD (*n* = 60) showed significantly lower ratio values compared with both ALS subjects (*n* = 50) and disease controls (*n* = 39; both *p* < 0.001) (Table 2), even after age-adjustment (AD vs. ALS, β = −0.630, *p* < 0.001; AD vs. controls, β = −0.687, *p* < 0.001).

### Comparative diagnostic accuracy: one-step approach vs. two-step approach with semi-automated BD-Tau-IP

Pre-analytical BD-Tau-IP significantly changed the diagnostic accuracy of both BD-tau and serum p-tau217 for discriminating ALS (*n* = 50), AD (*n* = 60) and disease controls (*n* = 39), compared with the one-step approach (Table 3). For serum BD-tau, BD-Tau-IP significantly improved discrimination between AD and ALS (AUC: 0.742 vs. 0.626; DeLong *p* = 0.007) and significantly reduced performance in distinguishing ALS from disease controls (AUC: 0.613 vs. 0.748; DeLong *p* = 0.002), while not significantly affecting AD vs. disease controls discrimination (DeLong *p* = 0.223) (Table 3).

**Table 3:** Comparative analysis of the diagnostic accuracy of serum BD-tau and p-tau217: one-step approach using the Simoa platform vs. two-step approach using semi-automated BD-Tau-IP followed by Simoa

	AUC		
Biomarker	AD vs. ALS	AD vs. controls	ALS vs. controls
**Serum BD-tau**			
Simoa	0.626 ± 0.054	0.851 ± 0.040	0.748 ± 0.052
BD-Tau-IP + Simoa	0.742 ± 0.049	0.817 ± 0.044	0.613 ± 0.062
DeLong test *p*-value	0.007	0.223	0.002
**Serum p-tau217**			
Simoa	0.729 ± 0.050	0.941 ± 0.024	0.799 ± 0.048
BD-Tau-IP + Simoa (BD-p-tau217)	0.900 ± 0.033	0.919 ± 0.032	0.582 ± 0.061
DeLong test *p*-value	<0.001	0.505	0.001
**Ratio BD-tau/BD-p-tau217 (BD-Tau-IP + Simoa)**			
Ratio BD-tau/BD-p-tau217 (BD-Tau-IP + Simoa)	0.961 ± 0.020	0.963 ± 0.020	0.543 ± 0.063
DeLong *p*-value vs. BD-Tau-IP BD-tau	<0.001	<0.001	0.367
DeLong *p*-value vs. BD-p-tau217	0.015	0.199	0.294

*AD* Alzheimer’s disease, *ALS* amyotrophic lateral sclerosis, *BD-p-tau217* brain-derived phosphorylated tau protein 217, *BD-tau* brain-derived tau protein, *BD-Tau-IP* brain-derived tau immunoprecipitation, *p-tau217* phosphorylated tau protein 217.

ROC analyses were performed to calculate the area under the curve (AUC) for the biomarkers (serum BD-tau, serum BD-tau after BD-Tau-IP, serum p-tau217, serum BD-p-tau217 and the ratio BD-tau/BD-p-tau217) when comparing the three diagnostic groups. The DeLong test was used to compare the AUCs. Two-sided *p*-values are reported.

For serum p-tau217, BD-Tau-IP substantially improved the discriminative performance in the comparison AD vs. ALS (BD-p-tau217 AUC: 0.900 vs. 0.729; DeLong *p* < 0.001) and markedly reduced ALS vs. disease controls performance (AUC: 0.582 vs. 0.799; DeLong *p* = 0.001), while leaving AD vs. disease controls accuracy largely unchanged (DeLong p = 0.505) (Table 3).

The BD-tau/BD-p-tau217 ratio (BD-Tau-IP + Simoa) achieved the highest discrimination between AD and ALS (AUC: 0.961 ± 0.020) and was significantly superior to BD-p-tau217 alone in this comparison (DeLong *p* = 0.015) (Table 3). The BD-tau/BD-p-tau217 ratio (BD-Tau-IP + Simoa) generally performed better than BD-tau after BD-Tau-IP alone in most comparisons. However, its performance was not significantly superior to that of BD-p-tau217 alone, except in the comparison between AD and ALS (Table 3).

### Two-step approach with manual BD-Tau-IP and inter-method reproducibility

The results of the two manual BD-Tau-IP runs, including biomarker distributions and detailed diagnostic accuracy comparisons, are reported in the Supplementary Results. In brief, we found a systematic tendency for the manual BD-Tau-IP to yield higher values compared to the semi-automated procedure, which is consistent with known differences in starting volumes, elution volumes and dilution factors between the two methods (Supplementary Results). However, the distribution of serum BD-tau and p-tau217 following manual and semi-automated BD-Tau-IP across diagnostic groups was comparable (Supplementary Tables 7–10 and Supplementary Figs. 1–2). Reproducibility data for the BD-Tau-IP procedure, including inter-method reproducibility between the manual and semi-automated protocols, are reported in detail in the Supplementary Results.

### Associations between tau biomarkers (one-step and two-step approaches) and clinical and laboratory variables

The clinical and laboratory characteristics of ALS patients from the Halle and Milan cohorts are reported in Supplementary Table 11. In the ALS cohort from Halle and Milan (*n *= 123), serum BD-tau (one-step approach) was positively associated with disease progression rate (*n* = 88, r = 0.318, *p* = 0.003) and negatively associated with ALSFRS-R score (*n* = 88, r = −0.471, *p* < 0.001). No associations were observed between BD-tau levels (one-step approach) and BMI, disease duration at blood sampling, site of symptom onset, King’s stage, ECAS score, MoCA score, upper motor neuron (UMN) or lower motor neuron (LMN) burden, or blood levels of CK, troponin, or myoglobin. Moreover, ALS clinical phenotypes did not differ in serum or CSF BD-tau levels. Associations between serum p-tau217 measured with the one-step approach and clinical and laboratory variables in ALS have already been described^8^. Finally, none of the clinical and laboratory characteristics of ALS patients (*n* = 50) was significantly associated with serum BD-tau after BD-Tau-IP or BD-p-tau217 (two-step approach).

### Pre-analytical pan-tau and p-tau217 immunoprecipitation followed by targeted mass spectrometric analyses of BD-tau and big tau in brain, spinal cord and muscle samples

**Fig. 3:**
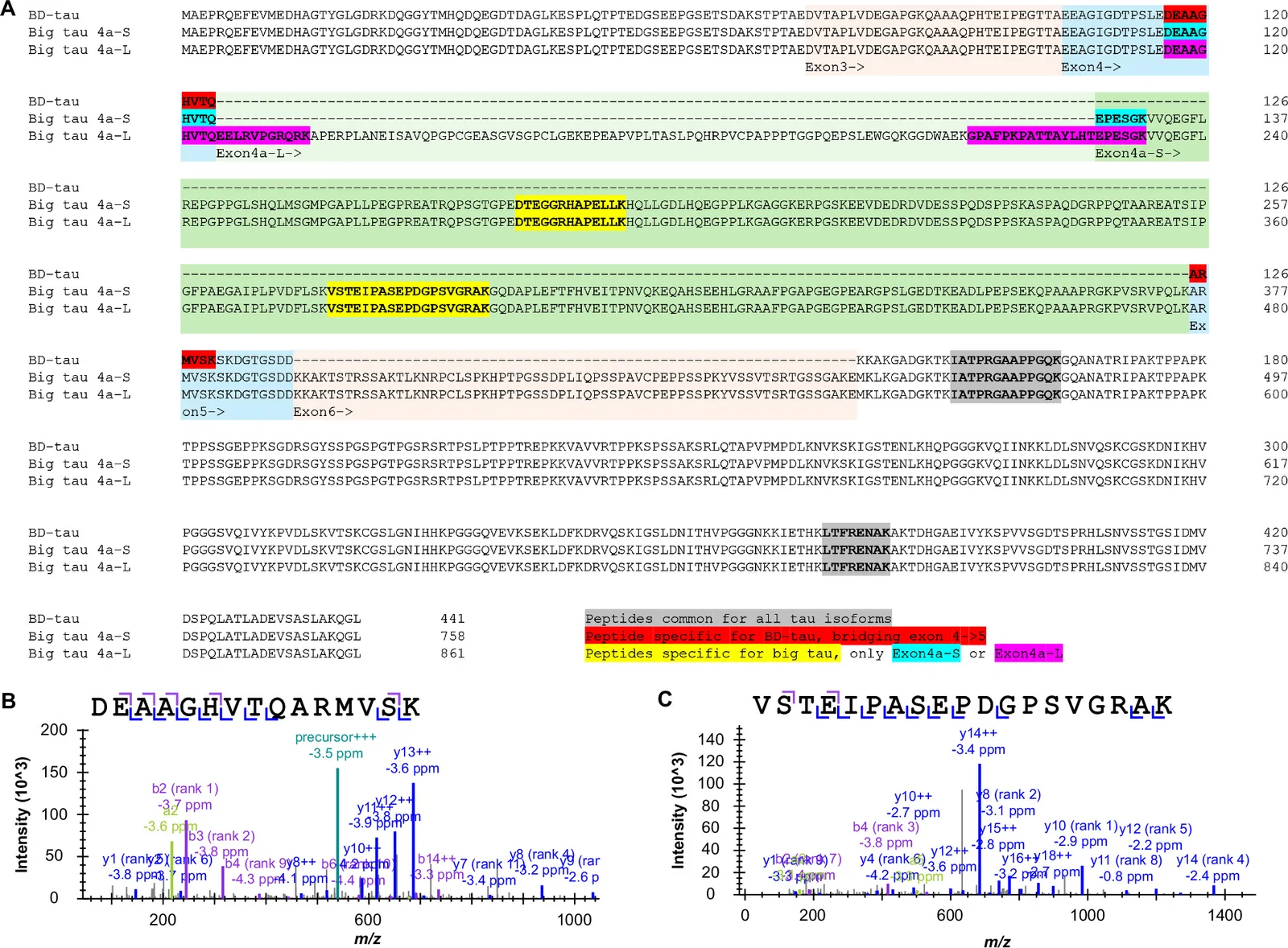
Targeted mass spectrometric analysis of BD-tau and big tau isoforms in distinct tissues and identification of exon 4a-L and exon 4a-S isoforms in muscle tissue by untargeted mass spectrometry. **A** Sequence alignment of BD-tau and of the 2N4R big tau isoforms containing exon 4a-S and exon 4a-L, highlighting the isoform-specific peptides analysed by targeted mass spectrometry (BD-tau in red, big tau in yellow). Peptides common to all tau species and used for normalisation are indicated in grey. Peptides specific to big tau with exon 4a-L and to big tau with exon 4a-S are indicated in magenta and cyan, respectively. The alignment was generated with Clustal Omega^53^. **B** Representative annotated mass spectrum of the BD-tau–specific peptide spanning the exon 4–5 junction (DEAAGHVTQARMVSK), obtained from an AD brain sample (subject No. 1, female, 78 years old with high AD neuropathologic changes). **C** Representative annotated mass spectrum of a big tau–specific peptide located within exon 4a (VSTEIPASEPDGPSVGRAK), obtained from an AD brain sample (subject No. 1). Spectra of the big tau exon 4a-L– and exon 4a-S–specific peptides are shown in Supplementary Fig. 3. AD Alzheimer’s disease, BD-tau brain-derived tau protein, big tau big tau protein, big tau 4a-L big tau exon 4a-L isoform, big tau 4a-S big tau exon 4a-S isoform.

The expression of BD-tau and big tau peptides was analysed after pan-tau-IP followed by targeted mass spectrometry in protein extracts from frontal cortex (AD *n* = 4, ALS *n* = 7, of whom 4 with sporadic ALS and 3 with C9ORF72-associated ALS, disease controls *n* = 5), spinal cord (ALS *n* = 6, disease controls *n *= 5) and muscle (ALS *n* = 5, disease controls *n* = 5). BD-tau was measured using a peptide spanning the exon 4–exon 5 junction, big tau using two peptides from exon 4a, and t-tau using two peptides common to all isoforms, which served for normalisation (Fig. 3A–C).

In addition, untargeted data-dependent mass spectrometry on two pan-tau-IP muscle samples confirmed the co-expression of both big tau exon 4a-L and exon 4a-S isoforms (Fig. 3A and Supplementary Fig. 3). These analyses were designed to establish the presence of both variants and did not permit quantification of their relative abundance.

**Fig. 4:**
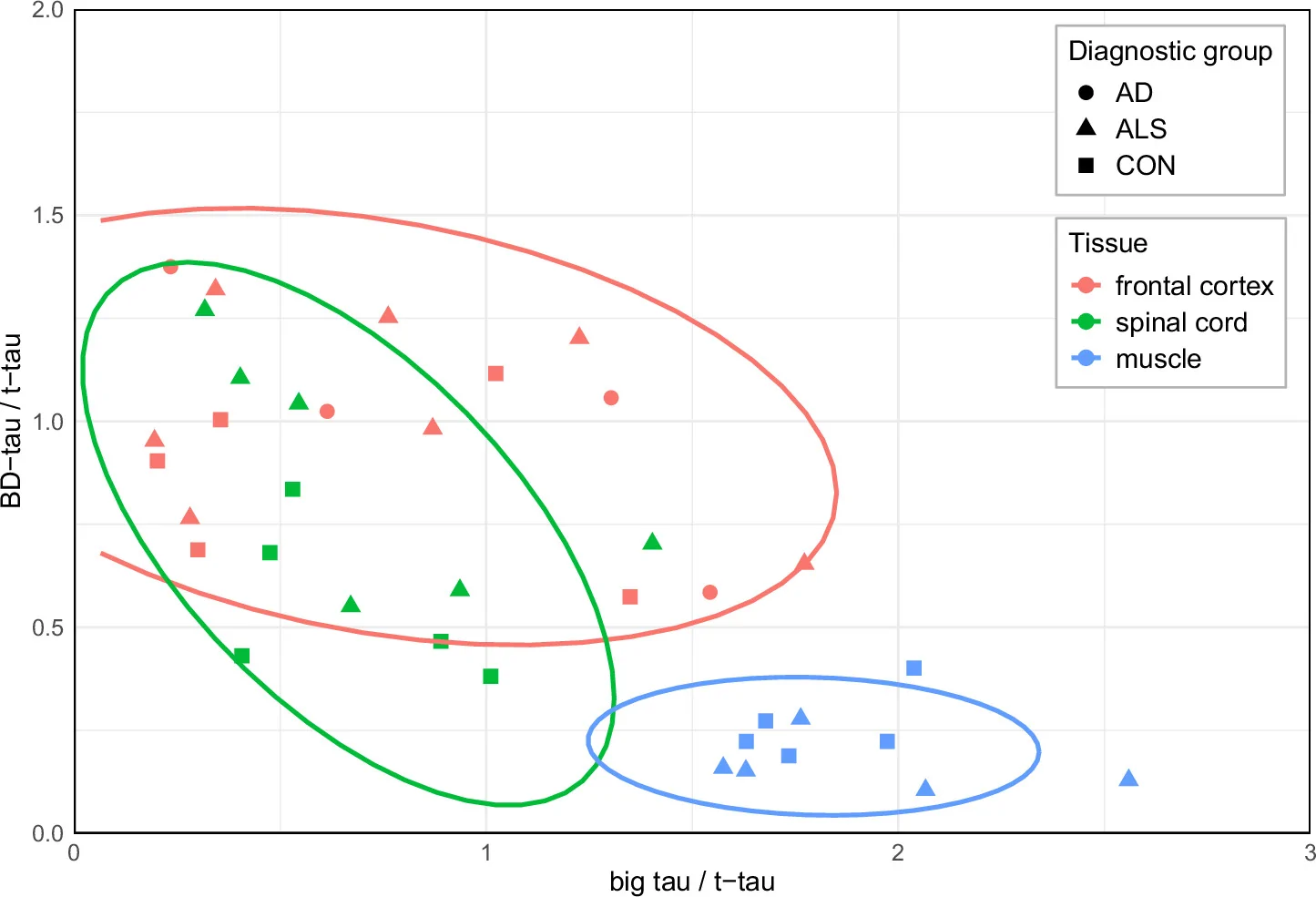
Distribution of BD-tau and big tau relative to total tau across frontal cortex, spinal cord and muscle. Individual BD-tau/t-tau and big tau/t-tau ratios measured by targeted mass spectrometry in samples obtained after pan-tau immunoprecipitation of protein extracts from frontal cortex (AD, *n* = 4; ALS, *n* = 7; CON, *n* = 5), spinal cord (ALS, *n* = 6; CON, *n* = 5) and muscle (ALS, *n* = 5; CON, *n* = 5). BD-tau and big tau–specific peptide intensities were normalised to the intensities of peptides common to all tau isoforms. Each symbol represents an individual sample; symbol shape indicates the diagnostic group (circles, AD; triangles, ALS; squares, disease controls) and colour indicates tissue origin (red, frontal cortex; green, spinal cord; blue, muscle). Ellipses denote 95% confidence regions for each tissue, drawn in the corresponding colour. The shift from the upper left to the lower right of the plot reflects a transition from predominant BD-tau in the central nervous system (frontal cortex and spinal cord) towards predominant big tau in muscle. AD Alzheimer’s disease, ALS amyotrophic lateral sclerosis, BD-tau brain-derived tau protein, big tau big tau protein, CON disease controls, t-tau total tau protein. Source data are provided as a Source Data file.

Using targeted mass spectrometry, both BD-tau and big tau were detected in all pan-tau-IP samples irrespective of tissue and diagnosis. The highest BD-tau/t-tau ratios were found in the frontal cortex, with BD-tau clearly predominating over big tau and a stable BD-tau/big tau ratio across diagnostic groups (Fig. 4). The opposite pattern was observed for big tau/t-tau, which showed the highest values in muscle and the lowest in the frontal cortex. Spinal cord samples showed an intermediate pattern, with a greater relative contribution of big tau than in the frontal cortex, although with considerable overlap between the two CNS tissues (Fig. 4). Median and interquartile range values for both ratios in each tissue are reported in Supplementary Table 12 and Supplementary Fig. 4.

**Fig. 5:**
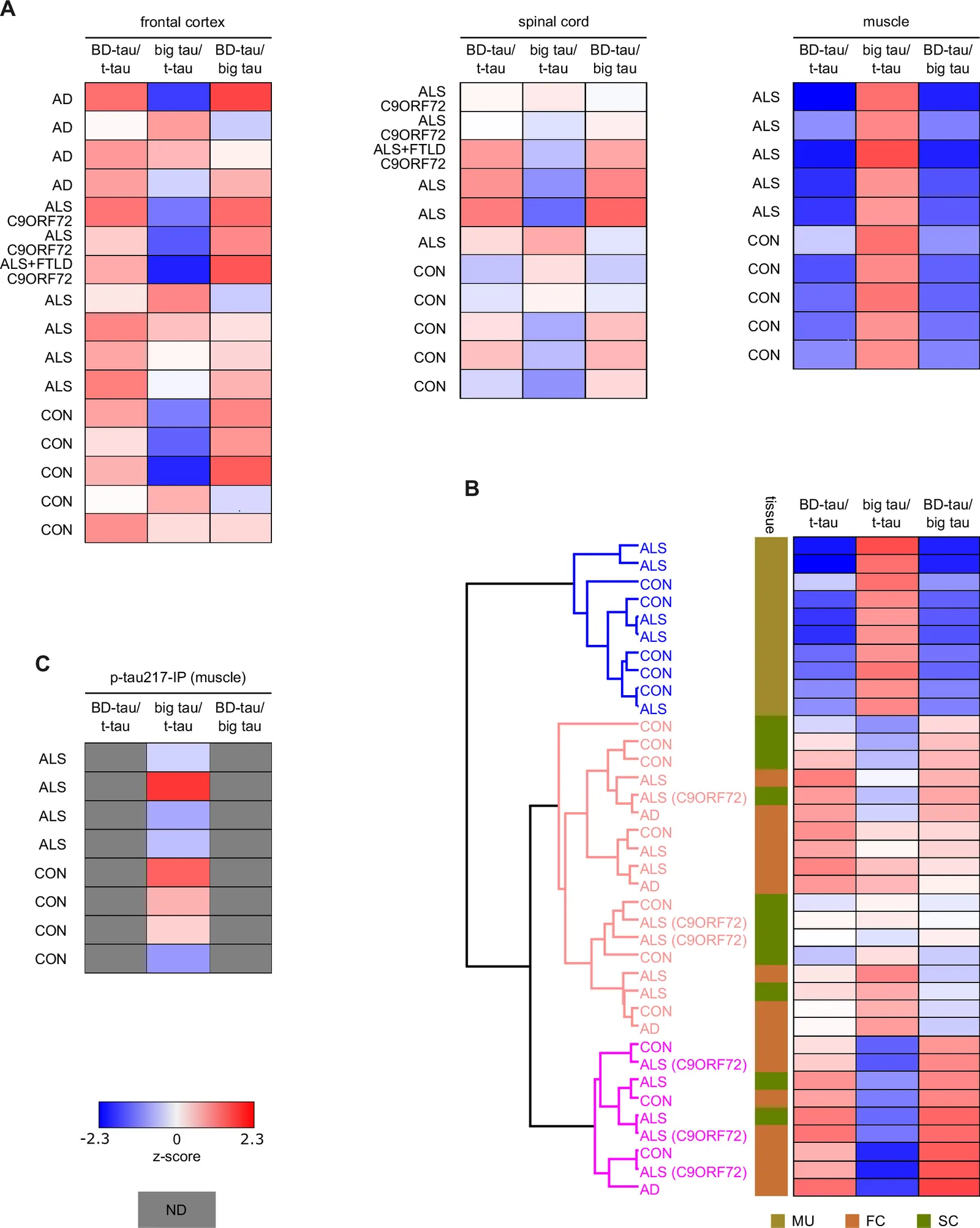
Analysis of BD-tau and big tau isoforms and their phosphorylated variants in frontal cortex, spinal cord, and muscle by immunoprecipitation-targeted mass spectrometry. **A** Relative BD-tau and big tau levels measured by targeted PRM mass spectrometry in protein extracts from frontal cortex (AD *n* = 4, ALS *n* = 7, CON *n* = 5), spinal cord (ALS *n* = 6, CON *n* = 5), and muscle (ALS *n* = 5, CON *n* = 5) following pan-tau immunoprecipitation (pan-tau-IP). PRM intensities for BD-tau and big tau peptides were normalized to t-tau peptide intensities. Additionally, the ratio of BD-tau to big tau was calculated. In frontal cortex, BD-tau predominated over big tau across all diagnostic groups. In muscle, big tau was the dominant species in both ALS and disease controls, with only low BD-tau signal detectable. **B** Hierarchical clustering analysis of BD-tau/t-tau, big tau/t-tau, and BD-tau/big tau ratios across all investigated tissues showed a clear separation of muscle from CNS tissues. **C** Following p-tau217-IP on muscle protein extracts (ALS *n* = 4, CON *n* = 4), only big p-tau217 was detected, whereas BD-p-tau217 was absent in all samples, directly demonstrating that quantitatively relevant T217 phosphorylation in muscle occurs only on big tau isoforms. All ratios were z-scored for visualisation purposes; red indicates above-average and blue indicates below-average ratios. AD Alzheimer’s disease, ALS amyotrophic lateral sclerosis, BD-tau brain-derived tau protein, big tau big tau protein, C9ORF72 chromosome 9 open reading frame 72, CON disease controls, FC frontal cortex, FTLD frontotemporal lobar degeneration, MU muscle, ND not detected, PRM parallel reaction monitoring, p-tau217 phosphorylated tau protein 217, SC spinal cord, t-tau total tau protein. Source data are provided as a Source Data file.

Accordingly, the BD-tau/big tau ratio decreased along a central-to-peripheral gradient, with the highest values in brain and spinal cord and the lowest in muscle (Figs. 4 and 5A). Hierarchical clustering analysis revealed a clear separation of muscle from CNS tissues based on tau isoform ratios (Fig. 5B). Raw intensities for all tau species showed a trend towards higher levels of BD-tau, big tau and t-tau in the frontal cortex of AD patients compared with disease controls and ALS patients, a trend towards higher values of tau species in the spinal cord of ALS patients compared with disease controls, and no significant differences between ALS and disease controls in muscle (Supplementary Fig. 5).

To determine which tau isoforms are phosphorylated at T217 in muscle, p-tau217-IP was performed on muscle protein extracts (ALS *n* = 4, disease controls *n* = 4) followed by targeted mass spectrometry. Only big tau, but not BD-tau, was detected in these samples (Fig. 5C), demonstrating that quantitatively relevant T217 phosphorylation in muscle occurs only on big tau isoforms.

## Discussion

In this study, we combined commercial direct serum and CSF immunoassays (one-step approach), a two-step approach incorporating selective serum BD-Tau-IP, and tissue-based mass spectrometry to distinguish brain-derived from peripheral tau species in AD and ALS.

First, CSF BD-tau mirrored the distribution of CSF t-tau in both ALS and AD, with markedly higher concentrations in AD, consistent with the observation that approximately 99% of CSF total tau consists of BD-tau peptides^12^ and with the strong correlation between the two biomarkers, as previously reported^7^. Although a recent meta-analysis reported elevated CSF t-tau in ALS relative to controls^18^, this did not account for AD co-pathology, present in 10–15% of ALS cases^19^. Indeed, elevated CSF t-tau and BD-tau in our ALS cohort were driven by cases with AD co-pathology, with no increase observed in ALS without AD co-pathology.

Notably, in our cohort, serum BD-tau concentrations, measured using a commercially available Simoa assay, were higher in ALS compared with disease controls, independent of AD co-pathology, though less markedly than in AD. This pattern was consistent with the distribution of serum p-tau217 in the same diagnostic groups^8^. In contrast, Janelidze et al. found a non-significant increase in plasma BD-tau in ALS compared with controls, but no difference between AD and ALS^2^. We also confirmed earlier reports of elevated BD-tau levels in both CSF and blood of AD patients compared with controls, and the strong diagnostic performance of this biomarker for AD pathology^6,7,20^.

Regarding the elevation of serum BD-tau in ALS, one plausible explanation is that the commercial Simoa BD-tau assay may not be sufficiently specific to distinguish between BD- and big tau isoforms. Thus, in ALS, the assay may cross-react with peripheral tau species released from denervated muscle fibres, similar to what has been reported for the commercial Simoa p-tau217 (ALZpath p-Tau 217 Advantage PLUS) assay^2,8^. This interpretation is supported by the lack of significant alterations in CSF BD-tau and CSF t-tau levels in ALS patients without AD co-pathology, indicating no substantial release of BD-tau isoforms from the CNS into the CSF in this condition. Furthermore, in contrast to previous reports^6,7,14^, we did not observe strong correlations between serum BD-tau and CSF BD-tau or CSF t-tau in paired CSF and blood samples.

To address this issue, we investigated whether incorporating a pre-analytical BD-Tau-IP into a two-step approach could enhance the diagnostic performance of the BD-tau and p-tau217 Simoa commercial assays. In contrast to our previous Tau-IP protocol^15,16^, which combined a pan-tau antibody and an anti-BD-tau antibody, the BD-Tau-IP used only the latter antibody. Following the two-step approach, increased serum levels of BD-tau and BD-p-tau217 were detected only in the AD group, with no significant differences between ALS cases and disease controls. This contrasted with direct measurements using the commercial Simoa p-tau217 assay^2,8^, or the Lilly p-tau217 assay, which better discriminated between ALS and AD but still reported elevated plasma p-tau217 levels in ALS versus controls^2^. Together, these observations suggest that both commercial p-tau217 assays, and potentially the commercial Simoa BD-tau assay (BD-tau Advantage PLUS), may cross-react to varying extents with peripheral big tau, including its phosphorylated forms. In this regard, the differing performance observed between the commercial Simoa BD-tau assay and our two-step approach for BD-tau species is most likely attributable to differences in the used antibodies. Although the exact characteristics of the antibody employed in the Simoa BD-tau assay have not been disclosed, the BD-tau antibody (mAb 2B8, Roboscreen), used in our BD-Tau-IP, recognizes an epitope located precisely at the exon 4–exon 5 junction, with half of the binding site spanning the C-terminus of exon 4 and the other half the N-terminus of exon 5. Importantly, the binding of antibody 2B8 is completely inhibited by a recombinant peptide encompassing the exon 4–5 junction, but not by a peptide spanning the exon 4–4a junction, indicating a high specificity for BD-tau^21^.

Overall, our two-step approach selectively measures BD-tau species, including BD-p-tau217, while minimizing the contribution of peripheral big tau forms in serum, thereby reducing false positives arising from muscle tau release. This selectivity, combined with the ability of IP to remove abundant plasma proteins such as albumin and IgG, as well as other substances that may interfere with immunological measurements^15,16^, may account for the improved discrimination between ALS and AD observed with both the Simoa BD-tau and p-tau217 assays after BD-Tau-IP in our ROC analyses. Of note, BD-Tau-IP did not significantly improve the already high performances of the Simoa commercial BD-tau and p-tau217 assays in distinguishing AD from disease controls, but this was expected, as in AD most BD-tau and p-tau217 species in blood are presumed to be CNS-derived.

**Fig. 6:**
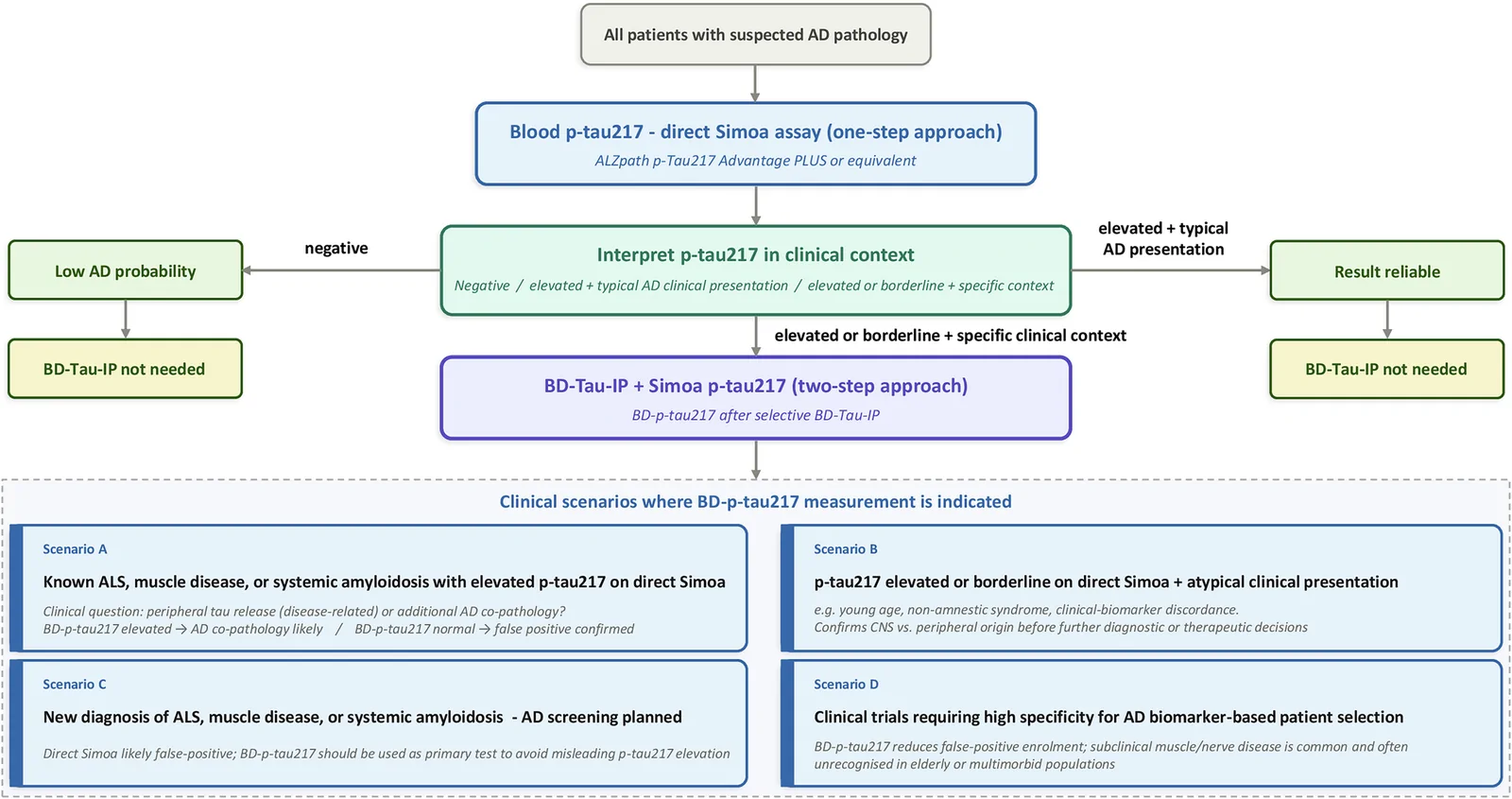
Proposed two-level clinical algorithm for the use of blood p-tau217 and BD-p-tau217 in the diagnostic workup of Alzheimer’s disease. The figure illustrates a pragmatic stepwise decision framework for the sequential use of direct commercial p-tau217 immunoassay (e.g. Simoa, one-step approach) and BD-p-tau217 measurement after pre-analytical BD-Tau immunoprecipitation (two-step approach) in patients with suspected AD pathology. First-line testing relies on direct Simoa quantification of serum p-tau217. A clearly negative result renders AD pathology unlikely and no further tau biomarker testing is required. A clearly positive result in a clinically typical context (i.e. amnestic cognitive impairment without concurrent conditions known to elevate peripheral tau species) is considered reliable without further confirmation with two-step approach. Second-line testing with BD-p-tau217 after BD-Tau-IP is reserved for specific scenarios in which peripheral tau release may confound direct Simoa results: (A) known amyotrophic lateral sclerosis (ALS), muscle disease, or systemic amyloidosis with already elevated serum p-tau217, where the critical question is whether elevation reflects peripheral tau release or genuine AD co-pathology; (B) elevated or borderline p-tau217 with an atypical clinical presentation, such as young age, non-amnestic syndrome, or clinical-biomarker discordance; (C) new diagnosis of ALS, muscle disease, or systemic amyloidosis in whom AD screening is planned, where the one-step approach with Simoa is expected to yield false-positive results; and (D) clinical trials requiring high specificity for AD biomarker-based patient selection, particularly in older or multimorbid populations. In scenarios A–D, a normal BD-p-tau217 after BD-Tau-IP indicates peripheral origin of the blood biomarker elevation, while an elevated result supports genuine AD pathology. AD Alzheimer’s disease, ALS amyotrophic lateral sclerosis, BD-p-tau217 brain-derived phosphorylated tau protein 217, BD-Tau-IP brain-derived tau immunoprecipitation, CNS central nervous system, p-tau217 phosphorylated tau protein 217.

Nevertheless, our two-step approach may help reduce false-positive detection of AD pathology by commercial assays, particularly in elderly or multimorbid individuals with clinical or subclinical muscle or peripheral nerve disease, which are common, often unrecognized, and may increase blood big p-tau species^8,22–25^. Supporting this concern, elevated blood p-tau217 and/or p-tau181 has also recently been reported in systemic amyloidosis and myotonic dystrophy type 1, likely reflecting p-tau release from affected peripheral tissues^24,25^. Moreover, we previously provided evidence that age- or senescence-related processes might influence the release of p-tau peptides from muscle tissue in both control individuals and ALS cases, potentially independent of disease state^8^. Building on these considerations, we propose a pragmatic two-level clinical algorithm (Fig. 6): serum p-tau217 quantification with commercial immunoassays (e.g. Simoa, one-step approach) as a first-line test for all patients with suspected AD pathology, and BD-p-tau217 measurement (two-step approach) as a second-line confirmatory step in specific scenarios, especially where peripheral p-tau release may confound results^8,25^. We acknowledge that the two-step approach is not suitable as a universal first-line test given its pre-analytical complexity and limited availability, and that further standardisation will be required before broader clinical implementation.

In this context, emerging NULISA-based assays offer high-throughput measurement of BD-tau species, with a potential but not consistently significant advantage of BD-p-tau217 over total p-tau217 for AD biomarker classification^26,27^. However, data on non-phosphorylated BD-tau measured by NULISA and validation in ALS or other disorders with peripheral tau release are lacking. Compared with NULISA, our two-step approach requires more sample volume and processing but offers selective pre-analytical enrichment and compatibility with different downstream platforms^15,16^, warranting future head-to-head studies. Despite these limitations, our two-step approach proved robust and reliable, with comparable biomarker distributions across diagnostic groups obtained using both manual and semi-automated BD-Tau-IP protocols, strong correlation between serum BD-p-tau217 concentrations measured with the two protocols and acceptable inter-method reproducibility. Further analytical validation studies will be required to evaluate additional sources of variability, including batch-to-batch differences in antibody-functionalized magnetic beads and other pre-analytical parameters.

Given these new results and our earlier work demonstrating the presence of peripheral p-tau217 peptides in muscle tissue from ALS patients and disease controls^8^, we extended our analysis by performing IP-based tau enrichment prior to targeted mass spectrometry, using both a pan-tau antibody and a p-tau217-specific antibody, to provide a robust characterisation of BD-tau and big tau species as well as their phosphorylated forms across tissues. To this end, we established a cleavage protocol using Asp-N and Lys-C, as conventional tryptic digestion did not robustly cover the junction peptide spanning exon 4 to exon 5 of BD-tau in our pre-experiments testing different enzymes and enzyme combinations. Following pan-tau-IP enrichment and targeted mass spectrometry analysis, both BD-tau and big tau peptides were detected in all tissue samples regardless of diagnosis, with BD-tau clearly predominating over big tau in the brain, in line with the previously reported central-to-peripheral gradient^12^. Moreover, levels of both species showed a trend towards elevation in AD brain, with a stable BD-tau/big tau ratio across diagnostic groups. The spinal cord occupied an intermediate position between the frontal cortex and muscle, consistent with the findings of Koppisetti et al. in peripheral nerve^12^.

Notably, in skeletal muscle big tau was the dominant species in both ALS patients and disease controls, extending the central-to-peripheral gradient to this clinically relevant tissue and indicating a tissue-intrinsic property rather than a disease-specific phenomenon. Untargeted proteomic analyses additionally demonstrated the presence of both exon 4a-L and exon 4a-S big tau isoforms^12^ in muscle but did not permit reliable quantification of their relative abundance, which will require dedicated targeted assays. Critically, p-tau217-IP followed by targeted mass spectrometry on muscle exclusively detected big p-tau217 and no BD-p-tau217, directly demonstrating that quantitatively relevant T217 phosphorylation in muscle occurs only on big tau isoforms.

**Fig. 7:**
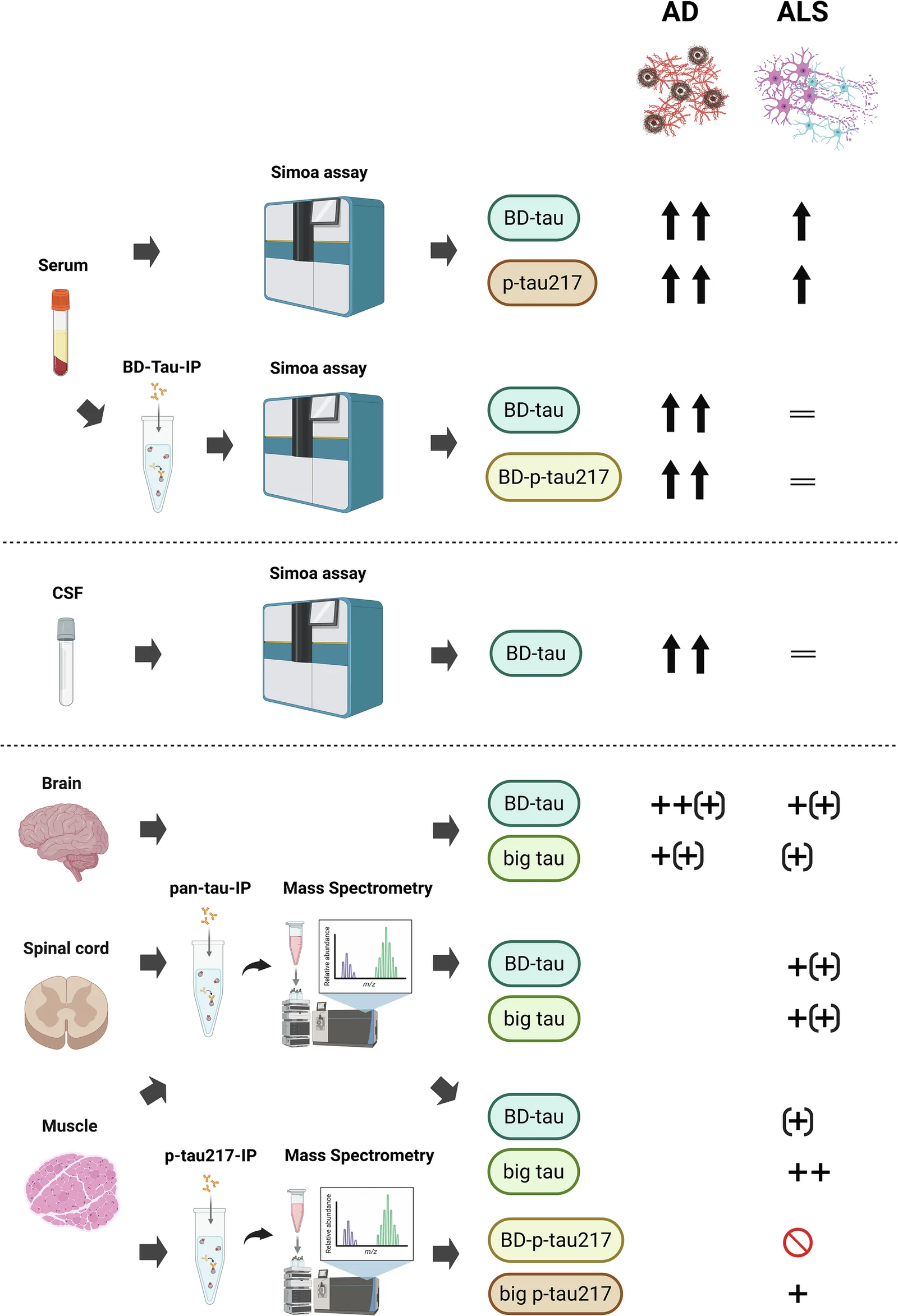
Distinct tau isoform signatures in Alzheimer’s disease and amyotrophic lateral sclerosis. The schematic summarizes the analytical approaches used to detect and validate biomarkers, including immunoassays and targeted mass spectrometry. In AD, neurodegeneration-associated release of BD-tau isoforms, including phosphorylated forms (e.g. BD-p-tau217), into CSF and blood accounts for the markedly elevated CSF and serum BD-tau and serum p-tau217 concentrations. In AD, direct Simoa measurements and BD-Tau-IP-based assays yield concordant results, consistent with a CNS origin of the detected tau species. In contrast, in ALS, direct Simoa assays (one-step approach) show an apparent increase in blood BD-tau and p-tau217 that is not observed following the two-step approach, indicating an analytical artefact driven by cross-reactivity of the assays with big tau species of muscular origin. Indeed, BD-tau is expressed at only minimal levels in muscle, where big tau isoforms predominate; accordingly, the minimal BD-tau fraction present in muscle is not significantly phosphorylated at T217, whereas big tau carries T217 phosphorylation. Consistent with this interpretation, brain and CSF BD-tau are not increased in ALS compared with  disease controls. Spinal cord and muscle samples were available only from ALS patients and disease controls; AD samples were not available for these tissues. Symbol legend: ↑↑ markedly elevated vs. controls, ↑ moderately elevated vs. controls, = no significant difference vs. controls, ++(+) moderate abundance with trend towards higher levels in a limited sample size, +(+) low abundance with trend towards higher levels or with considerable overlap between tissue compartments, + low abundance, (+) trace levels detectable; not detected. Symbols in parentheses indicate preliminary findings requiring confirmation in larger cohorts. AD Alzheimer’s disease, ALS amyotrophic lateral sclerosis, BD-p-tau217 brain-derived phosphorylated tau protein 217, BD-tau brain-derived tau protein, BD-Tau-IP brain-derived tau immunoprecipitation, big tau big tau protein, big p-tau217 big phosphorylated tau protein 217, CSF cerebrospinal fluid, pan-tau-IP total tau protein immunoprecipitation, p-tau217 phosphorylated tau protein 217, p-tau217-IP phosphorylated tau protein 217 immunoprecipitation. Created in BioRender. Abu Rumeileh, S. (2026) https://BioRender.com/ktr5mh3.

These patterns observed across tissues are largely concordant with our findings in blood and CSF. In ALS, our mass spectrometry data do not show increased BD-tau levels in brain tissue compared with controls, consistent with the absence of increased BD-tau release into biofluids, as reflected by unchanged BD-tau levels in CSF and, after BD-Tau-IP, in serum (Fig. 7). Moreover, although low BD-tau is detectable in muscle after mass spectrometric analysis, this fraction is not detectably phosphorylated at T217 and contributes negligibly to the total tau pool. Thus, elevated serum BD-tau in ALS measured by commercial Simoa assay most likely reflects cross-reactivity with peripheral big tau and, to a lesser extent, BD-tau released from muscle fibres, while elevated serum p-tau217 reflects big p-tau217 from the same source (Fig. 7). Although the convergent tissue-specific mass spectrometry and biomarker findings strongly support a peripheral origin of the serum p-tau217 signal in ALS, direct proteomic assessment of BD-p-tau217 and big p-tau217 species in serum remains an important objective for future studies.

An important question is whether elevated serum BD-tau in ALS measured with commercial assays reflects, at least in part, spinal cord motor neuron degeneration rather than peripheral muscle origin. The most compelling argument against this is that serum BD-tau and BD-p-tau217 after BD-Tau-IP are not elevated in ALS versus disease controls. If spinal cord degeneration were a substantial source of BD-tau release, some significant elevation would be expected after BD-Tau-IP in ALS, which was not observed. Of note, our mass spectrometry data show a trend towards higher tau levels in the spinal cord of ALS patients, which however does not translate into detectable BD-tau elevation in blood after BD-Tau-IP. Residual BD-tau and BD-p-tau217 detectable after the two-step approach in both ALS and disease controls likely reflect physiological baseline release from the CNS. For blood BD-tau, a minor muscle contribution cannot be excluded; BD-p-tau217, by contrast, originates exclusively from the CNS, since muscle BD-tau is not detectably phosphorylated at T217.

In AD, increased release of BD-tau isoforms, including phosphorylated forms (e.g. BD-p-tau217), into CSF and blood^12,20^, accounts for the markedly elevated serum and CSF BD-tau and serum BD-p-tau217 concentrations observed in our study(Fig. 7). Collectively, these observations reinforce the concept that extracellular BD-tau release is associated with AD pathological changes. Nevertheless, elevated blood BD-tau levels have also been reported in conditions involving acute brain injury, such as traumatic brain injury and stroke^28,29^.

On another issue, we found inconsistent associations between serum BD-tau and clinical variables in ALS. While serum BD-tau measured by direct Simoa assay correlated with disease severity and progression rate, these associations were not confirmed after BD-Tau-IP, suggesting they reflect peripheral tau release from degenerating muscle fibres rather than neuroaxonal degeneration. This is further supported by the contrast with NfL, an established marker of neuroaxonal damage, which is robustly associated with disease severity, progression, and therapy response in ALS^30^.

Distinguishing BD-tau from peripheral tau species may also be clinically relevant in ALS. Previous studies have associated blood p-tau with lower motor neuron (LMN)-predominant phenotypes, denervation as well as muscle pathology^2,8,31–34^. Big p-tau-specific assays may therefore support phenotypic stratification, non-invasive assessment of denervation burden and longitudinal disease monitoring in ALS, although such assays remain to be developed and validated against clinical and electrophysiological variables. Conversely, BD-tau-selective assays may improve recognition of concomitant AD pathology in ALS (Fig. 6).

This study has several notable strengths, including the use of multiple biospecimen types (serum, CSF, brain, spinal cord and muscle), the integration of ultrasensitive ELISAs, immunoprecipitation assays, and mass spectrometry, and its multicentre design. A limitation is that participating centres used slightly different CSF biomarker cut-offs to classify AT(N) status^8^. Nevertheless, all CSF biomarkers were measured using identical assays and platforms across centres^8^, and our findings remained consistent after adjusting for centre. Further limitations include the smaller sample size for the two-step approach and CSF BD-tau analyses compared with the direct Simoa serum measurements. We also acknowledge the limited neuropathological material, absence of peripheral nerve tissue, and lack of paired biospecimens^8^. Mass spectrometry findings should therefore be considered preliminary mechanistic evidence supporting the biofluid marker observations rather than definitive conclusions on tau isoform tissue distribution. A prolonged post-mortem interval (>48 h) in a subset of donors may additionally have affected tau protein integrity. Larger post-mortem studies are needed to confirm and extend our findings. Furthermore, our mass spectrometry approach for BD-tau/big tau discrimination has been validated in CNS and muscle tissue but not yet in biofluids, where tau abundance is very low and proteome complexity and dynamic range are high. Future quantitative targeted mass spectrometry approaches, potentially combined with isotopically labelled reference peptides (e.g. AQUA peptides) and improved immunoenrichment strategies, may enable absolute quantification of BD-tau and BD-p-tau217 in biofluids and tissues, supporting assay standardisation across laboratories and longitudinal monitoring in clinical studies. In addition, we acknowledge the absence of renal function data; however, previous studies indicate that renal function may influence p-tau217 levels, but only minimally affects BD-tau concentrations in blood^7,35^.

In conclusion, our findings clarify the distinct biological origins of BD-tau and big tau across different biofluids, tissues and disease states. While commercially available Simoa assays for BD-tau and p-tau217 are susceptible to interference from big tau in ALS and potentially other conditions involving muscle damage, BD-tau and BD-p-tau217 quantified in blood after pre-analytical BD-Tau-IP offer a highly specific readout of CNS-derived tau, including its phosphorylated forms, reliably distinguishing AD pathology from peripheral tau release. Our combined immunoprecipitation and targeted mass spectrometric analysis further confirmed the distinct tissue distributions of BD-tau and big tau, providing strong biological support for selective enrichment strategies. Although further standardisation is required before clinical implementation, our findings highlight the promise of BD-tau and BD-p-tau217 measured with the two-step approach as reliable blood biomarkers for identifying AD pathology, with important implications for patient screening and treatment selection in the era of disease-modifying therapies.

## Methods

### Inclusion and ethics

This research complies with all relevant ethical regulations. The study was conducted according to the revised Declaration of Helsinki and Good Clinical Practice guidelines. Informed written consent was obtained from participants and/or their relatives. The study of biosamples and case data was approved by the ethics committees of the Martin-Luther-University Halle-Wittenberg (approval number 2021-101), IRCCS Istituto Auxologico Italiano (approval number 2023_03_21_18), University Medicine Mannheim (Division for Neurodegenerative Diseases, approval number 2017-589N-MA and Department of Geriatric Psychiatry, approval number 2012-254N-MA) and University of Ulm (approval numbers 20/10 and 54/08). The study followed the STROBE statement guidelines.

### Case selection for blood and CSF biomarker analyses

This is a retrospective multicentre cohort study including 139 subjects with ALS, 101 with AD continuum (dementia *n* = 77 or mild cognitive impairment *n* = 24), 75 disease controls, and 22 healthy controls, recruited at four European centres: the Department of Neurology, Martin Luther University Halle-Wittenberg, Halle (Saale), Germany (Halle cohort, *n* = 147, plus 22 healthy controls); the Department of Neurology and Laboratory of Neuroscience, IRCCS Istituto Auxologico Italiano, Milan, Italy (Milan cohort, *n* = 90); the Division for Neurodegenerative Diseases, Neurology Department, University Medicine Mannheim, Mannheim, Germany (Mannheim cohort 1, *n *= 28); and the Department of Geriatric Psychiatry, Central Institute of Mental Health, Mannheim, Germany (Mannheim cohort 2, *n* = 50). All cases were included in a previously published multicentre study^8^. Case selection was based on the availability of serum samples from subjects with the target conditions and complete information required for the application of diagnostic criteria. CSF samples were available for all AD and disease control participants and for 116 of the 139 ALS cases. The distribution of diagnostic groups across cohorts is reported in the Supplementary Methods.

ALS diagnosis was based on the Revised El Escorial criteria^36^. The diagnosis of AD dementia or mild cognitive impairment was supported by the analysis of CSF core biomarkers, according to the National Institute on Aging–Alzheimer’s Association recommendations^37,38^. Selection criteria for disease controls and healthy controls are reported in our previous work^8^ and in detail in the Supplementary Methods.

Clinical and demographic data, clinical scores, and laboratory results were collected, when available, for ALS cases from the Halle and Milan cohorts^8^ (*n* = 123), including age at blood sampling, sex, body mass index (BMI), disease duration at blood sampling (time between symptom onset and blood sampling), site of symptom onset^39,40^, clinical phenotype^40,41^, King’s stage at blood sampling (disease staging system)^42^, ALS Functional Rating Scale–Revised (ALSFRS-R) score^40^, disease progression rate at blood sampling^40^, Montreal Cognitive Assessment (MoCA) score^43^, Edinburgh Cognitive and Behavioural ALS Screen (ECAS) total score at blood sampling^44^, and blood levels of creatine kinase (CK), troponin T, and myoglobin. Upper motor neuron (UMN) and LMN burden was assessed by examining for the presence or absence of signs at each spinal level (bulbar, cervical, thoracic, and lumbosacral regions; UMN thoracic was not assessed) as previously reported^31^.

Pathogenic mutations in TARDBP, FUS, SOD1, and ATXN2, as well as chromosome 9 open reading frame 72 (C9ORF72) hexanucleotide repeat expansions, were identified in six, one, one, one, and two cases, respectively. Further details on ALS patients are reported in the Supplementary Methods.

### Blood and CSF biomarker analyses using one-step and two-step approaches

Serum samples were collected at each centre, aliquoted, and stored at −80 °C according to standard procedures. CSF samples were obtained by lumbar puncture, centrifuged in cases of blood contamination, aliquoted, and stored in polypropylene tubes at −80 °C until analysis at each centre.

First, serum and CSF BD-tau and serum p-tau217 were measured using commercially available immunoassays (one-step approach) on the Simoa platform (BD-Tau Advantage PLUS Item 104797; ALZpath p-Tau217 Advantage PLUS kit Item 104570; Quanterix, Billerica, MA, USA). Serum BD-tau was tested in all 337 participants, CSF BD-tau in 143 individuals from the Halle and Milan cohorts (61 with ALS, 51 with AD, and 31 disease controls). These analyses were performed at the Department of Neurology in Halle (Saale). Serum p-tau217 analyses have already been described^8^.

Serum BD-tau and BD-p-tau217 were measured in subgroups of the whole cohort using manual and semi-automated two-step approaches. Both protocols shared the same core principle: a phosphorylation-insensitive BD-tau antibody (mAb 2B8, catalogue number 847-010200760, Roboscreen GmbH, Leipzig, Germany), recognising an epitope at the exon 4–exon 5 junction of tau (amino acids 121–128 of 2N4R tau)^15,16^, coupled to Dynabeads™ M-270 Epoxy Magnetic Beads (Thermo Fisher Scientific, USA), selectively captured BD-tau isoforms from serum during a 1-hour binding incubation at room temperature, followed by sequential washing and heat-denaturation elution (99 °C). Resulting eluates from manual and semi-automated approaches were used for downstream BD-tau and/or p-tau217 quantification by commercial Simoa assays. BD-Tau-IP was performed at Roboscreen GmbH, Leipzig, and Simoa measurements at the Department of Neurology, Halle.

The two protocols differed in starting serum volume, elution volume, dilution factors, and degree of automation (Supplementary Methods and Supplementary Table 13). In the manual protocol, all handling steps were performed by the operator using standard laboratory equipment. In the semi-automated protocol, the same immunoprecipitation principle was implemented on a CyBio FeliX liquid handling instrument (Analytik Jena, Germany) in a 96-deepwell plate format, and all pipetting, bead addition, washing, and elution steps were performed automatically enabling higher throughput and reduced operator-dependent variability.

The first manual BD-Tau-IP and subsequent BD-tau and p-tau217 measurements were performed in 21 ALS cases, 22 AD cases, and 21 controls from the Halle cohort. The second manual BD-Tau-IP and subsequent p-tau217 analyses were performed in 45 ALS cases, 39 AD cases, and 14 controls from the Halle and Milan cohorts. All cases included in the second manual IP were also included in the semi-automated IP. Semi-automated BD-Tau-IP followed by BD-tau and p-tau217 measurements was performed in 50 ALS cases, 60 AD cases, and 39 controls from the Halle, Milan, and Mannheim 2 cohorts.

These procedural differences result in distinct absolute biomarker concentrations across protocols, precluding direct numerical comparison. Moreover, as previously described for analogous two-step approaches^45^, neither absolute recovery of BD-tau and BD-p-tau217 following BD-Tau-IP nor true serum concentrations are known at this stage. Accordingly, BD-tau and BD-p-tau217 concentrations measured in serum IP eluates should be interpreted as relative serum levels.

Results from manual and semi-automated runs are reported separately throughout. The semi-automated run, being the largest (*n* = 149) and most representative across cohorts, is included in the main text. Serum p-tau217 measured after BD-Tau-IP is referred to throughout as BD-p-tau217.

Serum concentrations of phosphorylated tau 181 (p-tau181), total tau (t-tau), and neurofilament light chain (NfL) were all available from a previous study^8^ and were measured at the Department of Neurology in Halle (Supplementary Methods). The p-tau217 Simoa assay (ALZpath p-Tau217 Advantage PLUS kit) has been commercialized for use in blood. Indeed, when applied to CSF, the same assay failed to yield reliable measurements due to matrix effects; therefore p-tau217 analyses were not performed in CSF^8^. CSF AD core biomarkers (A: Aβ42/Aβ40; T: p-tau181; N: t-tau) in the Halle and Mannheim 1 cohorts were measured in Halle (Saale), whereas CSF analyses for the remaining cohorts were performed at the respective centres. Further details are provided in the Supplementary Methods. Pathological cut-off values for CSF AD biomarkers have been previously described^8^. All AD participants and disease controls showed A + T + N+ and A − T − N − CSF profiles, respectively, according to the AT(N) classification system^37^. Accordingly, ALS cases were classified as having AD co-pathology (ALS A+) in the presence of A + T ± CSF profiles^8^. For all biomarker measurements, the coefficients of intra-assay and inter-assay variability were <10% and <15%, respectively.

### Brain, spinal cord and muscle sample collection

Frozen brain samples from the frontal cortex of participants with ALS (4 with sporadic ALS; 3 with C9ORF72-associated ALS, one of whom also with frontotemporal lobar degeneration) or AD (*n* = 4), all lacking significant co-pathologies, as well as from non-neurodegenerative disease controls (*n* = 5), were obtained from the Department of Pathology at Ulm University Hospital, Ulm, Germany. For a subset of 6 ALS cases and 5 disease controls, paired spinal cord samples (cervical or thoracic spinal cord) were additionally available and included in the analysis. Detailed neuropathological classification criteria as well as demographic and neuropathological information of the included cases are provided in Supplementary Methods and in the Supplementary Table 14.

Muscle biopsies were obtained at the Department of Neurology, Halle (Saale), as previously described^8^. Muscle samples were collected from five ALS patients and five disease controls. The control group comprised individuals who had undergone diagnostic evaluation for myalgia, cramps, or exercise intolerance but showed no pathological findings on comprehensive clinical examination, electromyography, or myohistological assessment (Supplementary Table 15). All muscle biopsy samples had been included in our previous study^8^.

Because paired serum, CSF, brain, spinal cord and muscle samples were not available for the same individuals, it was not possible to assess direct associations between biofluid biomarker levels and BD-tau and big tau content in brain, spinal cord or muscle tissue.

### Pan-tau and p-tau217 immunoprecipitations followed by targeted mass spectrometry analysis of BD-tau and big tau

Pan-tau-IP was performed on protein extracts from frontal cortex (AD *n* = 4, ALS *n* = 7, disease controls *n* = 5), spinal cord (ALS *n* = 6, disease controls *n* = 5) and muscle (ALS *n* = 5, disease controls *n* = 5). Given the expected markedly lower tau concentration in peripheral tissues (muscle) compared with CNS tissues^12,46,47^, the amount of total protein used as starting material for pan-tau-IP was adjusted accordingly based on published tau concentration estimates across tissues^12,46,47^: 50 µg for brain, 240 µg for spinal cord, and 1000 µg for muscle samples. For the pan-tau-IP we used functionalized magnetic beads with pan-tau antibody 7E5 directed against the amino acid sequence RGAAPPGQKGQA of 2N4R Tau (catalogue number 847-0102006301, Roboscreen GmbH, Leipzig, Germany) as described^15,16^.

P-tau217-IP was performed on total protein from muscle protein extracts only (ALS *n* = 4, controls *n* = 4) due to limited sample availability. For p-tau217-IP, a mean of approximately 1000 µg of total protein per sample was used. For this IP we used functionalized magnetic beads with p-tau217 polyclonal antibody (#44-744, Thermo Fisher Scientific, Invitrogen, USA). The specificity of this antibody has been confirmed and its successful application in post-mortem AD brain tissue has been demonstrated^48,49^. Further details concerning pan-tau-IP and p-tau217-IP methods are reported in Supplementary Methods.

IP eluates were then analysed by means of targeted mass spectrometry for the detection of BD-tau and big tau.

Eluates from pan-tau-IP and p-tau217-IP were subjected to a two-step proteolytic digestion using Asp-N and Lys-C in a paramagnetic bead–based workflow. This protocol was established because a classical tryptic digestion did not robustly cover the junction peptide spanning exon 4 to exon 5 of BD-tau in our pre-experiments. The resulting peptides were analysed by targeted mass spectrometry using parallel reaction monitoring (PRM), focusing on the following peptide sequences: “DEAAGHVTQARMVSK”, which is specific to BD-tau isoforms; “VSTEIPASEPDGPSVGRAK” and “DTEGGRHAPELLK”, which are specific to big tau isoforms containing exon 4a; “IATPRGAAPPGQK” and “LTFRENAK”, which are common to all tau isoforms (Fig. 3 and Supplementary Table 16). Of note, the two big tau-specific peptides are located within the exon 4a sequence shared between exon 4a-long (4a-L) and exon 4a-short (4a-S) isoforms, as described by Koppisetti et al. ^12^.

Liquid chromatography–tandem mass spectrometry (LC–MS/MS) analyses were performed using an EASY-nLC 1200 system coupled to an Orbitrap Fusion Lumos Tribrid mass spectrometer via a FAIMS Pro DUO interface (all from Thermo Fisher Scientific, USA). Raw liquid chromatography-tandem mass spectrometry data were processed using Skyline software (v25.1.0.237, MacCoss Laboratory, University of Washington, Seattle, WA, USA)^50^. Additional methodological details are provided in the Supplementary Methods.

To investigate the expression of exon 4a-L and exon 4a-S isoforms, two pan-tau-IP muscle eluates were additionally analysed by untargeted, data-dependent acquisition (DDA) mass spectrometry, incorporating the exon 4a-L sequence described by Koppisetti et al.^12^ into the protein database search space. Further details are provided in the Supplementary Methods.

### Statistics and reproducibility

Statistical analyses were performed using IBM SPSS Statistics version 21 (IBM, Armonk, NY, USA), GraphPad Prism version 7 (GraphPad Software, La Jolla, CA, USA), and R software version 4.0.2 (R Foundation for Statistical Computing, Vienna, Austria). Depending on data distribution, variables are presented as percentages, mean ± standard deviation (SD), or median and interquartile range (IQR). The Chi-squared test was used for categorical variables. For continuous variables, depending on data distribution and the number of groups, the Mann–Whitney U test, Student’s *t* test, Kruskal–Wallis test (followed by Dunn’s post hoc testing), or analysis of variance (ANOVA; followed by Tukey’s post hoc test) was applied. All reported *p* values were adjusted for multiple comparisons.

Multivariate linear regression models were used to adjust group comparisons of blood and CSF biomarkers for covariates after natural logarithmic transformation of the dependent variables. Spearman’s correlation coefficients and uni- or multivariate regression analyses were performed to assess associations between variables. Diagnostic accuracy for each biomarker was evaluated using receiver operating characteristic (ROC) curve analyses, and areas under the curve (AUCs) were compared using the DeLong test. All statistical tests were two-tailed, and *p* values < 0.05 were considered statistically significant. No statistical method was used to predetermine sample size^8^. No data were excluded from the analyses. All biomarker analyses were randomized. Investigators were blinded to sample allocation and diagnosis during biomarker measurements and mass spectrometry analyses.

### Reporting summary

Further information on research design is available in the Nature Portfolio Reporting Summary linked to this article.

## Supplementary information

Supplementary Information

Transparent Peer Review file

Reporting Summary

## Source data

Source data

## Data Availability

The biomarker data underlying all figures and tables in this study are provided in the Source Data file. Source data are provided with this paper. Mass spectrometry data generated in this study have also been deposited in the ProteomeXchange Consortium database via the PRIDE partner repository under the identifier PXD079838 (pan-tau-IP-PRM) [http://proteomecentral.proteomexchange.org/cgi/GetDataset?ID=PXD079838], PXD079878 (p-tau217-IP-PRM) [http://proteomecentral.proteomexchange.org/cgi/GetDataset?ID=PXD079878] and PXD079895 (pan-tau-IP untargeted) [http://proteomecentral.proteomexchange.org/cgi/GetDataset?ID=PXD079895]^51,52^. Individual-level clinical data are under restricted access because they contain patient information that cannot be fully anonymised under the EU General Data Protection Regulation and the terms of the ethics approvals. Access can be requested in writing from the corresponding author (markus.otto@uk-halle.de); requests will be reviewed by the investigators and the respective institutions, answered within eight weeks, and granted for non-commercial academic research for ten years following publication. Source data are provided with this paper.

## References

[1] van Dyck, C. H. et al. Lecanemab in early Alzheimer’s disease. *N. Engl. J. Med.* **388**, 9–21 10.1056/NEJMoa2212948 (2023). 36449413

[2] Janelidze, S. et al. A comparison of p-tau assays for the specificity to detect tau changes in Alzheimer’s disease. *Alzheimers Dement* **21**, e70208 10.1002/alz.70208 (2025). PMC12035549 40289884

[3] Therriault, J. et al. Blood phosphorylated tau for the diagnosis of Alzheimer’s disease: a systematic review and meta-analysis. *Lancet Neurol.* **24**, 740–752 10.1016/S1474-4422(25)00227-3 (2025). 40818474

[4] Ashton, N. J. et al. The Alzheimer’s Association Global Biomarker Standardization Consortium plasma phospho-tau Round Robin study. *Alzheimers Dement* **21**, e14508 10.1002/alz.14508 (2025). PMC11851162 39907496

[5] Bali, D. et al. Comparison of plasma ALZpath p-Tau217 with Lilly p-Tau217 and p-Tau181 in a neuropathological cohort. *Acta Neuropathol. Commun.* **13**, 144 10.1186/s40478-025-02064-2 (2025). PMC12207803 40588775

[6] Gonzalez-Ortiz, F. et al. Brain-derived tau: a novel blood-based biomarker for Alzheimer’s disease-type neurodegeneration. *Brain* **146**, 1152–1165 10.1093/brain/awac407 (2023). PMC9976981 36572122

[7] Gonzalez-Ortiz, F. et al. Plasma brain-derived tau is an amyloid-associated neurodegeneration biomarker in Alzheimer’s disease. *Nat. Commun.* **15**, 2908 10.1038/s41467-024-47286-5 (2024). PMC10995141 38575616

[8] Abu-Rumeileh, S. et al. Phosphorylated tau 181 and 217 are elevated in serum and muscle of patients with amyotrophic lateral sclerosis. *Nat. Commun.* **16**, 2019 10.1038/s41467-025-57144-7 (2025). PMC11882981 40044663

[9] Thomas, E. V. et al. ALS plasma biomarkers reveal neurofilament and pTau correlate with disease onset and progression. *Ann. Clin. Transl. Neurol.* **12**, 714–723 10.1002/acn3.70001 (2025). PMC12040516 39913612

[10] Fischer, I. & Baas, P. W. Resurrecting the mysteries of big tau. *Trends Neurosci* **43**, 493–504 10.1016/j.tins.2020.04.007 (2020). PMC7999525 32434664

[11] Buchholz, S. & Zempel, H. The six brain-specific TAU isoforms and their role in Alzheimer’s disease and related neurodegenerative dementia syndromes. *Alzheimers Dement* **20**, 3606–3628 10.1002/alz.13784 (2024). PMC11095451 38556838

[12] Koppisetti, R. K. et al. Distribution of Big Tau Isoforms in the Human Central and Peripheral Nervous System. *Ann. Neurol.* **100**, 709–736 10.1002/ana.78300 (2026). PMC13587115 42473039

[13] Palmqvist, S. et al. Discriminative accuracy of plasma phospho-tau217 for Alzheimer disease vs other neurodegenerative disorders. *JAMA* **324**, 772–781 10.1001/jama.2020.12134 (2020). PMC7388060 32722745

[14] Nafash, M. N. et al. Plasma brain-derived tau: analytical and clinical validation of the first commercial immunoassay. *Sci. Rep.* **16**, 10124 10.1038/s41598-026-40271-6 (2026). PMC13021937 41723222

[15] Morgado, B. et al. Assessment of immunoprecipitation with subsequent immunoassays for the blood-based diagnosis of Alzheimer’s disease. *Eur. Arch. Psychiatry Clin. Neurosci.* **275**, 2215–2227 10.1007/s00406-023-01751-2 (2025). PMC12638416 38316685

[16] Morgado, B. et al. Evaluation of a fully automated assay for the measurement of plasma pTau217 and a composite score integrating the ratio Aβ1–42/1–40 as biomarkers of Alzheimer’s disease. *Eur. Arch. Psychiatry Clin. Neurosci*. **276**, 2251-2264 10.1007/s00406-025-02123-8 (2026). PMC13481745 41014347

[17] Bentivenga, G. M. et al. Clinical value of novel blood-based tau biomarkers in Creutzfeldt–Jakob disease. *Alzheimers Dement* **21**, e14422 10.1002/alz.14422 (2025). PMC11848332 39641397

[18] Agah, E. et al. CSF and blood levels of neurofilaments, T-tau, P-tau and Aβ42 in amyotrophic lateral sclerosis: a systematic review and meta-analysis. *J. Transl. Med.* **22**, 953 10.1186/s12967-024-05767-7 (2024). PMC11492992 39434139

[19] Barba, L., Otto, M. & Abu-Rumeileh, S. The underestimated relevance of Alzheimer’s disease copathology in amyotrophic lateral sclerosis. *J. Alzheimers Dis.* **95**, 1401–1404 10.3233/JAD-230900 (2023). 37807784

[20] Montoliu-Gaya, L. et al. Plasma tau biomarkers for biological staging of Alzheimer’s disease. *Nat. Aging* **5**, 2297–2308 10.1038/s43587-025-00951-w (2025). PMC12618263 40847200

[21] Roboscreen. BD-Tau antibody product information. https://www.roboscreen.com/index.php?eID=dumpFile&t=f&f=486&token=d83f35c261e96658ae96017c6485afa75380da51 (2024).

[22] Hanewinckel, R. et al. Prevalence of polyneuropathy in the general middle-aged and elderly population. *Neurology* **87**, 1892–1898 10.1212/WNL.0000000000003293 (2016). 27683845

[23] Richards, D., Morren, J. A. & Pioro, E. P. Time to diagnosis and factors affecting diagnostic delay in amyotrophic lateral sclerosis. *J. Neurol. Sci.* **417**, 117054 10.1016/j.jns.2020.117054 (2020). 32763509

[24] Takahashi, M. P. et al. Plasma neurofilament light chain and phosphorylated tau are elevated in myotonic dystrophy type 1. *J. Clin. Med.* **14**, 8197 10.3390/jcm14228197 (2025). PMC12653599 41303231

[25] Kaeser, S. A. et al. Blood phosphorylated tau elevation as a biomarker in immunoglobulin light chain and transthyretin amyloidosis. *Nat. Med*. 10.1038/s41591-026-04272-2 (2026). PMC13190286 41814005

[26] Jiang, Y. et al. Head-to-head comparison of brain-derived pTau217 and total pTau217 for brain amyloid and tau pathology classification. *Proc. Natl. Acad. Sci. USA.* **123**, e2536792123 10.1073/pnas.2536792123 (2026). PMC12974465 41770931

[27] Trelle, A. N. et al. Plasma proteomic signatures of preclinical Alzheimer’s disease in clinically unimpaired older adults. *Mol. Neurodegener.* **21**, 31 10.1186/s13024-026-00941-5 (2026). PMC13244826 42032756

[28] Gonzalez-Ortiz, F. et al. Association of serum brain-derived tau with clinical outcome and longitudinal change in patients with severe traumatic brain injury. *JAMA Netw. Open* **6**, e2321554 10.1001/jamanetworkopen.2023.21554 (2023). PMC10318474 37399012

[29] Gonzalez-Ortiz, F. et al. Plasma brain-derived tau correlates with cerebral infarct volume. *J. Intern. Med.* **297**, 173–185 10.1111/joim.20041 (2025). PMC11771704 39639627

[30] Khalil, M. et al. Neurofilaments as biomarkers in neurological disorders — towards clinical application. *Nat. Rev. Neurol.* **20**, 269–287 10.1038/s41582-024-00955-x (2024). 38609644

[31] Cousins, K. A. Q. et al. Elevated plasma phosphorylated tau 181 in amyotrophic lateral sclerosis. *Ann. Neurol.* **92**, 807–818 10.1002/ana.26462 (2022). PMC9588516 35877814

[32] Vacchiano, V. et al. Elevated plasma p-tau181 levels unrelated to Alzheimer’s disease pathology in amyotrophic lateral sclerosis. *J. Neurol. Neurosurg. Psychiatry* **94**, 428–435 10.1136/jnnp-2022-330709 (2023). 37012065

[33] Verde, F. et al. Phosphorylated tau in plasma could be a biomarker of lower motor neuron impairment in amyotrophic lateral sclerosis. *Neurol. Sci.* **44**, 3697–3702 10.1007/s10072-023-06916-4 (2023). 37369876

[34] Kawarabayashi, T. et al. Clinical validation of plasma p-217tau in neurological diseases. *Ann. Clin. Transl. Neurol.* 10.1002/acn3.70359 (2026). PMC13394761 41800832

[35] Janelidze, S. et al. Mitigating the associations of kidney dysfunction with blood biomarkers of Alzheimer disease by using phosphorylated tau to total tau ratios. *JAMA Neurol.* **80**, 516–522 10.1001/jamaneurol.2023.0199 (2023). PMC10061310 36987840

[36] Brooks, B. R. et al. El Escorial revisited: revised criteria for the diagnosis of amyotrophic lateral sclerosis. *Amyotroph. Lateral Scler. Other Motor Neuron Disord.* **1**, 293–299 10.1080/146608200300079536 (2000). 11464847

[37] Jack, C. R. Jr et al. NIA-AA Research Framework: toward a biological definition of Alzheimer’s disease. *Alzheimers Dement.* **14**, 535–562 10.1016/j.jalz.2018.02.018 (2018). PMC5958625 29653606

[38] Jack, C. R. Jr et al. Revised criteria for diagnosis and staging of Alzheimer’s disease. *Alzheimers Dement*. **20**, 5143–5169 10.1002/alz.13859 (2024). PMC11350039 38934362

[39] Swinnen, B. & Robberecht, W. The phenotypic variability of amyotrophic lateral sclerosis. *Nat. Rev. Neurol.* **10**, 661–670 10.1038/nrneurol.2014.184 (2014). 25311585

[40] Abu-Rumeileh, S. et al. Diagnostic-prognostic value and electrophysiological correlates of CSF biomarkers in amyotrophic lateral sclerosis. *J. Neurol.* **267**, 1699–1708 10.1007/s00415-020-09761-z (2020). 32100123

[41] Chiò, A. et al. Phenotypic heterogeneity of amyotrophic lateral sclerosis: a population-based study. *J. Neurol. Neurosurg. Psychiatry* **82**, 740–746 10.1136/jnnp.2010.235952 (2011). 21402743

[42] Roche, J. C. et al. A proposed staging system for amyotrophic lateral sclerosis. *Brain* **135**, 847–852 10.1093/brain/awr351 (2012). PMC3286327 22271664

[43] Nasreddine, Z. S. et al. The Montreal Cognitive Assessment, MoCA: a brief screening tool for mild cognitive impairment. *J. Am. Geriatr. Soc.* **53**, 695–699 10.1111/j.1532-5415.2005.53221.x (2005). 15817019

[44] Abrahams, S. et al. Screening for cognition and behaviour changes in ALS. *Amyotroph. Lateral Scler. Frontotemporal Degener.* **15**, 9–14 10.3109/21678421.2013.805784 (2014). 23781974

[45] Shahpasand-Kroner, H. et al. A two-step immunoassay for the simultaneous assessment of Aβ38, Aβ40 and Aβ42 in human blood plasma supports the Aβ42/Aβ40 ratio as a promising biomarker candidate of Alzheimer’s disease. *Alzheimers Res. Ther.* **10**, 121 10.1186/s13195-018-0448-x (2018). PMC6286509 30526652

[46] Dugger, B. N. et al. The presence of select tau species in human peripheral tissues and their relation to Alzheimer’s disease. *J. Alzheimers Dis.* **51**, 345–356 10.3233/JAD-150859 (2016). PMC6074044 26890756

[47] Hamsafar, Y. et al. Biochemical analyses of tau and other neuronal markers in the submandibular gland and frontal cortex across stages of Alzheimer disease. *Neurosci. Lett.* **810**, 137330 10.1016/j.neulet.2023.137330 (2023). PMC11006283 37330193

[48] Ellis, M. J. et al. Identification of high-performing antibodies for the reliable detection of tau proteoforms by Western blotting and immunohistochemistry. *Acta Neuropathol.* **147**, 87 10.1007/s00401-024-02729-7 (2024). PMC11102361 38761203

[49] Kavanagh, T. et al. The interactome of tau phosphorylated at T217 in Alzheimer’s disease human brain tissue. *Acta Neuropathol.* **149**, 44 10.1007/s00401-025-02881-8 (2025). PMC12049313 40317322

[50] MacLean, B. et al. Skyline: an open source document editor for creating and analyzing targeted proteomics experiments. *Bioinformatics* **26**, 966–968 10.1093/bioinformatics/btq054 (2010). PMC2844992 20147306

[51] Deutsch, E. W. et al. The ProteomeXchange consortium in 2026: making proteomics data FAIR. *Nucleic Acids Res* **54**, D459–D469 10.1093/nar/gkaf1146 (2026). PMC12807779 41206473

[52] Perez-Riverol, Y. et al. The PRIDE database at 20 years: 2025 update. *Nucleic Acids Res.* **53**, D543–D553 10.1093/nar/gkae1011 (2025). PMC11701690 39494541

[53] Sievers, F. et al. Fast, scalable generation of high-quality protein multiple sequence alignments using Clustal Omega. *Mol. Syst. Biol.* **7**, 539 10.1038/msb.2011.75 (2011). PMC3261699 21988835

