# Supplementary material for: Tau isoform mapping across tissues and biofluids reveals a signature distinguishing Alzheimer’s disease from amyotrophic lateral sclerosis
Source: Nat Commun. 2026 Oct 22;17:10745. doi: 10.1038/s41467-026-78390-3 (PMC13691104; doi:10.1038/s41467-026-78390-3)
Supplement: Supplementary file 1 [file 41467_2026_78390_MOESM1_ESM.pdf]

SUPPLEMENTARY INFORMATION

SUPPLEMENTARY RESULTS

One-step approach: Serum and CSF tau biomarkers in the diagnostic groups using direct Simoa immunoassays

Serum brain-derived (BD)-tau showed weak-to-moderate associations with age in the whole cohort (n = 337, Spearman's r = 0.449, p < 0.001), in the amyotrophic lateral sclerosis (ALS) group (n = 139, r = 0.291, p = 0.001), and in disease controls (n = 75, r = 0.394, p < 0.001), but not in the Alzheimer's disease (AD) (n=101) or healthy control groups (n = 22). In the combined control group (disease controls and healthy controls; n = 97), age correlated weakly with serum BD-tau (r = 0.259, p < 0.001). Cerebrospinal fluid (CSF) BD-tau was weakly to moderately associated with age in the whole cohort (n = 143, Spearman's r = 0.570, p < 0.001) and in the ALS group (n = 61, r = 0.432, p = 0.001), but not in the AD (n = 51) or disease control groups (n = 31). CSF total tau (t-tau) showed weak-to-moderate associations with age in the whole cohort (n = 292, Spearman's r = 0.556, p < 0.001), in the ALS group (n = 116, r = 0.455, p < 0.001), and in disease controls (n = 75, r = 0.261, p = 0.024), but not in the AD group. Serum phosphorylated tau 217 (p-tau217) correlated weakly to moderately with age in the whole cohort (n = 337, Spearman's r = 0.464, p < 0.001) and in disease controls (n = 75, r = 0.389, p = 0.001), but not in the AD and ALS groups.

After including healthy controls in the analysis, higher serum BD-tau levels were confirmed in ALS (n = 139) and AD (n = 101) compared with disease controls (n = 75; both p < 0.001) and healthy controls (n = 22; both p < 0.001), with no statistically significant difference between healthy controls and disease controls.

When measured with the one-step approach, serum and CSF BD-tau showed a moderate correlation in the whole cohort (n = 143, Spearman's r = 0.432, p < 0.001), with no association observed in the ALS, AD, or disease control groups. Serum BD-tau and CSF t-tau were also moderately associated in the whole cohort (n = 292, Spearman's r = 0.477, p < 0.001), but not in the ALS, AD, or disease control groups. Strong correlations

were observed between CSF BD-tau and CSF t-tau in the whole cohort (n = 143, r = 0.963, $p < 0.001$), ALS (n = 61, r = 0.902, $p < 0.001$), AD (n = 51, r = 0.879, $p < 0.001$), and disease control groups (n = 31, r = 0.921, $p < 0.001$).

After adjustment for centre, CSF t-tau was moderately associated with serum BD-tau in the whole cohort (n = 292, β = 0.453, $p < 0.001$), but no such association was observed in the ALS, AD, or disease control groups. Even after adjustment for centre, CSF BD-tau and CSF t-tau showed strong associations in the whole cohort (n = 143, β = 0.962, $p < 0.001$), as well as in ALS (n = 61, β = 0.881, $p < 0.001$), AD (n = 51, β = 0.911, $p < 0.001$), and disease control groups (n = 31, β = 0.947, $p < 0.001$).

Serum BD-tau and/or CSF BD-tau were also variably associated with serum serum phosphorylated tau 181 (p-tau181), serum p-tau217, serum t-tau, and serum neurofilament light chain protein (NfL) across diagnostic groups. In both ALS and AD groups, serum BD-tau correlated with p-tau181 (ALS: n = 139, r = 0.568, $p < 0.001$; AD: n = 101, r = 0.571, $p < 0.001$), serum p-tau217 (ALS: n = 139, r = 0.595, $p < 0.001$; AD: n = 101, r = 0.599, $p < 0.001$), serum t-tau (ALS: n = 139, r = 0.210, p = 0.014; AD: n = 101, r = 0.280, p = 0.005), and serum NfL (ALS: n = 139, r = 0.390, $p < 0.001$; AD: n = 101, r = 0.435, $p < 0.001$). In the disease control group (n = 75), serum BD-tau was associated with serum p-tau181 (r = 0.481, $p < 0.001$), serum p-tau217 (r = 0.510, $p < 0.001$), serum t-tau (r = 0.354, p = 0.002), and serum NfL (r = 0.529, $p < 0.001$). In ALS (n = 61), CSF BD-tau correlated with serum NfL (r = 0.415, p = 0.001). In the AD and disease control groups, CSF BD-tau was not associated with any serum biomarker.

In the whole sample, serum BD-tau (one-step immunoassay) yielded good diagnostic accuracy in discriminating AD (n = 101) from disease controls (n = 75; area under the curve [AUC] ± standard deviation, 0.886 ± 0.026), but only poor-to-moderate performance in distinguishing ALS (n = 139) from AD (n = 101; AUC, 0.676 ± 0.034) or disease controls (n = 75; AUC, 0.743 ± 0.034). CSF BD-tau showed excellent performance in distinguishing AD (n = 51) from disease controls (n = 31; AUC, 0.982 ± 0.012) or ALS (n = 61; AUC, 0.976 ± 0.012), but lower performance in distinguishing ALS (n = 61) from disease controls (n = 31; AUC, 0.607 ± 0.065).

Two-step approach: serum BD-tau and BD-p-tau217 measurements after pre-analytical semi-automated BD-tau immunoprecipitation (BD-Tau-IP)

Serum BD-tau after BD-tau immunoprecipitation (BD-Tau-IP) showed moderate associations with age in the whole cohort (n = 149, Spearman's r = 0.414, p < 0.001), in the ALS group (n = 50, r = 0.397, p = 0.005) but not in the AD (n = 60) or disease control groups (n = 39). Serum brain-derived phosphorylated tau 217 (BD-p-tau217) correlated moderately with age in the whole cohort (n = 149, Spearman's r = 0.535, p < 0.001), in the ALS group (n = 50, r = 0.461, p = 0.001), but not in the AD (n = 60) or disease control groups (n = 39). Sex did not influence either BD-tau or BD-p-tau217.

Strong correlations were observed between serum concentrations of BD-tau and p-tau217 measured with the one-step approach and the corresponding values with the two-step approach (n = 149; r = 0.801, p < 0.001 and r = 0.727, p < 0.001, respectively). These correlations were stronger in the AD group (n = 60; BD-tau, r = 0.794, p < 0.001; p-tau217, r = 0.774, p < 0.001) than in the control (n = 39; BD-tau, r = 0.776, p < 0.001; p-tau217, r = 0.634, p < 0.001) and ALS groups (n = 50; BD-tau, r = 0.702, p < 0.001; p-tau217, r = 0.549, p < 0.001).

First manual BD-Tau-IP run and subsequent serum BD-tau and p-tau217 measurements

After manual BD-Tau-IP (n = 64), patients with AD (n = 22) showed higher serum BD-tau and BD-p-tau217 levels compared with ALS patients (n = 21; p = 0.004 and p < 0.001, respectively) and disease controls (n = 21; p < 0.001 and p < 0.001, respectively), with no differences between the latter two groups (Supplementary Table 7 and Supplementary Figure 1). These findings were confirmed after age adjustment (AD, n = 22 vs disease controls, n = 21: BD-tau, β = 0.380, p = 0.019; BD-p-tau217, β = 0.852, p < 0.001; ALS, n = 21 vs disease controls, n = 21: BD-tau, not significant; BD-p-tau217, not significant; AD, n = 22 vs ALS, n = 21: BD-tau, β = 0.439, p = 0.009; BD-p-tau217, β = 0.842, p < 0.001). In the same subgroups, we found similar distributions of serum p-tau217 and BD-tau measured without pre-analytical BD-Tau-IP to those observed in the whole cohort (Supplementary Table 7 and Supplementary Figure 1).

Strong correlations were observed between serum concentrations of BD-tau and p-tau217 measured without pre-treatment and the corresponding values in BD-Tau-IP eluates (n = 64; BD-tau, r = 0.835, p < 0.001; p-tau217, r = 0.836, p < 0.001). These correlations were stronger in the AD group (n = 22; BD-tau, r = 0.912, p

< 0.001; p-tau217, $r = 0.929$, $p < 0.001$) than in the control group ($n = 21$; BD-tau, $r = 0.771$, $p < 0.001$; p-tau217, $r = 0.523$, $p = 0.015$) and the ALS group ($n = 21$; BD-tau, $r = 0.592$, $p = 0.005$; p-tau217, $r = 0.593$, $p = 0.005$).

We calculated the ratio of serum BD-tau to BD-p-tau217 concentrations measured after BD-Tau-IP. Participants with AD ($n = 22$) showed significantly lower ratio values compared with both ALS subjects ($n = 21$; $p < 0.001$) and disease controls ($n = 21$; $p < 0.001$) (Supplementary Table 7), even after adjustment for age (AD, $n = 22$ vs ALS, $n = 21$: $\beta = -0.745$, $p < 0.001$; AD, $n = 22$ vs disease controls, $n = 21$: $\beta = -0.449$, $p = 0.007$).

Pre-analytical BD-Tau-IP did not significantly modify the AUC of BD-tau for discriminating among AD ($n = 22$), ALS ($n = 21$), and disease controls ($n = 21$), compared with the approach without BD-Tau-IP. However, serum BD-tau after BD-Tau-IP showed a numerically lower AUC in the comparison ALS vs. disease controls, although the difference was not statistically significant (Supplementary Table 8). Conversely, BD-Tau-IP significantly increased the AUC of serum p-tau217 (i.e. BD-p-tau217) for discriminating AD from ALS and significantly reduced the AUC of the same biomarker when comparing ALS with disease controls. However, BD-Tau-IP did not significantly improve the performance of p-tau217 (i.e. BD-p-tau217) in the comparison AD vs. disease controls (Supplementary Table 8).

The BD-tau/BD-p-tau217 ratio (BD-Tau-IP + Simoa) did not perform better than either single blood biomarker (BD-tau or p-tau217) in differentiating between AD and disease controls or between AD and ALS (Supplementary Table 8).

Second manual BD-Tau-IP run and subsequent serum p-tau217 measurements

After manual BD-Tau-IP ($n = 98$), patients with AD ($n = 39$) showed higher BD-p-tau217 levels compared with ALS patients ($n = 45$) and disease controls ($n = 14$; both $p < 0.001$), with no differences between the latter two groups (Supplementary Table 9 and Supplementary Figure 2). These findings were confirmed after age adjustment (AD, $n = 39$ vs disease controls, $n = 14$: $\beta = 0.652$, $p < 0.001$; ALS, $n = 45$ vs disease controls, $n = 14$: not significant; AD, $n = 39$ vs ALS, $n = 45$: $\beta = 0.621$, $p < 0.001$). In the same subgroups, there were similar distributions of serum p-tau217 measured without pre-analytical BD-Tau-IP to those observed in the whole cohort (Supplementary Table 9 and Supplementary Figure 2).

A strong correlation was observed between serum p-tau217 concentrations measured without pre-treatment and the corresponding values in BD-Tau-IP eluates (n = 98, r = 0.610, p < 0.001). This association was replicated only in the AD group (n = 39, r = 0.781, p < 0.001), but not in ALS cases or disease controls.

BD-Tau-IP significantly increased the AUC of serum p-tau217 (i.e. BD-p-tau217) for discriminating AD (n = 39) from ALS (n = 45) and significantly reduced the AUC of the same biomarker when comparing ALS with disease controls. However, BD-Tau-IP did not significantly improve the performance of p-tau217 (i.e. BD-p-tau217) in discriminating between AD and disease controls (Supplementary Table 10).

Analytical reproducibility of the two-step approach

The analytical reproducibility of the BD-Tau-IP two-step approach was assessed at two levels: the pre-analytical BD-Tau-IP procedure and the downstream Simoa measurement platform. The analytical reproducibility of the manual and semi-automated BD-Tau-IP protocols was evaluated using serum pool samples. For the manual BD-Tau-IP, intra-assay coefficient of variation (CV) was 7.2 % and inter-assay CV was 9.5 %. For the semi-automated BD-Tau-IP, intra-assay CV was 6.8 % and inter-assay CV was 8.7 %. The analytical reproducibility of the Simoa assays was also assessed. For both BD-tau and p-tau217, intra-assay CV was <10% and inter-assay CV was <15%, indicating robust analytical performance of the measurement platform.

Inter-method reproducibility between the manual and semi-automated BD-Tau-IP procedures was tested by Spearman correlation and Bland-Altman analysis in the 98 subjects who underwent both procedures. Strong rank-order correlation between the two procedures was observed (Spearman's r = 0.877, p < 0.001), supporting the consistency of relative biomarker distributions across both BD-Tau-IP approaches. Bland-Altman analysis revealed an acceptable inter-method agreement with a mean difference (manual minus semi-automated) of +0.775 relative units (standard deviation, SD 0.889; 95% limits of agreement, LoA: -0.967 to +2.518) and with 92.9% of measurements falling within the limits of agreement. Agreement was better in ALS (mean difference: +0.335, LoA: -0.407 to +1.077) and controls (mean difference: +0.265, LoA: -0.038 to +0.567) than in AD subjects (mean difference: +1.467, LoA: -0.520 to +3.453), likely attributable to the higher absolute BD-p-tau217 values in the AD group, which amplify absolute differences between methods even when relative agreement is maintained (a known feature of Bland-Altman analysis in datasets with wide dynamic ranges).

Taken together, these findings indicate acceptable inter-method reproducibility, with a systematic tendency for the manual IP to yield higher values compared with the semi-automated procedure, consistent with known procedural differences in starting volumes, elution volume and dilution factors between the two protocols. Importantly, despite the higher absolute differences observed in AD subjects, the diagnostic separation between AD and the other groups (ALS and controls) was robustly maintained with both manual and semi-automated BD-Tau-IP procedures, confirming that inter-method variability does not compromise the diagnostic performance of the two-step approach.

Identification of exon 4a-L and exon 4a-S isoforms in muscle tissue by untargeted mass spectrometry

Two pan-tau-IP muscle samples were additionally analysed by untargeted, data-dependent mass spectrometry to investigate the expression of the two exon 4a splice variants, namely big tau with exon 4a-long (4a-L) and big tau with exon 4a-short (4a-S). Three peptides were identified, indicating the co-expression of both isoforms: one spanning the exon 4-to-exon 4a-long junction (specific for exon 4a-L), one the exon 4a-long-to-exon 4a junction (specific for exon 4a-L), and one the exon 4-to-exon 4a junction (specific for exon 4a-S). These results confirm the presence of both exon 4a-L and exon 4a-S isoforms in muscle tissue (Figure 3 and Supplementary Figure 3).

SUPPLEMENTARY METHODS

Case selection for blood and CSF biomarker analyses

The Halle cohort included 58 ALS patients, 59 AD patients (45 dementia and 14 mild cognitive impairment), and 30 disease controls; the Milan cohort included 65 ALS patients, 17 AD patients (13 dementia and 4 mild cognitive impairment), and 8 disease controls; the Mannheim 1 cohort included 16 ALS patients and 12 disease controls; and the Mannheim 2 cohort included 25 AD patients (19 dementia and 6 mild cognitive impairment) and 25 controls.

Inclusion criteria for disease controls were: (1) individuals lacking any clinical evidence of neurodegenerative disease; (2) absence of structural brain or spinal cord disease on magnetic resonance imaging; (3) absence of CSF evidence of CNS inflammatory disease, including normal white blood cell count and absence of oligoclonal bands; (4) normal AD CSF core biomarkers according to in-house reference values; and (5) normal serum NfL levels according to in-house reference values[1] (as a sensitive and disease-agnostic filter against subclinical neuronal injury, regardless of the underlying diagnosis).

The disease control group (n = 75) comprised individuals with: primary psychiatric disorders, including a subset with mild cognitive symptoms (n = 29); subjective cognitive impairment with normal cognitive testing (n = 8); first unprovoked seizure or epilepsy without structural brain disease (n = 5); primary headache (n = 4); idiopathic non-inflammatory polyneuropathy (n = 6); non-neurodegenerative movement disorders (e.g. functional movement disorder, hemifacial spasm, focal dystonia) (n = 8); Horner syndrome (n = 2); cranial or peripheral nerve palsies (n = 3); and other minor neurological diagnoses (n = 10) not associated with neuronal injury.

Healthy controls were individuals younger than 50 years with no history of neurological disease, normal Mini-Mental State Examination scores, and normal serum NfL levels according to in-house cut-off values[1]. CSF AD biomarker assessment was not performed in these individuals; however, given their age, the presence of incidental AD co-pathology was considered unlikely[2].

ALS clinical phenotypes included classic, bulbar, upper motor neuron–predominant (including primary lateral sclerosis), and lower motor neuron–predominant forms (including flail arm syndrome, flail leg syndrome, and progressive muscular atrophy)[3,4]. Disease progression rate at blood sampling was calculated as follows: (48 −

ALSFRS-R score at the time of sampling) divided by the time between symptom onset and blood sampling (months)[4].

Preparation of functionalized magnetic beads and pre-analytical BD-Tau-immunoprecipitation

Dynabeads™ M-270 Epoxy Magnetic Beads (Thermo Fisher, USA) were loaded with 2B8 antibody according to the protocol of the manufacturer and subsequently stored in storage buffer (20 mM PBS, 0.05 % Tween 20, 0.1% BSA, pH 7.4 containing 0.02% Na-Azide) at 2-10°C. All BD-Tau-IP experiments in the present study were performed using the same antibody clone 2B8 (catalogue number 847-010200760, Roboscreen GmbH, Leipzig, Germany) and bead preparation batch 0325-01, thus excluding batch-to-batch variability as a source of analytical variability in the current dataset. The amount of antibody used per aliquot of patient sample was 10 µg for the first manual IP and for the semi-automated IP, and 5 µg for the second manual IP.

The first run of BD-Tau IP from serum was performed manually. In summary, aliquots of serum stored at –80°C in Matrix 0.5 mL tubes were thawed at room temperature (RT), mixed vigorously for 5-10 s and centrifuged for 10 min at 10,000 × g at RT in a fixed angle rotor for removal of insoluble material. 50 µL of functionalized magnetic beads were pipetted into low bind 1.5 mL tubes (Eppendorf, Germany) and washed once with 1 mL Wash buffer (20 mM PBS containing 0.1% BSA and 0.1% Proclin 300, pH 7.4). We started from two aliquots of 500 µL of serum per patient. Each serum aliquot was mixed by vortexing with 125 µL of 5 × IP-buffer concentrate (250 mM HEPES/NaOH, pH 7.4, 750 mM NaCl, 2.5% Igepal CA630, 1.25% sodium deoxycholate; 0.25% SDS, Complete Mini Protease inhibitor cocktail of Roche, Switzerland). BD-Tau-IP started with a binding step carried out for 1 hour at room-temperature with periodic mixing using overhead rotation by HULA-Mixer (Thermo, USA) at 100 rpm. Subsequently the beads were washed 3 × for 5 min with 1 mL of Wash buffer followed by 1 × 5 min washing using 1 mL elution buffer (20 mM PBS containing 0.05% Tween 20, pH 7.4). The bound BD-tau proteins were eluted from the magnetic beads by adding 50 µL of elution buffer PBS-T and heating the tubes in a Thermomixer 1.5 mL (Eppendorf) for 10 min at 99 °C and 300 rpm mixing. After that, the tubes were cooled down in a cooling rack followed by collection of approximately two 50 µL IP-eluates (per patient). The two IP eluates of each patient sample were manually transferred into 2 mL screw cap micro tubes (Sarstedt, Germany) mixed by vortexing and stored at −80 °C until the BD-tau and p-

tau217 measurements on the Simoa platform were performed. In the manual protocol, all pipetting, washing, and bead handling steps were performed manually by the operator.

The second manual BD-Tau-IP from serum was performed using residual serum from the semi-automated IP run, for which 200 µL of serum were still available. In summary, aliquots of serum stored at –80 °C in Matrix 0.5 mL tubes were thawed at RT, mixed vigorously for 5-10 s and centrifuged for 10 min at 10,000 × g at RT in a fixed angle rotor for removal of insoluble material. 25 µL of functionalized magnetic beads were pipetted into low bind 1.5 mL tubes and washed once with 1 mL Wash buffer. After that, 200 µL of the serum for each patient was added and mixed by vortexing followed by addition of 50 µL of 5 × IP-buffer concentrate. BD-Tau-IP started with a binding step carried out at room-temperature for 1 hour with periodic mixing using overhead rotation at 100 rpm. Subsequently the beads were washed 3 × for 5 min with 1 mL of wash buffer followed by 1 × 5 min washing using 1 mL elution buffer. The bound BD-tau proteins were eluted from the magnetic beads by adding 25 µL of elution buffer (PBS-T) and heating the tubes in a Thermomixer for 10 min at 99 °C and 300 rpm mixing. After that, the tubes were cooled down in a cooling rack followed by collection of approximately 30 µL of eluate. Eluates were transferred individually into 2 mL screw cap micro tubes (Sarstedt, Germany) without pooling and stored at −80°C until p-tau217 measurements on the Simoa assays were performed.

The semi-automated pre-analytical BD-Tau-IP from serum was performed on a CyBio FeliX liquid handling Instrument (Analytik Jena, Jena, Germany). In summary, aliquots of serum stored at –80 °C in Matrix 0.5 mL tubes were thawed at RT, mixed vigorously for 5-10 s and centrifuged for 10 min at 10,000 × g at RT in a fixed angle rotor for removal of insoluble material. We started from two aliquots of 400 µL of serum per patient. Each serum aliquot was mixed by vortexing in a 96-deepwell sample plate (Sarstedt, Nümbrecht, Germany) with 100 µL of 5 × IP-buffer concentrate. Subsequently 50 µL of functionalized magnetic beads were added automatically by means of the CyBio FeliX liquid handling Instrument. The semi-automated BD-Tau-IP started with a binding step carried out at room-temperature for 1 hour with periodic mixing. Subsequently the beads were washed 3 × for 5 min with 1 mL of Wash buffer and 1 × for 3 min with 1 mL of elution buffer. The bound BD-Tau proteins were eluted from the magnetic beads by adding 2 × 100 µL of elution buffer and heating the 96 deepwell round bottom plate (Sarstedt, Germany) without a lid on a BioShake 3000-T elm (QInstruments,

Germany) for 5 min at 99 °C and 1100 rpm. Per patient, approximately 2 × 75 µL of bead-free BD-Tau eluate was obtained. The two IP eluates of each patient sample were manually transferred into 2 mL screw cap micro tubes (Sarstedt, Germany) mixed by vortexing and stored at −80 °C until the BD-tau and p-tau217 measurements on the Simoa platform were performed.

In the semi-automated protocol, these steps were performed using a CyBio FeliX liquid handling instrument (Analytik Jena, Germany), with only the transfer of the final eluates into storage tubes performed manually. In all protocols, eluates were measured after a single freeze-thaw cycle.

BD-Tau-IP eluates were used to measure the serum levels of BD-tau and/or BD-p-tau217. For BD-tau measurements, eluates were diluted 1:4 according to the manufacturer's instructions (35 µL eluate + 105 µL diluent). Following the first manual and the semi-automated BD-Tau-IP, eluates were diluted 1:3 for BD-p-tau217 quantification, as recommended by the manufacturer (45 µL eluate + 90 µL diluent). Due to limited eluate volume after the second manual BD-Tau-IP, BD-p-tau217 was measured using a 1:5 dilution (26 µL eluate + 104 µL diluent).

Other serum and CSF biomarkers

Serum p-tau181, serum p-tau217, and serum total tau (t-tau) were measured using the Simoa platform, whereas serum NfL was measured using the Ella microfluidic system (Bio-Techne, Minneapolis, MN, USA) at the Department of Neurology in Halle[1]. CSF AD core biomarkers (A: Aβ42/Aβ40; T: p-tau181; N: t-tau) from the Halle and Mannheim 1 cohorts were measured in Halle (Saale), whereas CSF samples from the remaining cohorts were analysed at the respective centres[1]. All CSF AD biomarkers were measured across centres using an automated chemiluminescent enzyme immunoassay on the Lumipulse platform (Fujirebio, Ghent, Belgium)[1].

Pathological values for AD core biomarkers (A+, T+, and N+) were defined according to centre-specific in-house validated cut-off values (Halle and Mannheim 1: Aβ42/Aβ40 < 0.069, p-tau181 > 56.5 pg/mL, t-tau > 400 pg/mL; Milan: Aβ42/Aβ40 < 0.069, p-tau181 > 56.5 pg/mL, t-tau > 404 pg/mL; Mannheim 2: Aβ42/Aβ40 < 0.05, p-tau181 > 61.0 pg/mL, t-tau > 450 pg/mL)[1].

Brain, spinal cord and muscle sample collection

Individuals were diagnosed as AD when they were clinically diagnosed with dementia (based on the CDR score) and had at least an intermediate degree of AD neuropathologic changes according to National Institute on Aging–Alzheimer's Association (NIA-AA) criteria[5,6]. Diagnosis of ALS and/or frontotemporal lobar degeneration (FTLD) was established according to accepted clinical criteria[7-9]. The post-mortem diagnosis of ALS and FTLD was pathologically confirmed by assessment of the pTDP-43 pathology. Disease controls consisted of individuals without evidence of neurodegenerative disease. ALS cases and disease controls displayed none or low AD-related neuropathologic change, not sufficient to meet criteria for AD co-pathology[6].

Pan-tau and p-tau217 immunoprecipitation

The semi-automated pre-analytical pan-tau and p-tau217 immunoprecipitations (IP) from protein extracts were performed on a CyBio FeliX liquid handling instrument (Analytik Jena, Jena, Germany). In summary, aliquots of protein extracts stored at –80 °C in 1.5 mL tubes were thawed at RT, mixed vigorously for 5–10 s and centrifuged for 10 min at 10,000 × g at RT in a fixed angle rotor for removal of insoluble material.

The amount of total protein used as starting material for pan-tau-IP was adjusted according to the expected tau concentration in each tissue: 50 µg for frontal cortex, 240 µg for spinal cord and 1,000 µg for skeletal muscle. For pan-tau-IP, the volume of protein extract required to reach the target protein amount was calculated from the individual sample concentration and adjusted to a final volume of 400 µL for all samples, irrespective of tissue. For the p-tau217-IP on muscle samples, the volume of protein extract used was adjusted based on the individual sample concentration to obtain approximately 1,000 µg of total protein per sample.

Each protein extract aliquot was mixed by vortexing in a 96-deepwell sample plate (Sarstedt, Nümbrecht, Germany) with 100 µL of 5 × IP-buffer concentrate. For pan-tau-IP, functionalized magnetic beads with pan-tau antibody 7E5 against the amino acid sequence RGAAPPGQKGQA of 2N4R tau (catalogue number 847-0102006301, Roboscreen GmbH, Leipzig, Germany) were used. For p-tau217-IP, functionalized magnetic beads with a Phospho-Tau (Thr217) polyclonal antibody (catalogue number 44-744, Thermo Fisher Scientific, Invitrogen, USA) were used. Subsequently, 50 µL of functionalized magnetic beads were used for each IP per aliquot. Functionalization of magnetic beads was performed according to the protocol described in the methods and supplementary method section of the manuscript. The amount of antibody used per aliquot was 10 µg for

both the pan-tau and the p-tau217-IP. Functionalized magnetic beads were added automatically by means of the CyBio FeliX liquid handling instrument. The semi-automated IP started with a binding step carried out at room temperature for 1 hour with periodic mixing. Subsequently, the beads were washed 3 × for 5 min with 1 mL of wash buffer and 1 × for 3 min with 1 mL of elution buffer. The bound tau proteins were eluted from the magnetic beads by adding 100 µL of elution buffer and heating the 96-deepwell round bottom plate (Sarstedt, Germany) without a lid on a BioShake 3000-T elm (QInstruments, Germany) for 15 min at 99 °C and 1,100 rpm. Per sample, approximately 75 µL of pan-tau or p-tau217 eluate was obtained. The IP eluates were manually transferred into 2 mL screw cap micro tubes (Sarstedt, Germany) and stored at −80 °C until mass spectrometry analysis was performed.

Targeted mass spectrometry analysis of tau species after pan-tau-IP and p-tau217-IP

Eluates from pan-tau-IP and p-tau217-IP were subjected to a two-step proteolytic digestion using Asp-N and Lys-C in a paramagnetic bead–based workflow. The eluates were reduced with 50 mM TCEP (Sigma-Aldrich, USA) for 30min at 55°C and carbamidomethylated with 100 mM CAA (Merck, Germany) for 30min at room temperature. Proteins were precipitated on magnetic carboxylate-modified particles (Sigma-Aldrich, USA) by adding twice the volume of acetonitrile, then washed twice with 80% ethanol in water. Bead-bound proteins were digested with Asp-N (Promega, USA) for 2h at 37°C and consecutively with Lys-C (Promega, USA) for 8h at 37°C. The peptides were subsequently collected, dried in a vacuum concentrator, and reconstituted in 0.1% FA for liquid chromatography-tandem mass spectrometry (LC-MS/MS) analysis. The digested samples were analysed by targeted mass spectrometry using parallel reaction monitoring, focusing on the following peptide sequences: "DEAAGHVTQARMVSK", which is specific to BD-tau isoforms, "VSTEIPASEPDGPSVGRAK" and "DTEGGRHAPELLK", which are specific to big tau isoforms containing exon 4a, "IATPRGAAPPGQK" and "LTFRENAK", which are common to all tau isoforms (t-tau). Peptide-specific settings are listed in Supplementary Table 16.

The LC-MS/MS analysis of the samples was conducted blinded to diagnoses and allocation on an EASY-nLC 1200 system (Thermo Fisher Scientific, USA), which was coupled to an Orbitrap Fusion Lumos Tribrid mass spectrometer (Thermo Fisher Scientific, USA) via a FAIMS Pro DUO interface (Thermo Fisher Scientific,

USA). The peptides were trapped and desalted on a C18 pre-column (Acclaim PepMap 100, 75 µm × 2 cm, C18) and subsequently separated on a C18 analytical column (Aurora Ultimate TS, 75 µm × 25 cm, C18).

A bipartite linear 28 min gradient with 300 nL/min flow starting from 4% eluent B (0.1% FA in 80% ACN) in eluent A (0.1% FA in water) to 45% eluent B via 20% eluent B after 17 min was used for pan-tau-IP samples. After each sample, the column was flushed to 98% eluent B and reconstituted to starting conditions. PRMs for all targeted peptides were acquired in cycles, followed by a survey scan after each complete set. For PRMs, the following parameters were set: Isolation Mode = Quadrupole, Isolation Window = 0.7 m/z, HCD activation with 30% normalized collision energy, Detector Type = Orbitrap, Resolution = 30k, AGC = 200%, maximum injection time = 54 ms. For MS1 scans the following parameters were set: m/z range 375-1000, maximum injection time = 118 ms, normalized AGC target = 200%, R = 60 000, FAIMS CV = -65V.

A tripartite linear 53 min gradient with 300 nL/min flow starting from 4% eluent B (0.1% FA in 80% ACN) in eluent A (0.1% FA in water) to 45% eluent B via 24 % eluent B after 33.5 min and 34 % eluent B after 45.5 min was used for p-tau217-IP samples. After each sample, the column was flushed to 98% eluent B and reconstituted to starting conditions. PRMs for all the targeted peptides were acquired in cycles, followed by a survey scan after each complete set. For PRMs, the following parameters were set: Isolation Mode = Quadrupole, Isolation Window = 0.7 m/z, HCD activation with 30% normalized collision energy, Detector Type = Orbitrap, Resolution = 60k, AGC = 200%, maximum injection time = 118 ms. For MS1 scans the following parameters were set: m/z range 375-1000, maximum injection time = 118 ms, normalized AGC target = 200%, R = 60 000, FAIMS CV = -65V.

Raw LC–MS/MS data were processed using Skyline (v25.1.0.237) software.

Untargeted mass spectrometry analysis of tau species captured by pan-tau-IP

The sample preparation for untargeted mass spectrometry analysis followed the same protocol as the sample preparation for targeted MS described above. The two pan-tau-IP muscle samples were analysed on an EASY-nLC 1200 system (Thermo Fisher Scientific, USA), which was coupled to an Orbitrap Fusion Lumos Tribrid mass spectrometer (Thermo Fisher Scientific, USA) via a FAIMS Pro DUO interface (Thermo Fisher

Scientific, USA). The peptides were trapped and desalted on a C18 pre-column (Acclaim PepMap 100, 75 µm x 2 cm, C18) and subsequently separated on a C18 analytical column (Aurora Ultimate TS, 75 µm x 25 cm, C18).

A tripartite linear 105 min gradient with 300 nL/min flow starting from 6 % eluent B (0.1% FA in 80 % ACN) in eluent A (0.1 % FA in water) to 45% eluent B via 24% eluent B after 66 min and 34% eluent B after 90 min was used. After each sample, the column was flushed to 98% eluent B and reconstituted to starting conditions. Mass spectra were acquired in a data-dependent manner (DDA). For MS1 scans the following parameters were set: m/z range 375-1500, maximum injection time = 100 ms, normalized AGC target = 200%, R = 120 000. The most abundant ions were selected for MS2 acquisition with a fixed cycle time of 1s using the following parameters: isolation window of 1.4 m/z, maximum injection time 75 ms, normalized AGC target = 100%, normalized collision energy (NCE) = 30, R = 15 000. Fragmented ions were dynamically excluded for 60 s. The FAIMS-CV was set to alternating cycles of -50V and -65V. The LC–MS/MS raw data were examined by MaxQuant (Version 2.6.1.0, Max Planck Institute of Biochemistry, Martinsried, Germany)[10]. Database search was performed against the UniProt Homo Sapiens Reviewed RefSet with isoform information (02/2025), the sequence of big tau with exon 4a-long described by Koppisetti et al.[11], and a list of common contaminants provided by MaxQuant (07/2019, 245 entries)[12]. Search parameters were set as follows: Maximum missed cleavages = 2, minimal peptide length = 7 amino acids, first search peptide tolerance = 20 ppm, main search peptide tolerance = 4.5 ppm, FTMS MS/MS match tolerance = 20 ppm. Carbamidomethylation of cysteine was set as a fixed modification, protein N-terminal acetylation, and oxidation of methionine were set as variable modifications. Peptides, proteins, and sites were filtered by a target-decoy approach to an FDR ≤ 0.01 using a reversed decoy database.

Supplementary Table 1. Demographic characteristics and serum biomarker distribution in healthy controls (n=22).

**Age (years)** mean±SD<br>min-max	35.3±7.7<br>22 – 48
**Female (%)**	63.6
**Serum BD-tau (pg/mL)**<br>Median (IQR)	6.72 (5.99-7.78)

BD-tau brain-derived tau protein, *IQR* interquartile range, *max* maximal value, *min* minimal value, *SD* standard deviation

Supplementary Table 2. Demographic characteristics and biomarker distribution in the diagnostic groups in the Halle cohort.

Halle				
	**ALS**	**AD**	**Disease controls**	**p-value**
**N with serum samples**	58	59	30	
**Age (years)** mean±SD	63.9±10.8	75.7±7.7	59.9±14.4	<0.001
**Female (%)**	27.6	59.3	56.7	<0.001
**Serum BD-tau (pg/mL)** Median (IQR)	9.93 (7.61-12.01)	11.56 (9.40-16.76)	6.90 (5.10-8.20)	<0.001
**Serum p-tau217 (pg/mL)** Median (IQR)	0.415 (0.240-0.550)	0.770 (0.410-0.970)	0.145 (0.118-0.193)	<0.001
**CSF BD-tau (pg/mL)** Median (IQR)	358.48 (298.00-420.94)	687.90 (546.19-922.04)	267.94 (196.28-370.23)	<0.001
**CSF t-tau (pg/mL)** Median (IQR)	289 (213-371)	665 (515-818)	218 (190-281)	<0.001

Age, sex and biomarkers (serum BD-tau, serum p-tau217, CSF BD-tau and CSF t-tau) in the three diagnostic groups are displayed as mean±standard deviation (SD), median and interquartile range (IQR) or as percentage. Depending on the type and distribution of the data, two-sided p-values of ANOVA, Kruskal-Wallis or Chi-squared test are reported. Dunn post-hoc tests (adjustments for multiple comparisons) to compare biomarkers levels: serum BD-tau: AD vs. controls p<0.001; AD vs. ALS p=0.026; ALS vs disease controls p<0.001; CSF BD-tau: AD vs. controls p<0.001; AD vs. ALS p<0.001; ALS vs disease controls p=0.813; CSF t-tau: ALS vs. AD p<0.001, ALS vs. controls p=0.537, AD vs controls p<0.001. *AD* Alzheimer's disease, *ALS* amyotrophic lateral sclerosis, *BD-tau* brain-derived tau protein, *CSF* cerebrospinal fluid, *IQR* interquartile range, *N* number of cases, *p-tau217* phosphorylated tau protein 217, *t-tau* total tau protein

Supplementary Table 3. Demographic characteristics and biomarker distribution in the diagnostic groups in the Milan cohort.

Milan				
	**ALS**	**AD**	**Disease controls**	**p-value**
**N with serum samples**	65	17	8	
**Age (years)** mean±SD	62.6±10.3	73.1±4.8	64.4±13.8	0.048
**Female (%)**	33.8	41.2	50.0	0.618
**Serum BD-tau (pg/mL)** Median (IQR)	8.89 (7.14-12.85)	10.43 (9.69-14.52)	7.89 (5.70-11.33)	<0.001
**Serum p-tau217 (pg/mL)** Median (IQR)	0.370 (0.195-0.605)	0.600 (0.390-0.840)	0.130 (0.100-0.213)	<0.001
**CSF BD-tau (pg/mL)** Median (IQR)	273.02 (206.99-354.15)	-	230.70 (171.45-320.90)	0.212
**CSF t-tau (pg/mL)** Median (IQR)	256 (202-354)	619 (521-1099)	205 (158-229)	<0.001

Age, sex and biomarkers (serum BD-tau, serum p-tau217, CSF BD-tau and CSF t-tau) in the three diagnostic groups are displayed as mean±standard deviation (SD), median and interquartile range (IQR) or as percentage. Depending on the type and distribution of the data, two-sided p-values of ANOVA, Kruskal-Wallis or Chi-squared test are reported. Dunn's post-hoc tests (adjustments for multiple comparisons) to compare biomarkers levels: serum BD-tau: AD vs. controls p=0.024; AD vs. ALS p=0.046; ALS vs disease controls p=0.259; serum p-tau217: AD vs. controls p<0.001; AD vs. ALS p=0.010; ALS vs disease controls p=0.027.; CSF t-tau: ALS vs. AD p<0.001, ALS vs. controls p=0.349, AD vs controls p<0.001. *AD* Alzheimer's disease, *ALS* amyotrophic lateral sclerosis, *BD-tau* brain-derived tau protein, *CSF* cerebrospinal fluid, *IQR* interquartile range, *N* number of cases, *p-tau217* phosphorylated tau protein 217, *t-tau* total tau protein

Supplementary Table 4. Demographic characteristics and biomarker distribution in the diagnostic groups in the Mannheim 1 cohort.

Mannheim 1				
	**ALS**	**AD**	**Disease controls**	**p-value**
**N with serum samples**	16	-	12	
**Age (years)** mean±SD	54.6±17.3	-	58.4±19.5	0.642
**Female (%)**	37.5	-	50.0	0.508
**Serum BD-tau (pg/mL)** Median (IQR)	6.57 (5.49-9.54)	-	6.61 (5.17-7.92)	0.676
**Serum p-tau217 (pg/mL)** Median (IQR)	0.165 (0.120-0.453)	-	0.140 (0.080-0.285)	0.245
**CSF t-tau (pg/mL)** Median (IQR)	255 (191-383)	-	195 (159-391)	0.377

Age, sex and biomarkers (serum BD-tau, serum p-tau217 and CSF t-tau) in the two diagnostic groups are displayed as mean±standard deviation (SD), median and interquartile range (IQR) or as percentage. Depending on the type and distribution of the data, two-sided p-values of t-test, Mann-Whitney or Chi-squared test are reported. *AD* Alzheimer's disease, *ALS* amyotrophic lateral sclerosis, *BD-tau* brain-derived tau protein, *CSF* cerebrospinal fluid, *IQR* interquartile range, *N* number of cases, *p-tau217* phosphorylated tau protein 217, *t-tau* total tau protein

Supplementary Table 5. Demographic characteristics and biomarker distribution in the diagnostic groups in the Mannheim 2 cohort.

Mannheim 2				
	**ALS**	**AD**	**Disease controls**	**p-value**
**N with serum samples**	-	25	25	
**Age (years)** mean±SD	-	72.7±8.0	62.1±9.9	<0.001
**Female (%)**	-	64.0	56.0	0.348
**Serum BD-tau (pg/mL)** Median (IQR)	-	11.01 (9.67-16.00)	6.74 (4.43-8.33)	<0.001
**Serum p-tau217 (pg/mL)** Median (IQR)	-	0.770 (0.545-0.930)	0.150 (0.115-0.255)	<0.001
**CSF t-tau (pg/mL)** Median (IQR)	-	693 (536-857)	188 (126-279)	<0.001

Age, sex and biomarkers (serum BD-tau, serum p-tau217 and CSF t-tau) in the two diagnostic groups are displayed as mean±standard deviation (SD), median and interquartile range (IQR) or as percentage. Depending on the type and distribution of the data, two-sided p-values of t-test, Mann-Whitney or Chi-squared test are reported. *AD* Alzheimer's disease, *ALS* amyotrophic lateral sclerosis, *BD-tau* brain-derived tau protein, *CSF* cerebrospinal fluid, *IQR* interquartile range, *N* number of cases, *p-tau217* phosphorylated tau protein 217, *t-tau* total tau protein

Supplementary Table 6. Biomarker levels disaggregated by sex.

Sex	Male	Female	p-value
**Serum BD-tau (pg/mL)** Median (IQR)	8.890 (6.880-11.730)	8.670 (6.585-12.355)	0.784
**Serum p-tau217 (pg/mL)** Median (IQR)	0.390 (0.173-0.705)	0.250 (0.140-0.620)	0.005
**CSF BD-tau (pg/mL)** Median (IQR)	358.49 (261.37-478.68)	463.81 (265.73-678.94)	0.057
**CSF t-tau (pg/mL)** Median (IQR)	312 (213-517)	383 (213-668)	0.067

Biomarkers (serum BD-tau, serum p-tau217, CSF BD-tau and CSF t-tau) in the males and females are displayed as median and interquartile range (IQR). Two-sided p-values of Mann-Whitney test are reported. *BD-tau* brain-derived tau protein, *CSF* cerebrospinal fluid, *IQR* interquartile range, *p-tau217* phosphorylated tau protein 217, *t-tau* total tau protein

Supplementary Table 7. Serum BD-tau and p-tau217 distribution across diagnostic groups: one-step approach using the Simoa platform vs. two-step approach using manual BD-Tau-IP followed by Simoa (first manual run)

	ALS	AD	Disease controls	p-value
N	21	22	21	
**Serum BD-tau**				
Simoa (pg/mL) Median (IQR)	9.07 (7.33-10.56)	11.39 (9.71-14.84)	7.33 (5.15-8.41)	<0.001
BD-Tau-IP + Simoa (pg/mL) Median (IQR)	40.52 (33.29-48.92)	56.91 (47.17-69.48)	35.35 (28.24-41.43)	<0.001
Concentration Factor Median (IQR)	4.66 (4.17-5.10)	4.94 (4.31-5.31)	5.21 (4.59-5.84)	0.129
**Serum p-tau217**				
Simoa (pg/mL) Median (IQR)	0.29 (0.20-0.57)	0.86 (0.61-1.21)	0.14 (0.12-0.20)	<0.001
BD-Tau-IP + Simoa (pg/mL) (BD-p-tau217) Median (IQR)	0.36 (0.29-0.67)	2.83 (1.98-3.77)	0.32 (0.22-0.45)	<0.001
Concentration Factor Median (IQR)	1.30 (1.09-1.93)	3.15 (3.02-3.54)	2.23 (1.83-2.60)	<0.001
**Ratio BD-tau/BD-p-tau217 (BD-Tau-IP + Simoa)** Median (IQR)	96.49 (69.50-145.30)	19.83 (17.30-30.85)	108.00 (88.54-144.29)	<0.001

Biomarkers (serum BD-tau, serum BD-tau after BD-Tau-IP, serum p-tau217, serum BD-p-tau217 and the ratio BD-tau/BD-p-tau217) in the three diagnostic groups are displayed as median and interquartile range (IQR). Two-sided p-values of Kruskal-Wallis are reported. Dunn post-hoc tests (adjustments for multiple comparisons) to compare biomarkers levels: serum BD-tau (Simoa): AD vs. controls p<0.001; AD vs. ALS p=0.025; ALS vs disease controls p=0.069; serum BD-tau after BD-Tau-IP: AD vs. controls p<0.001; AD vs. ALS p=0.004; ALS vs disease controls p=0.484; serum p-tau217 (Simoa): AD vs. controls p<0.001; AD vs. ALS p=0.007; ALS vs disease controls p=0.006; Serum BD-p-tau217: AD vs. ALS p<0.001, ALS vs. controls p=0.789, AD vs controls p<0.001, ratio BD-tau/BD-p-tau217: AD vs. ALS p<0.001, ALS vs. controls p=1.000, AD vs controls p<0.001. *AD* Alzheimer's disease, *ALS* amyotrophic lateral sclerosis, *BD-p-tau217* brain-derived phosphorylated tau protein 217, *BD-tau* brain-derived tau protein, *BD-Tau-IP* brain-derived tau immunoprecipitation, *IQR* interquartile range, *N* number of cases, *p-tau217* phosphorylated tau protein 217

Supplementary Table 8. Comparative analysis of the diagnostic accuracy of serum BD-tau and p-tau217: one-step approach using the Simoa platform vs. two-step approach using manual BD-Tau-IP followed by Simoa (first manual run)

	AUC		
**Biomarker**	**AD vs. ALS**	**AD vs. Controls**	**ALS vs. controls**
**Serum BD-Tau**			
Simoa	0.777 ± 0.072	0.896 ± 0.054	0.748 ± 0.077
BD-Tau-IP + Simoa	0.818 ± 0.065	0.879 ± 0.060	0.660 ± 0.086
DeLong test p-value	0.519	0.532	0.151
**Serum p-tau217**			
Simoa	0.837 ± 0.064	0.981 ± 0.020	0.848 ± 0.062
BD-Tau-IP + Simoa (BD-p-tau217)	0.972 ± 0.023	0.989 ± 0.012	0.644 ± 0.086
DeLong test p-value	0.004	0.382	0.008
**Ratio BD-tau / BD-p-tau217 (BD-Tau-IP + Simoa)**			
Ratio BD-tau/BD-p-tau217 (BD-Tau-IP + Simoa)	0.955 ± 0.031	0.981 ± 0.020	0.583 ± 0.091
DeLong p-value vs. BD-Tau-IP BD-tau	0.064	0.111	0.565
DeLong p-value vs. BD-p-tau217	0.161	0.364	0.326

ROC analyses were performed to calculate the area under the curve (AUC) for the biomarkers (serum BD-tau, serum BD-tau after BD-Tau-IP, serum p-tau217, serum BD-p-tau217 and the ratio) when comparing the three diagnostic groups. The DeLong test was used to compare the AUCs. Two-sided p-values are reported. *AD* Alzheimer's disease, *ALS* amyotrophic lateral sclerosis, *BD-p-tau217* brain-derived phosphorylated tau protein 217, *BD-tau* brain-derived tau protein, *BD-Tau-IP* brain-derived tau immunoprecipitation, *p-tau217* phosphorylated tau protein 217

Supplementary Table 9. Serum p-tau217 distribution across diagnostic groups: one-step approach using the Simoa platform vs. two-step approach using manual BD-Tau-IP followed by Simoa (second manual run)

	**ALS**	**AD**	**Disease controls**	**p-value**
**N**	45	39	14	
**Serum p-tau217**				
Simoa (pg/mL) Median (IQR)	0.38 (0.26-0.57)	0.65 (0.38-0.93)	0.14 (0.10-0.22)	<0.001
BD-Tau-IP + Simoa (pg/mL) (BD-p-tau217) Median (IQR)	0.44 (0.23-0.70)	2.56 (1.24-3.93)	0.39 (0.29-0.66)	<0.001
Concentration Factor Median (IQR)	1.31 (0.77-1.95)	3.64 (2.67-4.23)	3.00 (2.45-3.15)	<0.001

Serum p-tau217 and serum BD-p-tau217 in the three diagnostic groups are displayed as median and interquartile range (IQR). Two-sided p-values of Kruskal-Wallis are reported. Dunn post-hoc tests (adjustments for multiple comparisons) to compare biomarkers levels: serum p-tau217 (Simoa): AD vs. controls p<0.001; AD vs. ALS p=0.046; ALS vs disease controls p=0.001; Serum BD-p-tau217: AD vs. ALS p<0.001, ALS vs. controls p=1.000, AD vs controls p<0.001. *AD* Alzheimer's disease, *ALS* amyotrophic lateral sclerosis, *BD-p-tau217* brain-derived phosphorylated tau protein 217, *BD-tau* brain-derived tau protein, *BD-Tau-IP* brain-derived tau immunoprecipitation, *IQR* interquartile range, *N* number of cases, *p-tau217* phosphorylated tau protein 217

Supplementary Table 10. Comparative analysis of the diagnostic accuracy of serum p-tau217: one-step approach using the Simoa platform vs. two-step approach using manual BD-Tau-IP followed by Simoa (second manual run)

	AUC		
**Biomarker**	**AD vs. ALS**	**AD vs. controls**	**ALS vs. controls**
**Serum p-tau217**			
Simoa	0.662 ± 0.060	0.940 ± 0.036	0.836 ± 0.061
BD-Tau-IP + Simoa (BD-p-tau217)	0.901 ± 0.035	0.930±0.035	0.550 ± 0.078
DeLong test p-value	<0.001	0.784	<0.001

ROC analyses were performed to calculate the area under the curve (AUC) for the biomarkers (serum p-tau217, serum BD-p-tau217) when comparing the three diagnostic groups. The DeLong test was used to compare the AUCs. Two-sided p-values are reported. *AD* Alzheimer's disease, *ALS* amyotrophic lateral sclerosis, *BD-p-tau217* brain-derived phosphorylated tau protein 217, *BD-tau* brain-derived tau protein, *BD-Tau-IP* brain-derived tau immunoprecipitation, *p-tau217* phosphorylated tau protein 217

Supplementary Table 11. Demographic, clinical and laboratory data of ALS patients from Halle and Milan (n = 123)

**Age (years) at blood collection** mean ±SD	63.24± 10.51
**Female** N (%)	38 (30.9)
**BMI** mean ±SD	24.18 ± 3.76
**Site of symptom onset** N (%)<br>bulbar<br>spinal: upper limbs, trunk, lower limbs<br>generalised<br>cognitive	 25 (20.3)<br>93 (75.6): 46 (37.4), 2 (1.6), 45 (36.6)<br>4 (3.3)<br>1 (0.8)
**Disease duration at blood collection (months)** mean ±SD	22.20 ± 20.25
**Clinical phenotype** N (%)<br>classic<br>bulbar<br>UMN-predominant<br>LMN-predominant	 77 (62.6)<br>16 (13.0)<br>9 (7.3)<br>21 (17.1)
**Diagnosis according to revised El Escorial criteria** N (%)<br>Definite<br>Probable<br>Probable laboratory-supported<br>Possible	 29 (23.6)<br>28 (22.8)<br>45 (36.6)<br>21 (17.1)
**King's staging system** N (%)<br>1, 2, 3, 4	 4 (3.3), 62 (50.4), 44 (35.8), 13 (10.6)
**ALSFRS-R score** mean ±SD	39.08 ± 7.06
**UMN involvement** N (%)<br>Bulbar, cervical, lumbosacral<br>**LMN involvement** N (%)<br>Bulbar, cervical, thoracic, lumbosacral	 54 (43.9), 91 (74.0), 91 (74.0)<br><br>73 (59.3), 114 (92.7), 49 (39.8), 113 (92.0)
**Disease progression rate at blood sampling** mean ±SD	0.71 ± 0.60
**ECAS total score** mean ±SD	101.05 ± 17.38
**MoCA score** mean ±SD	22.94±3.56
**Blood Creatine Kinase (U/L)** Median (IQR) (normal values <171 U/L)	137.0 (66.6-254.7)
**Blood Troponin T (ng/L)** Median (IQR) (normal values <14 ng/L)	49.0 (16.2-73.3)
**Blood Myoglobin (µg/L)** Median (IQR) (normal values 25-72 µg/L)	43.0 (30.6-89.5)

ALS amyotrophic lateral sclerosis, *ALSFRS-R* ALS Functional Rating Scale-Revised, *BMI* body mass index, *ECAS* Edinburgh Cognitive and Behavioural ALS Screen, *IQR* interquartile range, *LMN* lower motor neuron, *MoCA* Montreal cognitive assessment, *N* number of cases, *SD* standard deviation, *UMN* upper motor neuron

Supplementary Table 12. Tau isoform ratios across frontal cortex, spinal cord, and muscle

Tissue	Diagnostic groups	N	BD-tau/t-tau, median (IQR)	Big tau/t-tau, median (IQR)
Frontal cortex	AD, ALS, disease controls	16	0.993 (0.765-1.116)	0.688 (0.300-1.226)
Spinal cord	ALS, disease controls	11	0.681 (0.466-1.043)	0.545 (0.407-0.936)
Muscle	ALS, disease controls	10	0.206 (0.152-0.273)	1.749 (1.631-2.038)

BD-tau/t-tau and big tau/t-tau ratios were calculated by normalizing the PRM intensities of the respective isoform-specific peptides to the intensities of peptides common to all tau isoforms. Ratios are based on relative peptide intensities and do not represent molar fractions of tau isoforms; they are intended for comparison across tissues rather than for estimating the absolute proportion of each isoform within a tissue. Data were pooled across diagnostic groups within each tissue and presented descriptively as median and interquartile range (IQR), without formal statistical comparisons, given the exploratory nature and limited sample size of the tissue analyses. *AD* Alzheimer's disease, *ALS* amyotrophic lateral sclerosis; *BD-tau* brain-derived tau protein, *IQR* interquartile range; *PRM* parallel reaction monitoring, *t-tau* total tau.

Supplementary Table 13. BD-Tau-IP procedural parameters across three protocols

Parameter	Manual IP 1st run	Manual IP 2nd run	Semi-automated IP
Subjects included	21 ALS, 22 AD, 21 controls	45 ALS, 39 AD, 14 controls	50 ALS, 60 AD, 39 controls
Starting serum volume (per patient)	2 × 500 µL (1,000 µL total)	200 µL	2 × 400 µL (800 µL total)
Antibody-functionalized magnetic beads	50 µL	25 µL	50 µL
Amount of 2B8 antibody (per patient)	10 µg	5 µg	10 µg
Elution volume (per patient)	2 × 50 µL (100 µL total)	30 µL	2 × 75 µL (150 µL total)
Dilution factor of eluate for BD-tau measurement (Simoa direct assay)	1:4	n/a	1:4
Dilution factor of eluate for p-tau217 measurement (Simoa direct assay)	1:3	1:5	1:3
Concentration of the analyte in the diluted eluate loaded onto the Simoa, relative to native serum - BD-tau*	~2.5×	n/a	~1.3×
Concentration of the analyte in the diluted eluate loaded onto the Simoa, relative to native serum – p-tau217*	~3.3×	~1.3×	~1.8×
Handling	Manual	Manual	CyBio FeliX liquid handling instrument
IP eluate storage	−80 °C until Simoa analysis	−80 °C until Simoa analysis	−80 °C until Simoa analysis

* these values were calculated from the input serum volume, the elution volume and the dilution applied to the eluate prior to Simoa measurement, assuming complete recovery and are therefore not directly comparable with the measured concentration factors reported in Table 2 (main manuscript), Supplementary Tables 7 and 9. True enrichment factors are different, as recovery during immunoprecipitation is incomplete and remains unquantified at this stage.

AD Alzheimer's disease, *ALS* amyotrophic lateral sclerosis, *BD-tau* brain-derived tau protein, *IP* immunoprecipitation, *p-tau217* phosphorylated tau protein 217

Supplementary Table 14. Characteristics of participants with available post-mortem brain tissue and of the subset with additional spinal cord samples

Subject No.	Diagnosis	Age at death (years)	Sex	Post-mortem interval (hours)	AD neuropathologic changes (NIA-AA)
1[¥]	AD	78	F	72	High
2	AD	78	M	48	Intermediate
3[¥]	AD	75	M	72	Intermediate
4	AD	72	F	12	Intermediate
5*[¥]	C9ORF72-associated ALS	48	F	192	Not present
6*	C9ORF72-associated ALS	75	M	24	Not present
7*	C9ORF72-associated ALS+FTLD	61	M	24	Not present
8*[¥]	Sporadic ALS	57	M	96	Not present
9	Sporadic ALS	43	M	48	Not present
10*	Sporadic ALS	61	M	24	Low
11*[¥]	Sporadic ALS	56	F	72	Low
12*[¥]	Disease control	57	M	144	Not present
13*[¥]	Disease control	64	M	96	Not present
14*	Disease control	35	M	48	Not present
15*	Disease control	59	M	24	Not present
16*[¥]	Disease control	67	F	96	Low

*subjects with paired brain and spinal cord samples

[¥] subjects with post-mortem interval > 48 hours

AD Alzheimer's disease, *ALS* amyotrophic lateral sclerosis, *C9ORF72* chromosome 9 open reading frame 72, *F* female, *FTLD* frontotemporal lobar degeneration, *M* male, *NIA-AA* National Institute on Aging–Alzheimer's Association

Supplementary Table 15. Description of ALS cases and disease controls, who underwent muscle biopsy

Subject No.	Diagnosis	Age at onset (years)	Age at biopsy (years)	Sex	Muscle biopsy site and side
1	possible ALS	34	35	M	biceps brachii, left
2	definite ALS	39	40	F	vastus lateralis, left
3	possible ALS	52	53	F	biceps brachii, right
4	definite ALS	68	69	M	biceps brachii, right
5	possible ALS	75	76	M	deltoideus, right
6	exercise intolerance	24	42	M	vastus lateralis, right
7	myalgia, cramps	45	48	F	vastus lateralis, right
8	Myalgia	39	42	F	biceps brachii, left
9	exercise intolerance	adolescence	45	M	biceps brachii, left
10	exercise intolerance	38	40	F	biceps brachii, right

ALS amyotrophic lateral sclerosis, *M* male, *F* female

Supplementary Table 16. PRM settings for targeted mass spectrometry

Peptide	specific for	m/z	Z	FAIMS CV (V)	Ions analyzed	Used for MS analysis after
DEAAGHVTQARMoxVSK	BD-tau	539.267	3	-65	y9+, y8+, y7+, y5+, b6+	pan-tau-IP<br>p-tau217-IP
VSTEIPASEPDGPSVGRAK	big tau	632.994	3	-65	y14+, y12+, y11+, y10+, y8+, y7+	pan-tau-IP<br>p-tau217-IP
DTEGGRHAPELLK	big tau	474.916	3	-50	y11++, y10++, y5+, b8+	pan-tau-IP
LTFRENAK	t-tau	489.772	2	-50	y7+, y6+, b7+, b6+	pan-tau-IP
IATPRGAAPPGQK	t-tau	421.910	3	-50	y12++, y11++, y6+, y5+, y4+ (p+++, p(M+1)+++, p(M+2)+++)	pan-tau-IP,<br>p-tau217-IP

BD-tau brain-derived tau protein, *IP* immunoprecipitation, *MS* mass spectrometry, *PRM* parallel reaction monitoring, *p-tau217* phosphorylated tau protein 217, *t-tau* total tau protein

Supplementary Figure 1.

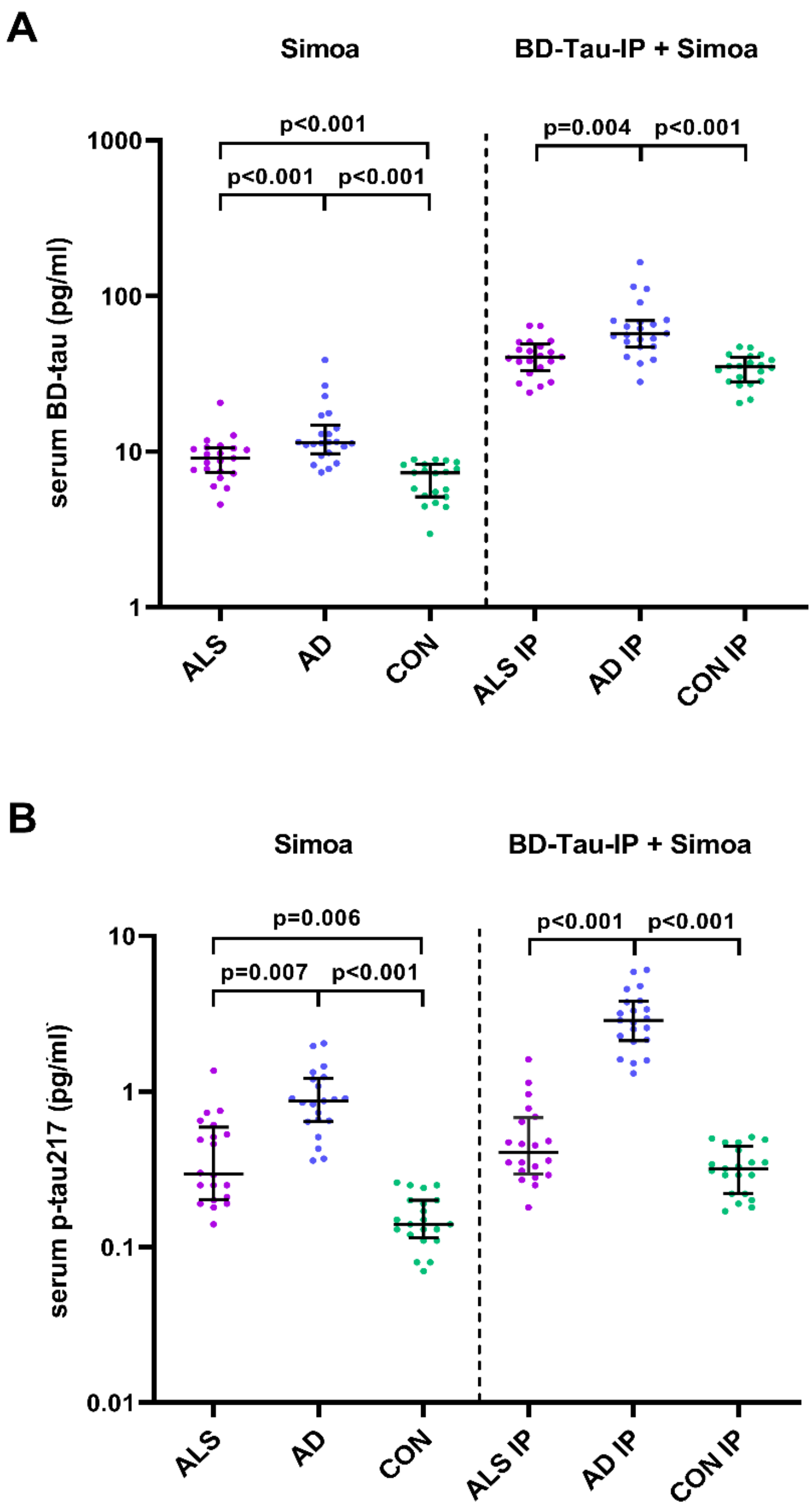

Serum BD-tau and p-tau217 distribution across diagnostic groups: one-step approach using the Simoa platform vs. two-step approach using manual BD-Tau-IP followed by Simoa (first run)

A. Distribution of serum BD-tau in a sub-cohort of ALS (n = 21), AD subjects (n = 22) and disease controls (CON, n = 21): one-step approach using the Simoa platform for direct measurement vs. two-step approach using manual BD-Tau-IP followed by Simoa. B. Distribution of serum p-tau217 in a sub-cohort of ALS (n = 21), AD subjects (n = 22) and disease controls (n = 21): one-step approach using the Simoa platform for direct

measurement vs two-step approach using manual BD-Tau-IP followed by Simoa. Y-axis shown on a logarithmic scale. Dots represent single data points. Horizontal lines represent the median values, the lower and upper lines correspond to the first and third quartiles, and the vertical line is the interquartile range. Biomarker differences between groups were assessed by Mann-Whitney or Kruskal-Wallis followed by Dunn's post hoc test (adjustment for multiple comparisons). Two-sided p-values are reported. *AD* Alzheimer's disease, *ALS* amyotrophic lateral sclerosis, *BD-tau* brain-derived tau protein, *BD-Tau-IP* brain-derived tau immunoprecipitation, *CON* disease controls, *IP* immunoprecipitation, *p-tau217* phosphorylated tau protein 217. Source data are provided as a Source Data file.

Supplementary Figure 2.

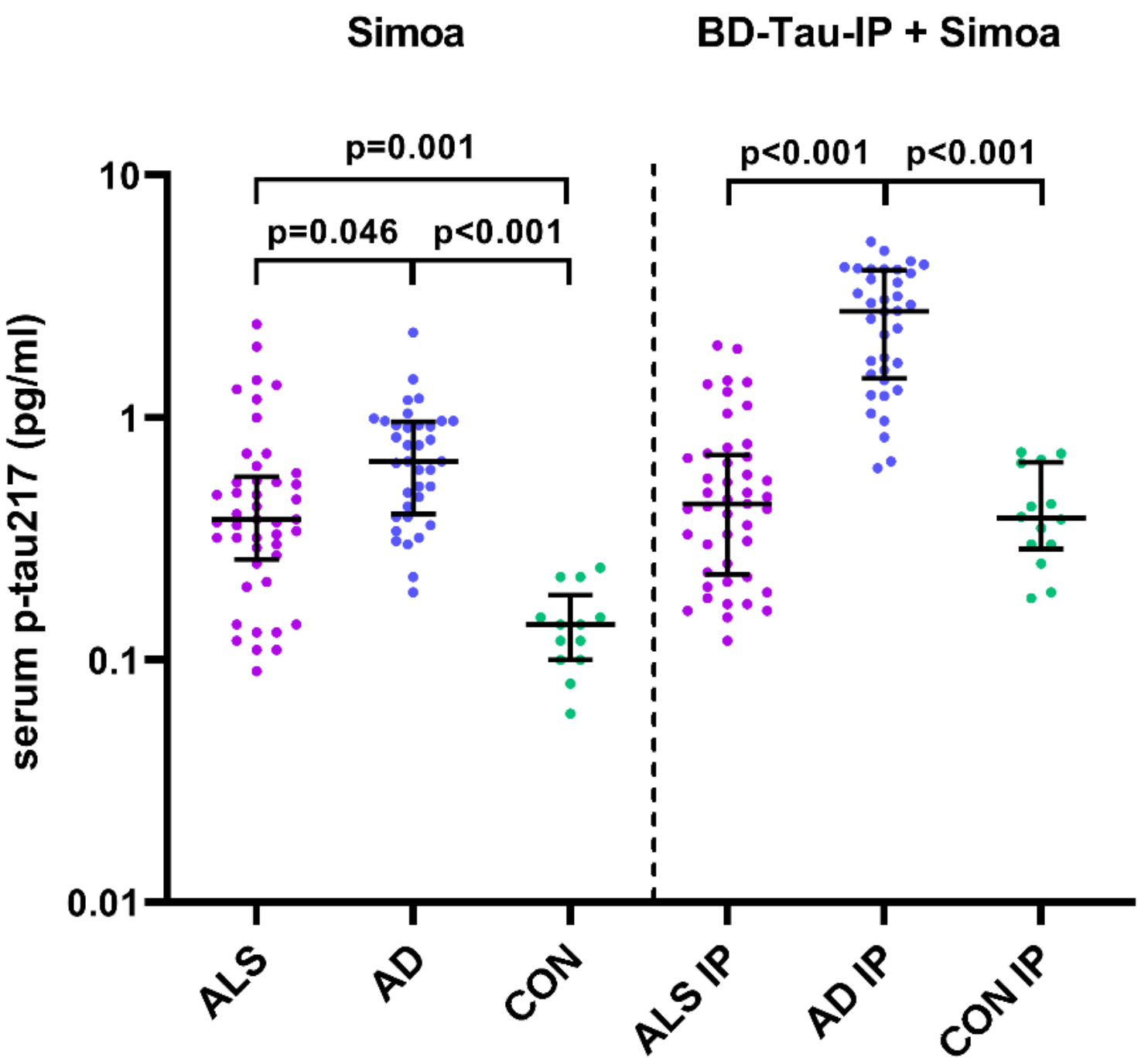

Serum p-tau217 distribution across diagnostic groups: one-step approach using the Simoa platform vs two-step approach using manual BD-Tau-IP followed by Simoa (second run)

Distribution of serum p-tau217 in a sub-cohort of ALS (n = 45), AD subjects (n = 39) and disease controls (CON, n = 14): one-step approach using the Simoa platform for direct measurement vs. two-step approach using manual BD-Tau-IP followed by Simoa. Y-axis shown on a logarithmic scale. Dots represent single data points. Horizontal lines represent the median values, the lower and upper lines correspond to the first and third quartiles, and the vertical line is the interquartile range. Biomarker differences between groups were assessed by Mann-Whitney or Kruskal-Wallis followed by Dunn's post hoc test (adjustment for multiple comparisons). Two-sided p-values are reported. *AD* Alzheimer's disease, *ALS* amyotrophic lateral sclerosis, *BD-Tau-IP* brain-derived tau immunoprecipitation, *CON* disease controls, *IP* immunoprecipitation, *p-tau217* phosphorylated tau protein 217. Source data are provided as a Source Data file.

Supplementary Figure 3.

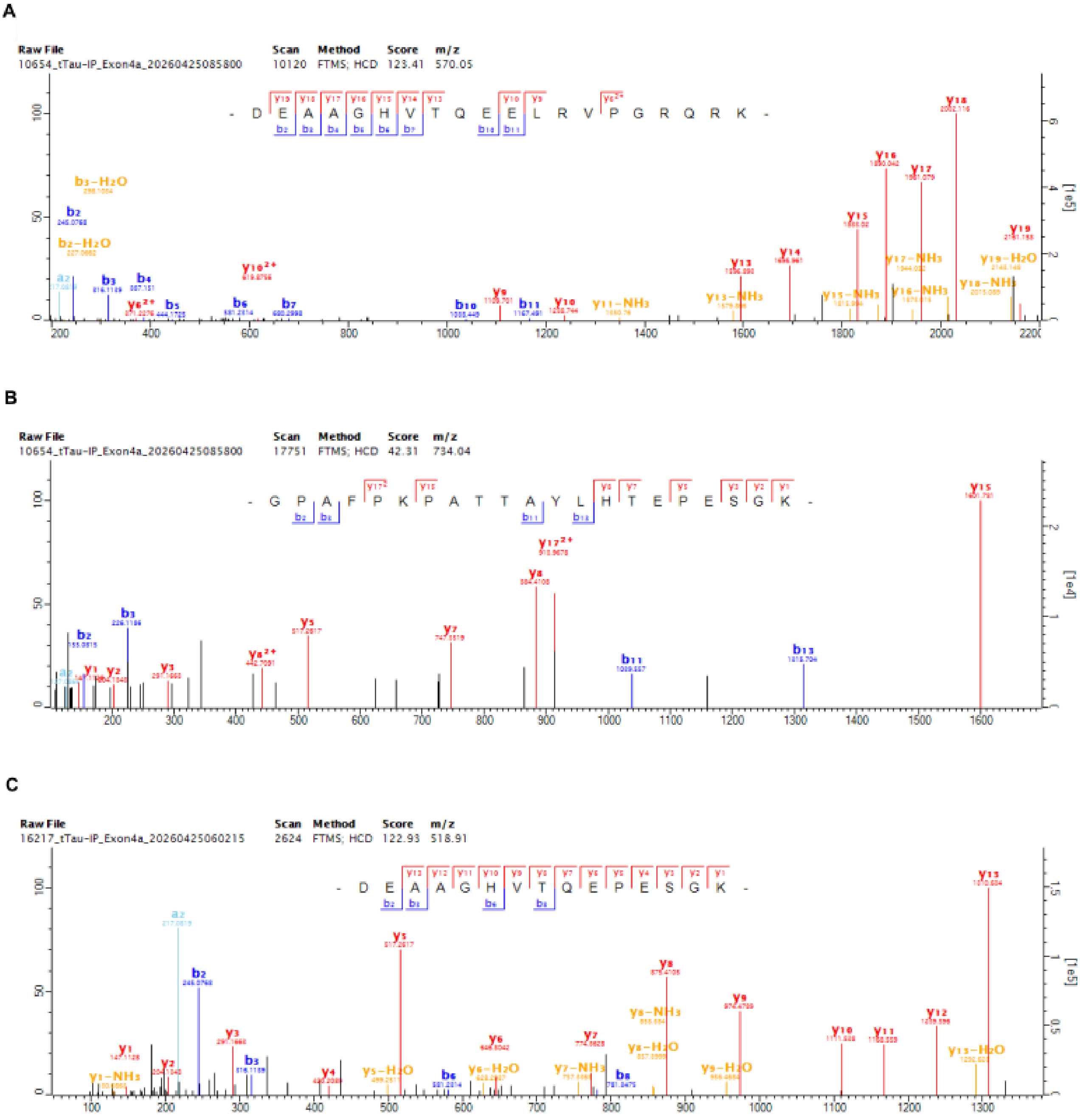

Identification of exon 4a-L and exon 4a-S isoforms in muscle tissue by untargeted mass spectrometry

Shown are spectra of peptides spanning the (**A**) exon 4-to-exon 4a-long junction (specific for big tau with exon 4a-L), (**B**) the exon 4a-long-to-exon 4a junction (specific for big tau with exon 4a-L) and (**C**) the exon 4-to-exon 4a junction (specific for big tau with exon 4a-S), indicating the co-expression of both exon 4a-L and exon 4a-S isoforms in muscle tissue. The peptides were identified by searching the untargeted mass spectrometry data against the mentioned databases, including protein isoforms extended with the sequence of big tau with exon 4a-long, as described by Koppisetti et al.[11], using MaxQuant.

Supplementary Figure 4.

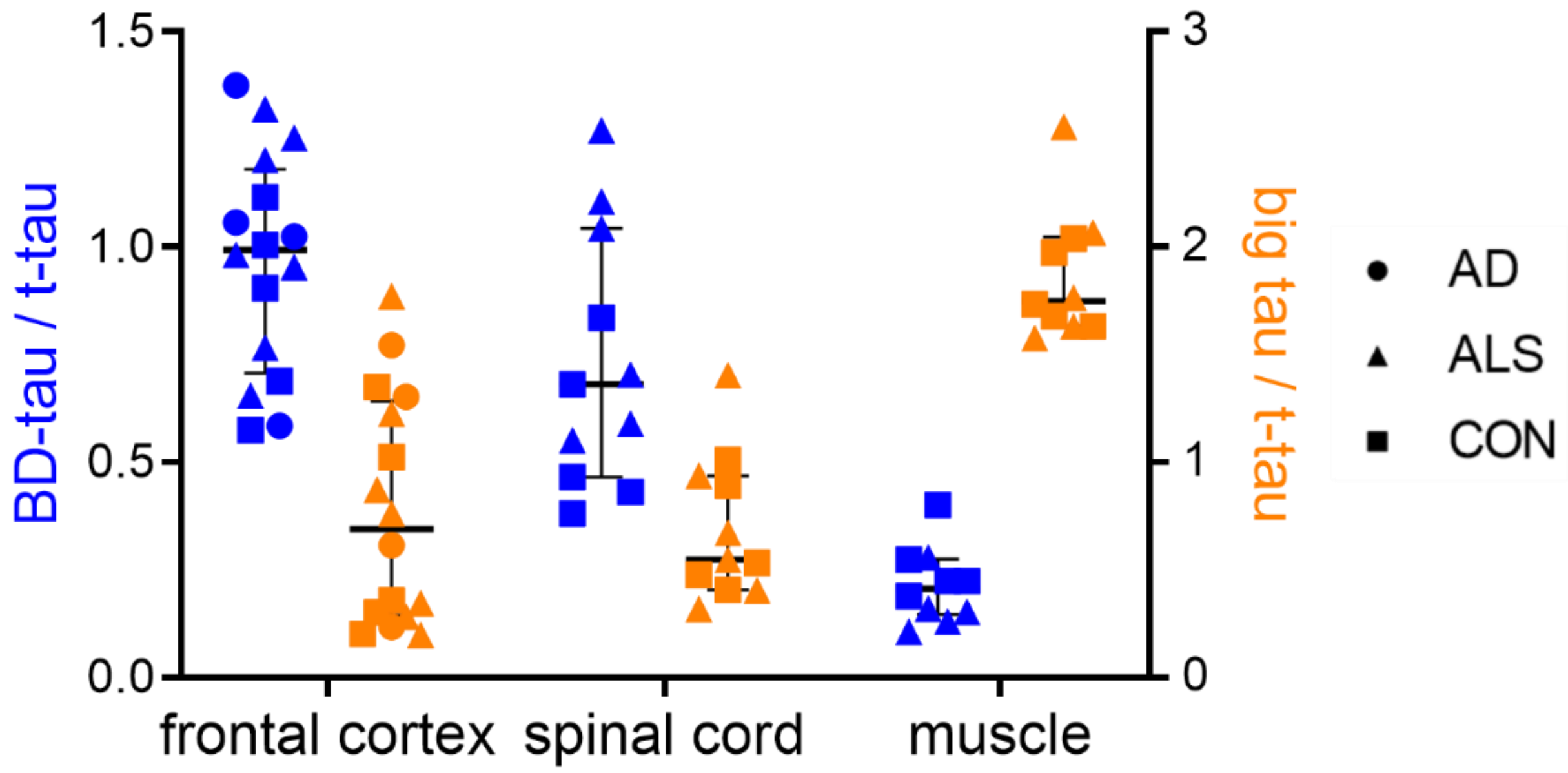

Distribution of BD-tau and big tau relative to total tau, grouped by tissue.

Peptides specific for BD-tau and big tau were measured by targeted mass spectrometry in samples obtained after pan-tau immunoprecipitation of protein extracts from frontal cortex (AD, n = 4; ALS, n = 7; disease controls, n = 5), spinal cord (ALS, n = 6; disease controls, n = 5) and skeletal muscle (ALS, n = 5; disease controls, n = 5). BD-tau– and big tau–specific peptide intensities were normalised to the intensities of peptides common to all tau isoforms. Ratios are based on relative peptide intensities and do not represent molar fractions of tau isoforms; they are intended for comparison across tissues rather than for estimating the absolute proportion of each isoform within a tissue. For each tissue, BD-tau/t-tau ratios are shown in blue (left y-axis) and big tau/t-tau ratios in orange (right y-axis). Each symbol represents an individual sample, and its shape indicates the diagnostic group (circles, AD; triangles, ALS; squares, disease controls). Horizontal lines represent the median values, the lower and upper lines correspond to the first and third quartiles, and the vertical line is the interquartile range. Data are presented descriptively, without formal statistical comparisons. *AD* Alzheimer's disease, *ALS* amyotrophic lateral sclerosis, *BD-tau* brain-derived tau protein, *CON* disease controls, *t-tau* total tau. Source data are provided as a Source Data file.

Supplementary Figure 5.

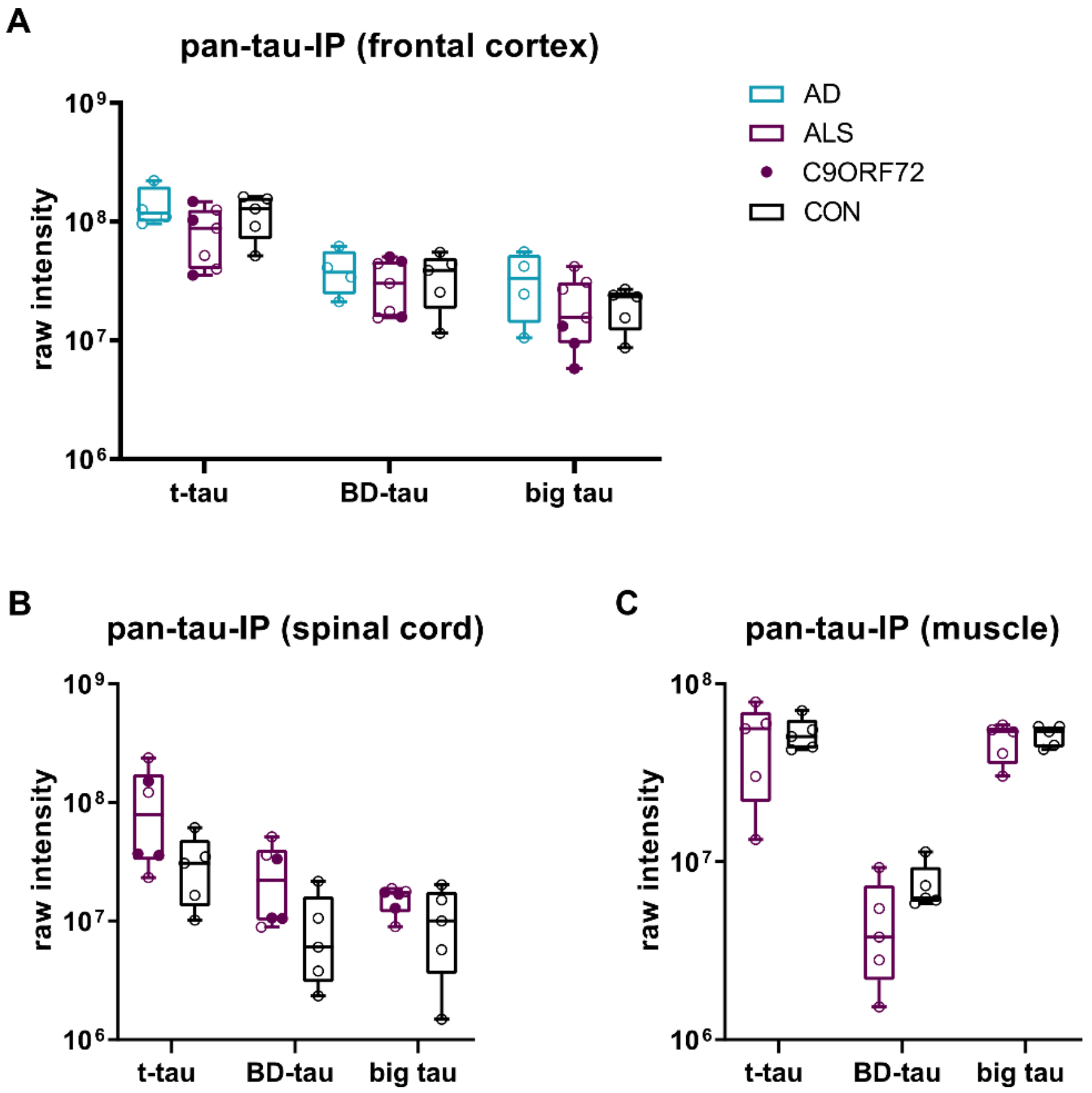

Raw peptide intensities after pan-tau immunoprecipitation across frontal cortex, spinal cord and muscle.

Shown are the raw intensities of tau peptides measured in digested eluates from pan-tau immunoprecipitation (pan-tau-IP) followed by targeted mass spectrometry of frontal cortex, spinal cord, and muscle protein extracts, without normalization to common tau peptides. For t-tau and big tau, the mean of the two specific peptides is shown. Due to different protein input amounts across tissues, raw intensities are not directly comparable between tissue compartments. Frontal cortex samples include AD (n = 4), ALS (n = 7) and disease controls (n = 5); spinal cord samples include ALS (n = 6) and disease controls (n = 5); muscle samples include ALS (n = 5) and disease controls (n = 5). Each dot represents one tissue sample. Data are presented descriptively, without formal statistical comparisons. *AD* Alzheimer's disease, *ALS* amyotrophic lateral sclerosis, *big tau* big tau protein, *BD-tau* brain-derived tau protein, *CON* disease controls, *C9ORF72* chromosome 9 open reading frame 72, *IP* immunoprecipitation, *t-tau* total tau. Source data are provided as a Source Data file.

Supplementary References

1. Abu-Rumeileh, S. *et al.* Phosphorylated tau 181 and 217 are elevated in serum and muscle of patients with amyotrophic lateral sclerosis. *Nat. Commun.* **16**, 2019 (2025). https://doi.org/10.1038/s41467-025-57144-7
2. Braak, H., Thal, D. R., Ghebremedhin, E. & Del Tredici, K. Stages of the Pathologic Process in Alzheimer Disease: Age Categories From 1 to 100 Years. *J Neuropathol Exp Neurol* **70**, 960–969 (2011).
3. Chiò, A. *et al.* Phenotypic heterogeneity of amyotrophic lateral sclerosis: a population-based study. *J. Neurol. Neurosurg. Psychiatry* **82**, 740–746 (2011). https://doi.org/10.1136/jnnp.2010.235952
4. Abu-Rumeileh, S. *et al.* Diagnostic-prognostic value and electrophysiological correlates of CSF biomarkers in amyotrophic lateral sclerosis. *J. Neurol.* **267**, 1699–1708 (2020). https://doi.org/10.1007/s00415-020-09761-z
5. Hyman, B. T. *et al.* National Institute on Aging–Alzheimer's Association guidelines for the neuropathologic assessment of Alzheimer's disease. *Alzheimers Dement*. **8**, 1–13 (2012). https://doi.org/10.1016/j.jalz.2011.10.007
6. Montine, T. J. *et al.* National Institute on Aging–Alzheimer's Association guidelines for the neuropathologic assessment of Alzheimer's disease: a practical approach. *Acta Neuropathol.* **123**, 1–11 (2012). https://doi.org/10.1007/s00401-011-0910-3
7. Brooks, B. R. *et al.* El Escorial revisited: revised criteria for the diagnosis of amyotrophic lateral sclerosis. *Amyotroph. Lateral Scler. Other Motor Neuron Disord.* **1**, 293–299 (2000). https://doi.org/10.1080/146608200300079536
8. Rascovsky, K. *et al.* Sensitivity of revised diagnostic criteria for the behavioural variant of frontotemporal dementia. *Brain* **134**, 2456–2477 (2011). https://doi.org/10.1093/brain/awr179
9. Gorno-Tempini, M. L. *et al.* Classification of primary progressive aphasia and its variants. *Neurology* **76**, 1006–1014 (2011). https://doi.org/10.1212/WNL.0b013e31821103e6
10. Cox, J. *et al.* Accurate proteome-wide label-free quantification by delayed normalization and maximal peptide ratio extraction, termed MaxLFQ. *Mol Cell Proteomics* **13**, 2513-2526 (2014). doi: 10.1074/mcp.M113.031591.
11. Koppisetti, R.K. *et al.* Distribution of Big Tau Isoforms in the Human Central and Peripheral Nervous System. *Ann Neurol.* (2026). doi: 10.1002/ana.78300.
12. UniProt Consortium. UniProt: a worldwide hub of protein knowledge. *Nucleic Acids Res.* **47**, D506-D515 (2019). doi: 10.1093/nar/gky1049.
