# Supplementary material for: Tau isoform mapping across tissues and biofluids reveals a signature distinguishing Alzheimer’s disease from amyotrophic lateral sclerosis
Source: Nat Commun. 2026 Oct 22;17:10745. doi: 10.1038/s41467-026-78390-3 (PMC13691104; doi:10.1038/s41467-026-78390-3)
Supplement: Supplementary file 2 [file 41467_2026_78390_MOESM2_ESM.pdf]

nature portfolio

Peer Review File

Tau isoform mapping across tissues and biofluids reveals a signature distinguishing Alzheimer's disease from amyotrophic lateral sclerosis

Corresponding Author: Professor Markus Otto

This file contains all reviewer reports in order by version, followed by all author rebuttals in order by version.

Version 0:

Reviewer comments:

Reviewer #1

(Remarks to the Author)
This manuscript addresses a clinically important issue in the field of blood-based biomarkers for neurodegenerative diseases, namely the biological and analytical specificity of circulating phosphorylated tau species. By combining ultrasensitive immunoassays, a selective brain-derived tau immunoprecipitation strategy, and targeted mass spectrometry across multiple tissue types, the authors provide a compelling and mechanistically grounded investigation of the peripheral contribution to blood tau measurements, particularly in amyotrophic lateral sclerosis.
The study is well designed, with multicentre cohorts, and builds coherently on the authors' previous work. Importantly, it delivers a clear message with direct implications for the interpretation and clinical deployment of plasma tau biomarkers in Alzheimer's disease, especially in populations with concurrent or suspected neuromuscular disorder. Although the manuscript is certainly important to the field, addressing the following comment would improve its clarity and transparency, for clinical implementation in selective situations.

Major Comments :
- The authors conclude that the BD-Tau-IP enables selective measurement of brain-derived tau species. However, as acknowledged in the Methods section, the absolute recovery of BD-tau and BD-p-tau217 after immunoprecipitation is unknown, and biomarker concentrations are therefore reported as relative values. It would strengthen the manuscript to discuss more explicitly:
1) The reproducibility of the BD-Tau-IP procedure (both on AD and control subjects)
2) If available, the potential batch-to-batch variability of antibody-functionalized magnetic beads
3) The possibility of developing quantitative mass spectrometry measurements (e.g., using AQUA peptides or other strategies).

Given the current emphasis in the field on assay harmonization and standardization of blood-based tau biomarkers, as well as the importance of quantitation for longitudinal monitoring, this point will be particularly relevant for translation into routine practice.

- The authors report that serum BD-tau measured with the one-step Simoa assay correlates with disease severity and progression in ALS, whereas these associations disappear after BD-Tau-IP.
This observation strongly suggests that the initial correlations may reflect peripheral muscle-derived tau rather than central neurodegeneration. Expanding the discussion on this point would be valuable, particularly in comparison with neurofilament light chain (NfL), which is more firmly established as a marker of axonal damage.

- A major strength of the study is the use of targeted mass spectrometry to distinguish BD-tau from big tau in muscle tissue. However, no mass spectrometric validation is provided for serum or CSF samples, where the immunoassays are ultimately applied. While the technical challenges are acknowledged, this remains an important limitation. A forward-looking discussion on the feasibility of mass spectrometry–based validation of BD-tau or BD-p-tau217 in blood would further enhance the methodological rigor of the manuscript.

- While the BD-Tau immunoprecipitation (BD-Tau-IP) represents a central methodological innovation of this study, the level

of procedural detail currently provided is insufficient to ensure full reproducibility by independent laboratories.

In particular, the manuscript would benefit from a clearer and more standardized description of:
1) the starting serum volume used for each IP,
2) the amount of antibody and magnetic beads applied per sample,
3) the final elution volume,
4) the resulting concentration or dilution factor relative to native serum,
5) the handling and storage conditions of IP eluates prior to Simoa analysis.
This information is especially important given the observed differences between manual and semi-automated IP procedures and the authors' conclusion that further standardization is required before clinical implementation. Providing these details, either in the main text or in the Supplementary Methods, would substantially enhance methodological transparency and facilitate external validation.

- Clinical positioning and proposed algorithm for the use of BD-p-tau217 measurement. While the analytical advantages of the BD-Tau-IP approach are convincingly demonstrated, the clinical added value of systematically applying this protocol in all diagnostic scenarios remains unclear. Given its increased pre-analytical complexity, cost, and limited scalability, it is unlikely that BD-p-tau217 measurement after immunoprecipitation would be justified as a universal first-line test.
To strengthen the translational relevance of the study, the authors are encouraged to propose a pragmatic clinical and/or analytical algorithm delineating the specific situations in which BD-p-tau217 assessment would be particularly informative.

Providing a decision framework—whether based on clinical features, biomarker ranges, or both—would help readers understand when and why the additional analytical effort of BD-Tau-IP is justified, and would greatly facilitate implementation in both research settings and eventually future real-world diagnostic workflows.

Minor comments :
- Please, use the more widely adopted « p-Tau217 » or « p-tau217 » terminology instead of p-tau 217
- The terms BD-tau, small tau, and brain-derived tau are used interchangeably throughout the manuscript. Providing a clear and operational definition early in the Introduction would improve clarity.

Reviewer #2

(Remarks to the Author)
I co-reviewed this manuscript with one of the reviewers who provided the listed reports. This is part of the Nature Communications initiative to facilitate training in peer review and to provide appropriate recognition for Early Career Researchers who co-review manuscripts.

Reviewer #3

(Remarks to the Author)
The study "Selective detection of tau isoforms across brain, muscle and blood in Alzheimer´s disease and amyotrophic lateral sclerosis" by Samir Abu-Rumeileh et al. investigates the origin of the blood phosphorylated tau (p-tau) 217 in amyotrophic lateral sclerosis (ALS), that is an diagnostic biomarker for Alzheimer's disease (AD) and is also elevated in ALS patient plasma. They hypothesize the likely source of the increased p-tau217 originates from peripheral tau that are released from denervated muscle fibers.
To further prove their point, they measure a novel tau fragment in the serum using commercial immunoassay, brain derived tau (BD-tau) along with p-tau 217 in a cohort of ALS (n=139), AD (n=101) and disease controls (n=75). They also performed pre-analytical magnetic bead BD-tau immunoprecipitation (BD-tau IP). In their direct immunoassay, they found serum BD-tau and p-tau217 were elevated in both AD and ALS compared to controls. Whereas, following BD-tau-IP, serum BD-tau and p-tau 217 were observed exclusively in AD. The authors also proved the BD-tau and peripheral tau (big tau) species in brains and muscle samples by targeted mass spectrometry.
This study is interesting and aims at resolving the long-standing biomarker related question about the source of elevated p-tau in the blood in motor neuron diseases such as ALS. However, the authors don't provide concrete evidence that the source of elevated p-tau in the serum of ALS is derived from peripheral tau. Some of the mass spectrometric experiments to establish the peripheral tau in muscles and brain tissue also need further clarification as detailed below.
1. While the BD-tau assay on the commercial platform captures the brain derived small tau isoforms, the authors need to define more lucidly what is the pre-analytical BD-tau IP. Are they enriching for BD-tau using the antibody and then performing Simoa or are they preclearing the BD-tau? The process needs clarification as readers who are not familiar with immunoprecipitation process would think this preanalytical process is meant to remove the BD-tau from the serum.
2. Page 7-8: the sections describing the pre-analytical results compared to direct measurements are very confusing to understand. The authors have also performed p-tau 217 analysis in both the methods and the description of the data is not clearly written. There is semi-automated format and manual BD-tau IP, that is not defined in the method section.
3. Page 8: The preanalytical process was done in a smaller subset compared to direct Simoa measurements. Any particular reason why? Was sample availability the reason for the choice.
4. Page 9: Mass spec measurements analysis of BD-tau and big tau in brain and muscle samples are not properly defined and there are many caveats that need to be corrected.

a. While serum samples were tested with tau IP, searching through the supplementary methods shows no enrichment strategies were employed for these tissue samples. Total brain or muscle homogenates (10 µg protein) was digested with enzymes and specific tau peptides for small tau and big tau were targeted on the mass spec. The authors claim they found no BD-tau peptide in the muscle biopsies from ALS and disease controls (n=5/5), only observing one big tau peptide form exon 4a in these muscle tissues. The authors found both big tau and BD-tau specific peptide in the brains, which is quite different to what has been published in the literature about big tau. The authors do not provide evidence of other tau peptides in their measurements (N-terminus, mid-domain, microtubule binding region), making it hard to understand the total tau profile in brain and muscles.
b. The choice of peptides for the mass spec is very puzzling. Using Asp-N and Lys-C they derive the DEAAGHVTQARMVSK that is exon 4- exon 5 bridging peptide and is specific for small tau. They claim repeatedly trypsin cleavage peptides does not cover the exon 4-5 bridging region that is technically not correct. Using AspN and LysC combination reduces the N-terminal inserts diversity (0N, 1N and 2N isoforms) and provides only one unique peptide that the authors have targeted using their PRM mass spec method. AspN combined with trypsin (DEAAGHVTQAR) would further validate their findings for small tau in the muscle tissue.
c. Why monitor only one peptide from big tau? The exon 4a is large (255 amino acid) and the authors should monitor more than one peptide to ensure they have covered the big tau.
d. The concentration of tau protein in the periphery is 3-4 orders of magnitude lower compared to brain (Dugger, Brittany N. et al JAD, 2016, Hamsafar Y et al Neurosci. Letters 2023) The authors claim that they do not find BD-tau specific peptide in the muscles compared to big tau (exon 4a) peptide should be validated using tau specific enrichment, as low abundance of tau protein in complex protein digest from peripheral tissue such as muscle may have hindered sufficient signal to noise for BD-tau peptide detection. Using any commercial antibody (Tau-1, HT7, etc.) should be able to enrich tau in these samples and ratio of BD-tau/big tau signals would be more confident unlike what is currently presented.
e. Why only muscle and brain tissues, ALS impact upper and lower motor neurons. Is it possible for the authors to test any other tissues (nerves) to demonstrate the changes they observe in BD-tau and big tau are also present in these ALS affected tissues?
f. For the quantitation the authors need to use common tau peptides to normalize their PRM intensities. In Figure 4 as well as in the discussion Page 13 the inference of increased levels of BD-tau in C9ORF72-associated ALS could be strengthened if they observed both increased total tau and BD-tau. Normalization with total tau would further indicate BD-tau expression is increased in these patients.
5. In the page 14 the authors state that this paper clarifies the distinct biological origins of BD-tau and big tau across different tissues and disease states. However, the sample numbers are low per group to make any conclusions. Furthermore, while BD-tau IP in serum seems to indicate the source of elevated p-tau 217 and BD-tau in AD, however the authors have not performed the experiments to show that big tau and phosphorylated big tau could be the potential source in ALS serum. The plasma BD-tau and its consequent increase in prodromal AD and clinical AD compared to controls without amyloid positivity has been well established (Gonzalez-ortiz, F. et al Brain 2022; Gonzalez-ortiz, F. et al Nature Comm 2024, Montoliu-Gaya, Laia et al Nature Aging 2025), however the source of p-tau 217 elevation using commercial immunoassays in ALS need explanation – is it derived from small tau within the CNS or is it derived from the periphery (muscles) that are primarily made up of big tau? This has to be addressed by the authors in this manuscript before this can be considered for publication in Nature Communication.

Reviewer #4

(Remarks to the Author)
Review Comments: Abu-Rumeileh et al., “Selective detection of tau isoforms across brain, muscle and blood in Alzheimer´s disease and amyotrophic lateral sclerosis”

In this study “Selective detection of tau isoforms across brain, muscle and blood in Alzheimer´s disease and amyotrophic lateral sclerosis”, Abu-Rumeileh et al. examined the diagnostic utility of BD-tau specific immunoassays for improving specificity of tau-based biomarkers for detection of central (i.e AD) vs peripheral neurodegenerative processes (i.e. ALS). First, they measured serum and CSF brain-derived tau (BD-tau) in ALS (n=139), AD (n=101), and disease controls(n=75) using commercial Simoa immunoassay and found that serum BD-tau was increased in AD> ALS> disease controls whereas CSF BD-tau was increased in AD compared to ALS and disease controls with no difference between the latter two groups. Contrarily, CSF total tau was increased in AD and ALS compared to disease controls. Second, using a “two-step” approach involving an initial BD-tau IP to enrich for brain-derived species followed by immunoassay to serum BD-tau and ptau-217. Whereas pre-IP, serum BD-tau and ptau-217 were increased in AD and ALS compared to disease controls, post-IP, both BD-tau and ptau-217 were only increased in AD suggesting that enrichment of BD-tau increases sensitivity for AD. Finally, the authors examine peptides representative of BD-tau and peripheral tau (big tau) using mass spec in frontal cortex and muscle tissue from AD, ALS, and controls. In brain, relative levels of BD- and big tau were elevated in AD and a subset of ALS while in muscle, mild increases in big tau were detected in ALS and a disease control.

This study addresses an important question related to how central and peripheral sources of tau contribute to biofluid measures and begins to determine how analytic strategies can be optimized to improve specificity of tau measures for AD.

The strength of the study includes 1) the application of an established BD-tau assay towards a large cohort of ALS (n= 139) and AD (n= 101), 2) stratification of ALS cases based on AD co-pathology, and 3) the use of orthogonal approaches for

detecting BD-tau (i.e immunoassay, BD-tau IP enrichment, mass spec). However, the cohort characterization could be improved to provide more clinical insight into disease controls and the authors don't provide concrete evidence that the source of elevated p-tau in the serum of ALS is derived from peripheral tau. Some of the mass spectrometric experiments to establish the peripheral tau in muscles and brain tissue also need further clarification as detailed below.

Major points

1. The authors find that serum BD-tau is increased in ALS compared to disease controls after accounting for AD co-pathology. As tau may derive from brain vs peripheral sources and ALS is a disease defined by both upper and lower motor neuron pathology, this work could be strengthened by providing more phenotypic data related to ALS cases, particularly cognitive involvement and UMN vs LMN burden to determine whether BD-tau is in ALS is associated with cognitive/ UMN involvement. The disease control cohort should also be better characterized with regard to underlying diagnoses, particularly neurological diagnoses. It is indicated that disease controls were screened clinically, neuroradiologically and by CSF analyses but these criteria should be more clearly defined. For instance, non-neurodegenerative conditions such as multiple sclerosis, traumatic brain injury can predispose to neuronal injury and tau leak. In addition, it would be helpful to understand whether this group represents subsets that may serve as AD- or ALS mimic conditions.
2. The study utilizes postmortem frontal cortex tissue from AD (n=4), ALS (n=7, 3 C9, 4 sALS) and disease control (n= 5) for mass spec of small tau and big tau species. However, the postmortem intervals for a number of the tissues are quite prolonged > 48 hours which applies to 2 AD, 3 ALS, 3 disease controls. Since cytoskeletal proteins (i.e. tubulin, neurofilament) are known to have reduced staining intensity by 48 hr and lose signal by immunoblot even earlier < 24hr, this caveat should be highlighted in the data. Although sample size is too low to stratify based on PMI, the authors could consider designating samples with long PMI >48hr by asterisk or other indicator.
3. Given the differences in age across cohorts, the BD-tau data was adjusted for age but it is unclear why sex adjustment was not also made. The authors should show data suggesting no effect of sex on biomarker levels though their prior study showed sex affected ptau-181 and ptau-217 measures.
4. The serum BD-tau measures were performed on a larger cohort of ALS (n= 139), AD (n= 101), and disease controls (n=75). However, CSF analysis was only performed on a subset of samples available. The authors should comment on how the subset was chosen as well as rationale for using smaller cohort.
5. While the BD-tau assay on the commercial platform captures the brain derived small tau isoforms, the authors need to define more clearly the purpose of the pre-analytical BD-tau IP. Are they enriching for BD-tau using the antibody and then performing Simoa or are they preclearing the BD-tau? The process needs clarification as readers who are not familiar with immunoprecipitation process would think this preanalytical process is meant to remove the BD-tau from the serum. On pages 7-8, the authors performed p-tau 217 analysis using Simoa only and BD-tau IP methods but the description of the data is not clearly written. The difference between the semi-automated IP format and manual BD-tau IP should also be better defined in the methods section. In addition, the preanalytical process was done in a smaller cohort subset compared to direct Simoa measurements. Any particular reason why? Was sample availability the reason for the choice?
6. Page 9: Mass spec analysis of BD-tau and big tau in brain and muscle samples are not properly defined and multiple aspects of the data has to be explained as described below.
a. While serum samples were tested with BD-tau IP, searching through the supplementary methods shows no enrichment strategies were employed for these tissue samples. 10 µg of total protein from brain or muscle homogenates were digested with enzymes and specific tau peptides for small tau and big tau were targeted on the mass spec. The authors claim they found no BD-tau peptide in the muscle biopsies from ALS (n=5) and disease controls (n=5), only observing one big tau peptide form exon 4a in these muscle tissues. The authors also found both big tau and BD-tau specific peptide in the brains. Without an enrichment step, it would be difficult to conclude absence of BD-tau in muscle. The concentration of tau protein in the periphery is 1000-fold lower compared to cortical brain tissues (PMID: 26890756; PMID: 37330193, PMID: 37122097). The claim that BD-tau specific peptide is absent in muscle compared to big tau (exon 4a) peptide should be validated using tau specific enrichment. Using any tau specific antibody (HT7 that binds to mid-domain of tau isoforms) should be able to enrich tau in these samples and ratio of BD-tau/big tau signals would be more confident unlike what is currently being presented in Figure 4.
b. The authors do not provide evidence of other tau peptides in their measurements (N-terminus or proline rich region of tau) to help assess proportion of small tau vs big tau in these tissues. Does the muscle tau contain 3R and 4R (microtubule binding region) repeats?
c. The choice of peptides for the mass spectrometry analysis seems limited. Using Asp-N and Lys-C they derive the DEAAGHVTQARMVSK that is exon 4- exon 5 bridging peptide and is specific for small tau. However, they only monitor and target single peptide from the exon 4a of big tau. The exon 4a is quite large and the authors should monitor more than one peptide to ensure they have covered the big tau sequence. In the discussion the authors cite Koppisetti, R. et. al biorxiv (2025) that mentions another big tau isoform that they call exon 4a-long and distinct from exon 4a. Do the authors find any peptides from exon 4a-long in any of their tissue samples?
d. ALS impacts upper and lower motor neurons. Is it possible for the authors to test spinal cords and nerves to demonstrate the changes they observe in BD-tau and big tau are also present in these ALS affected tissues. Although muscle may be a source for big tau in the periphery, nerve should also be explored.
e. The authors need to use common tau peptides to normalize their target mass spec peptide intensities that will strengthen some of their claims that indicate BD-tau expression is increased in these ALS patients.
7. The authors in the discussion claim this paper clarifies the distinct biological origins of BD-tau and big tau across different tissues and disease states. However, the sample numbers are too low per group to make any conclusions. Furthermore, BD-tau IP in serum seems to indicate the source of elevated p-tau 217 and BD-tau in AD is centrally derived. The plasma BD-tau increase in late stages of AD compared to controls has been well established (Gonzalez-ortiz, F. et al Brain 2022; Montoliu-Gaya, Laia et al Nature Aging 2025). However, the source p-tau 217 elevation using commercial immunoassays in ALS need explanation – is it derived from small tau from brain or other nerves within the CNS or is it derived from the

periphery (muscles) that are primarily made up of big tau (PNS tau)? This has to be addressed by the authors with experimental evidence to clarify the source of big tau and phosphorylated big tau in ALS serum.by the authors in this manuscript.

Version 1:

Reviewer comments:

Reviewer #1

(Remarks to the Author)
The authors adequately addressed my comments.

Reviewer #2

(Remarks to the Author)
I co-reviewed this manuscript with one of the reviewers who provided the listed reports. This is part of the Nature Communications initiative to facilitate training in peer review and to provide appropriate recognition for Early Career Researchers who co-review manuscripts.

Reviewer #3

(Remarks to the Author)
Review Comments: Abu-Rumeileh et al., "Tau isoforms mapping across tissues and biofluids reveals a signature distinguishing Alzheimer´s disease from amyotrophic lateral sclerosis"

The authors of the study have now thoroughly addressed most of the points raised by the reviewer and have provided details regarding their approach to measure BD-tau and BD-p-tau217 in the tissues investigated. Especially the mass spectrometry results are very convincing, and details provided for all the workflows will be very useful for future validations and reproducibility. The authors clearly demonstrate that in AD brain derived tau and the corresponding brain derived p-tau217 is highly specific for changes associated with AD. Monitoring BD-p-tau217 not only increases the sensitivity to detect changes due to AD pathology, but is no more influenced by the peripheral tau proteoform contribution in other degenerative diseases such as amyotrophic lateral sclerosis (ALS) that may involve different disease mechanism beyond amyloid-beta driven tau pathology. They also provide evidence for diverse tau isoforms in the periphery (big tau) such as muscle biopsies and spinal cord tissues that are in line with current literature.

This study addresses an important question related to how central and peripheral sources of tau contribute to biofluid measures and begins to determine how analytic strategies can be optimized to improve specificity of tau measures for AD. The reviewer feels the manuscript could benefit from few things:
1. New BD-tau and BD-p-tau217 assays from NULISA and other vendors are also becoming available. Could they be also useful and have an advantage when it comes to high-throughput for measuring these biomarkers.
2. While the authors do highlight that peripheral tau and big p-tau contribution could be the reason for the serum tau increase in ALS independent of AD-pathology, do they predict ALS patients would benefit from the development of such assay and diagnose pathological associated with lower motor neurons.
3. The mass spec data is now very convincing and much improved, however the z-scored heatmaps do not do justice to the distribution of tau isoforms across the different tissues. Scatter plots of the ratios of BD-tau and big tau compared to total tau for all the tissues investigated would be better as they provide how these two tau isoforms change from CNS to PNS. P-tau217 IP results are interesting and highlights the peripheral tissues indeed have big tau with minimal contribution from exon4-5 specific tau isoform (small tau/BD-tau).
4. In light of the pan-tau IP from the muscles tissues and the author noticing exon 4a-L and exon 4a-S tau isoforms, it would be more appropriate for the authors to change the tau schematic to include both the big tau isoforms (exon 4a-L and exon 4a-S) in figure 3. The untargeted pan-tau IP proteomics results are interesting as they provide evidence for exon 4a-S isoforms. What is the relative contribution of the exon 4a-L and exon 4a-S in the muscles?

Reviewer #4

(Remarks to the Author)
The authors have substantially addressed all major initial concerns raised in the prior review. In particular, they have clarified the characteristics of their disease control cohort with defined inclusion criteria as well as provided correlations between ALS cognitive and UMN/LMN phenotype to serum BD-tau which support their interpretation of BD-tau. The restructuring of the Methods to distinguish and clarify direct Simoa, semi-automated IP, and manual IP workflows. The mass spec work has also been improved by adding tau enrichment to enable detection of BD-tau for normalization, inclusion of additional big-tau peptides, including a subset of paired spinal cord to analyses, and identified postmortem samples with prolonged PMI to inform interpretation.

Version 2:

Reviewer comments:

Reviewer #3

(Remarks to the Author)
The authors have now adequately addressed all the comments and now is ready to be published in Nature Communications

Open Access This Peer Review File is licensed under a Creative Commons Attribution 4.0 International License, which permits use, sharing, adaptation, distribution and reproduction in any medium or format, as long as you give appropriate credit to the original author(s) and the source, provide a link to the Creative Commons license, and indicate if changes were made.
In cases where reviewers are anonymous, credit should be given to 'Anonymous Referee' and the source.
The images or other third party material in this Peer Review File are included in the article's Creative Commons license, unless indicated otherwise in a credit line to the material. If material is not included in the article's Creative Commons license and your intended use is not permitted by statutory regulation or exceeds the permitted use, you will need to obtain permission directly from the copyright holder.
To view a copy of this license, visit https://creativecommons.org/licenses/by/4.0/

RESPONSE TO REVIEWERS' COMMENTS

Reviewer #1 (Remarks to the Author):

This manuscript addresses a clinically important issue in the field of blood-based biomarkers for neurodegenerative diseases, namely the biological and analytical specificity of circulating phosphorylated tau species. By combining ultrasensitive immunoassays, a selective brain-derived tau immunoprecipitation strategy, and targeted mass spectrometry across multiple tissue types, the authors provide a compelling and mechanistically grounded investigation of the peripheral contribution to blood tau measurements, particularly in amyotrophic lateral sclerosis.
The study is well designed, with multicentre cohorts, and builds coherently on the authors' previous work. Importantly, it delivers a clear message with direct implications for the interpretation and clinical deployment of plasma tau biomarkers in Alzheimer's disease, especially in populations with concurrent or suspected neuromuscular disorder. Although the manuscript is certainly important to the field, addressing the following comment would improve its clarity and transparency, for clinical implementation in selective situations.

Major Comments:
- The authors conclude that the BD-Tau-IP enables selective measurement of brain-derived tau species. However, as acknowledged in the Methods section, the absolute recovery of BD-tau and BD-p-tau217 after immunoprecipitation is unknown, and biomarker concentrations are therefore reported as relative values. It would strengthen the manuscript to discuss more explicitly:

We thank the reviewer for this important comment. We agree that these aspects are critical for the future translation of BD-Tau-IP–based measurements into routine clinical practice.

1) The reproducibility of the BD-Tau-IP procedure (both on AD and control subjects)

The analytical reproducibility of the BD-Tau-IP two-step approach was assessed at two levels: the pre-analytical BD-Tau-IP procedure and the downstream Simoa measurement platform.
The analytical reproducibility of the manual and semi-automated BD-Tau-IP protocols was assessed using serum pool samples. For the manual BD-Tau-IP, intra-assay CV was 7.2 % and inter-assay CV was 9.5 %. For the semi-automated BD-Tau-IP, intra-assay CV was 6.8 % and inter-assay CV was 8.7 %.
The analytical reproducibility of the Simoa assays was also assessed. For both BD-tau and p-tau 217 assays, intra-assay CV was <10% and inter-assay CV was <15%, indicating robust analytical performance of the measurement platform.
Inter-method reproducibility between the manual and semi-automated BD-Tau-IP procedures was assessed by Spearman correlation and Bland-Altman analysis in the 98 subjects who underwent both procedures. Strong rank-order correlation between the two procedures was observed (Spearman's r = 0.877, p<0.001), supporting the consistency of relative biomarker distributions across both BD-Tau-IP approaches. Bland-Altman analysis revealed an acceptable inter-method agreement with a mean difference (manual minus semi-automated) of +0.775 relative units (SD 0.889; 95% LoA: -0.967 to +2.518) and with 92.9% of measurements falling within the limits of agreement. Agreement was better in ALS (mean diff: +0.335, LoA: -0.407 to +1.077) and controls (mean diff: +0.265, LoA: -0.038 to +0.567) than in AD subjects (mean diff: +1.467, LoA: -0.520 to +3.453), likely attributable to the higher absolute BD-p-tau217 values in the AD group, which amplify absolute differences between methods even when relative agreement is maintained (a known feature of Bland-Altman analysis in datasets with wide dynamic ranges).
Taken together, these findings indicate acceptable inter-method reproducibility, with a systematic tendency for the manual IP to yield higher values compared to the semi-automated procedure, consistent with known procedural differences in sample volume and dilution factors between the two methods.
Importantly, despite the higher absolute differences observed in AD subjects, the diagnostic separation between AD and the other groups (ALS and controls) was robustly maintained with both manual and semi-automated BD-Tau-IP procedures, confirming that inter-method variability does not compromise the clinical utility of the two-step approach.

Taken together, although absolute recovery of BD-tau species after BD-Tau-IP cannot currently be determined, the consistent diagnostic group separation, the acceptable inter-method reproducibility, and the robust analytical performance of the downstream Simoa assays collectively support the reliability of the relative measurements reported in this manuscript and their validity for the diagnostic comparisons presented.

Reproducibility data are reported in the Result section (page 8) and in Supplementary Information (pages 5-6) and are discussed in the discussion (page 13).

2) If available, the potential batch-to-batch variability of antibody-functionalized magnetic beads
Concerning potential batch-to-batch variability of antibody-functionalized magnetic beads, all BD-Tau-IP experiments in the present study were performed using the same antibody clone 2B8 and bead preparation batch 0325-01, thus excluding batch-to-batch variability as a source of analytical variability in the current dataset. We acknowledge, however, that systematic evaluation of batch-to-batch variability will be an important step toward future standardization and clinical implementation of the BD-Tau-IP procedure.

We added these data in the supplementary information (page 8), and we discussed the topic in the discussion (page 13).

3) The possibility of developing quantitative mass spectrometry measurements (e.g., using AQUA peptides or other strategies). Given the current emphasis in the field on assay harmonization and standardization of blood-based tau biomarkers, as well as the importance of quantitation for longitudival monitoring, this point will be particularly relevant for translation into routine practice.
We agree that absolute quantification approaches based on targeted mass spectrometry, such as the use of isotopically labeled AQUA peptides or similar strategies, represent a promising step to enable fully quantitative measurement of BD-tau and BD-p-tau217 in biofluids.
All these points have been clarified as a limitation of the study in the revised Discussion section (page 15).

- The authors report that serum BD-tau measured with the one-step Simoa assay correlates with disease severity and progression in ALS, whereas these associations disappear after BD-Tau-IP. This observation strongly suggests that the initial correlations may reflect peripheral muscle-derived tau rather than central neurodegeneration. Expanding the discussion on this point would be valuable, particularly in comparison with neurofilament light chain (NfL), which is more firmly established as a marker of axonal damage.

We thank the reviewer for raising this important point. We agree that this observation deserves further discussion, particularly in comparison with neurofilament light chain (NfL), which is widely considered a robust marker of neuroaxonal damage in ALS.
Several lines of evidence support NfL as the most well-established blood and CSF biomarker in ALS, playing an important role in differential diagnosis against ALS mimics, as well as in tracking disease severity, prognostic assessment, and therapy monitoring (Halbgebauer 2022, Abu Rumeileh 2020, Verde 2021, Khalil 2024). In our study, serum BD-tau measured using the direct Simoa assay was associated with clinical severity and disease progression in ALS, whereas these associations disappeared after selective BD-Tau-IP enrichment. This finding suggests that the correlations observed with the direct assay may partly reflect peripheral tau release from degenerating muscle fibres rather than central neuroaxonal degeneration, as more specifically tracked by NfL. Indeed, the BD-tau antibody used in the IP procedure selectively captures BD-tau isoforms, thereby excluding peripheral tau species derived from skeletal muscle. This selectivity likely explains why the correlations with clinical severity markers observed with the direct Simoa assay are attenuated following BD-Tau-IP enrichment. Taken together, these findings highlight an important methodological advantage of the BD-Tau-IP approach over the direct Simoa assay in the context of ALS, where peripheral muscle degeneration may confound the interpretation of serum tau measurements.
Therefore, our findings suggest that serum BD-tau after BD-Tau-IP and NfL may provide complementary rather than redundant biological information.
We have expanded the Discussion to address this point (pages 14-15).

- A major strength of the study is the use of targeted mass spectrometry to distinguish BD-tau from big tau in muscle tissue. However, no mass spectrometric validation is provided for serum or CSF samples, where the immunoassays are ultimately applied. While the technical challenges are acknowledged, this remains an important limitation. A forward-looking discussion on the feasibility of mass spectrometry–based validation of BD-tau or BD-p-tau217 in blood would further enhance the methodological rigor of the manuscript.

We agree with the reviewer. In this study, mass spectrometry was applied to brain, spinal cord and muscle tissues to confirm the differential tissue distribution of BD-tau and big tau isoforms. Although the convergent tissue-specific mass spectrometry and biomarker findings strongly support a peripheral origin of the serum p-tau217 signal in ALS, direct proteomic confirmation of circulating BD-p-tau217 and big p-tau217 species in serum remains an important objective for future studies. Indeed, a limitation of the present study is the absence of mass spectrometric validation of BD-tau or BD-p-tau217 species in serum samples. Such analyses remain technically challenging due to the very low abundance of tau peptides in blood and the complexity and dynamic range of the blood proteome. Future developments in targeted proteomics workflows, including improved immunoenrichment strategies in combination with high-sensitivity mass spectrometric detection methods, may enable direct validation of BD-tau and big tau species in biofluids. We discussed this topic as a limitation in the discussion (pages 14 and 15).

- While the BD-Tau immunoprecipitation (BD-Tau-IP) represents a central methodological innovation of this study, the level of procedural detail currently provided is insufficient to ensure full reproducibility by independent laboratories.

In particular, the manuscript would benefit from a clearer and more standardized description of:

1) the starting serum volume used for each IP,

2) the amount of antibody and magnetic beads applied per sample,

3) the final elution volume,

4) the resulting concentration or dilution factor relative to native serum,

5) the handling and storage conditions of IP eluates prior to Simoa analysis.

This information is especially important given the observed differences between manual and semi-automated IP procedures and the authors' conclusion that further standardization is required before clinical implementation. Providing these details, either in the main text or in the Supplementary Methods, would substantially enhance methodological transparency and facilitate external validation.

We thank the reviewer for this important comment regarding methodological transparency. Most of the procedural details of the BD-Tau immunoprecipitation protocol have been already provided in the previous version of Supplementary Methods. These details are now clearly summarized in the Supplementary Methods (pages 8-10) as well as in the supplementary table 12, which compare the three protocols, to facilitate replication by independent laboratories.

1. the starting serum volume used for each IP protocol: 2x500 µL aliquots for each patient in the first manual IP, 200 µL for each patient in the second manual IP, and 2x400 µL aliquots for each patient in the semi-automated IP).
2. the amount of antibody per used aliquot of patient sample was 10 µg for the first manual IP and for the semiautomated IP, and 5 µg for the second manual IP. The volume of antibody-functionalized magnetic beads used per patient was 50 µL in the first manual IP and in the semiautomated IP, and 25 µL in the second manual IP.
3. the elution volumes: 2x50 µL for each patient in the manual IP, 30 µL for each in patient in the second manual IP, 2x75 µL for each patient in the semiautomated IP.
4. Resulting concentration factor relative to native serum: Taking into account the input serum volume, the elution volume, and the subsequent dilution step prior to Simoa analysis, the net concentration factors relative to native serum were approximately 2.5× for BD-tau and 3.3× for p-tau217 in the first manual IP, approximately 1.3× for p-tau217 in the second manual IP, and approximately 1.3× for BD-tau and 1.8× for p-tau217 in the semi-automated IP. These values represent theoretical estimates based on volumetric calculations; the true enrichment factors may differ due to incomplete recovery during the IP procedure, which remains unknown at this stage, as acknowledged in the manuscript.
5. The handling and storage conditions of IP eluates prior to Simoa analysis: In the manual protocol, all pipetting, washing, and bead handling steps were performed manually by the operator. In the semi-automated protocol, these steps were performed using a CyBio FeliX liquid handling instrument (Analytik Jena, Germany), with only the transfer of the final eluates into storage tubes performed manually.
   In the first manual and semi-automated IP protocols, the two eluate aliquots obtained per patient sample were transferred into 2 mL screw cap micro tubes (Sarstedt, Germany), mixed by vortexing, and stored at −80°C until Simoa analysis. In the second manual IP protocol, eluates were transferred individually into 2 mL screw cap micro tubes (Sarstedt, Germany) without pooling and stored at −80°C until Simoa analysis. In all protocols, eluates were measured after a single freeze-thaw cycle.

- Clinical positioning and proposed algorithm for the use of BD-p-tau217 measurement. While the analytical advantages of the BD-Tau-IP approach are convincingly demonstrated, the clinical added value of systematically applying this protocol in all diagnostic scenarios remains unclear. Given its increased pre-analytical complexity, cost, and limited scalability, it is unlikely that BD-p-tau217 measurement after immunoprecipitation would be justified as a universal first-line test.

To strengthen the translational relevance of the study, the authors are encouraged to propose a pragmatic clinical and/or analytical algorithm delineating the specific situations in which BD-p-tau217 assessment would be particularly informative.
Providing a decision framework—whether based on clinical features, biomarker ranges, or both—would help readers understand when and why the additional analytical effort of BD-Tau-IP is justified and would greatly facilitate implementation in both research settings and eventually future real-world diagnostic workflows.

We thank the reviewer for this constructive comment. We fully agree that specifying when the additional use of pre-analytical BD-Tau-IP to measure BD-p-tau217 is clinically justified would greatly strengthen the translational relevance of the study.
We have therefore added a dedicated paragraph to the Discussion section (pages 12-13) proposing a pragmatic two-level decision framework for BD-p-tau217 assessment, illustrated as a flow-chart (new Figure 5).
We propose the following step-wise approach:
First-line testing (direct Simoa p-tau217, one-step approach): Direct commercial Simoa measurement of blood p-tau217 should remain the first-line approach for all patients with suspected AD pathology. In patients with a clearly negative result, AD pathology is considered unlikely and no further tau testing is warranted. In patients with a clearly positive result in a clinically typical context, i.e., individuals with amnestic cognitive impairment and no evidence of concurrent conditions known to elevate peripheral tau species, the result is considered reliable and BD-Tau-IP is not required.
Second-line testing (BD-Tau-IP + Simoa p-tau217, two-step approach): BD-p-tau217 measurement after BD-Tau-IP should be reserved as a second-line confirmatory test in the following specific scenarios:
Scenario A. Known ALS, muscle disease, or systemic amyloidosis with already elevated blood p-tau217 on direct Simoa assay. This is the most clinically relevant scenario. In patients with a pre-existing diagnosis of ALS, a muscle disease, or systemic amyloidosis (AL or ATTR) who show elevated serum p-tau217 on direct Simoa, the critical clinical question is whether this elevation reflects peripheral p-tau217 release, expected in the context of the underlying disease, or rather indicates an additional AD co-pathology. In this context, it is also worth noting that a recent study reported elevated plasma p-tau181 in patients with myotonic dystrophy type 1 (DM1), a multisystem disorder affecting both skeletal muscle and the central nervous system (Nakamura et al., J Clin Med 2025). Although this observation specifically concerns p-tau181 rather than p-tau217, it is biologically plausible that the same phenomenon extends to other phosphorylated tau species. Regarding systemic amyloidosis, Kaeser et al. (Nat Med 2026) found elevated serum p-tau181 and p-tau 217 in patients with AL and ATTR amyloidosis, with biomarker increases in the same range as those observed in AD and with more pronounced elevations in the presence of polyneuropathy. The precise tissue source of elevated p-tau in this context has not been definitively established; the authors propose that cardiac muscle, renal tissue, and peripheral nerves, all of which may be affected by amyloid deposits in systemic amyloidosis, represent plausible sources of peripheral tau release into the circulation, in addition to or independently of any CNS involvement.
As demonstrated in our study, BD-Tau-IP selectively captures BD-tau, effectively removing the peripheral big tau signal. Accordingly, in the above-mentioned clinical scenario, a normal BD-p-tau217 after BD-Tau-IP would confirm that the elevation is of peripheral origin and represents a false positive, whereas elevated BD-p-tau217 concentrations would support the presence of genuine AD co-pathology. This distinction is of direct clinical and prognostic relevance, particularly in ALS, where true AD co-pathology is estimated to be present in approximately 10–15% of cases (Barba et al., J Alzheimers Dis 2023), and in elderly ATTR patients, who are at age-related risk for incipient AD pathology.
Scenario B. Blood p-tau217 elevated or borderline on direct Simoa with an atypical clinical presentation. In patients with an elevated or borderline p-tau217 result but an atypical presentation, such as young age, a non-amnestic cognitive syndrome, or a clinical-biomarker discordance (i.e. elevated p-tau 217 and negative amyloid-PET), the two-step approach including BD-Tau-IP allows discrimination between true CNS-derived elevation and a potential analytical false positive before diagnostic or therapeutic decisions are made.
Scenario C. New diagnosis of ALS, muscle disease, or systemic amyloidosis in whom AD screening is not yet performed and is planned. In these patients, direct Simoa measurement is likely to yield a false-positive result due to the release of peripherally derived p-tau217 species associated with the underlying disease. Accordingly, BD-p-tau217 should be used as the primary analytical approach for AD biomarker assessment in this population, rather than direct Simoa, thereby avoiding the need for a subsequent confirmatory step.
Scenario D. Clinical trials requiring high specificity for AD patient selection. In research settings where maximum specificity for AD biomarker-based enrolment is required, BD-p-tau217 after BD-Tau-IP represents a higher-specificity alternative that may reduce false-positive enrolment, particularly in older or multimorbid populations where subclinical muscle or nerve disease is common and frequently unrecognised.

This proposed algorithm is supported by the data presented in the current study, our previous work (Abu-Rumeileh et al., Nat Commun 2025), and recent independent evidence on non-AD conditions associated with elevated blood p-tau from peripheral origin (Kaeser et al., Nat Med 2026; Nakamura et al., J Clin Med 2025).

We acknowledge, as the reviewer correctly notes, that BD-Tau-IP is not suitable as a universal first-line test given its pre-analytical complexity, and limited current scalability. Its clinical value lies specifically in the scenarios outlined above, where the analytical performance of direct Simoa assays is likely to be influenced by peripheral tau sources. Further standardisation and cost-reduction strategies will be required before broader clinical implementation.

Minor comments :

- Please, use the more widely adopted « p-Tau217 » or « p-tau217 » terminology instead of p-tau 217

We decided to adopt p-tau217 and p-tau181.

- The terms BD-tau, small tau, and brain-derived tau are used interchangeably throughout the manuscript. Providing a clear and operational definition early in the Introduction would improve clarity.

We decided to specify in the introduction that BD-tau, brain-derived tau and small tau are the same tau peptide and then we used consistently throughout the manuscript the word "BD-tau".

Reviewer #2 (Remarks to the Author):

I co-reviewed this manuscript with one of the reviewers who provided the listed reports. This is part of the Nature Communications initiative to facilitate training in peer review and to provide appropriate recognition for Early Career Researchers who co-review manuscripts.

Reviewer #3 (Remarks to the Author):

The study "Selective detection of tau isoforms across brain, muscle and blood in Alzheimer´s disease and amyotrophic lateral sclerosis" by Samir Abu-Rumeileh et al. investigates the origin of the blood phosphorylated tau (p-tau) 217 in amyotrophic lateral sclerosis (ALS), that is an diagnostic biomarker for Alzheimer's disease (AD) and is also elevated in ALS patient plasma. They hypothesize the likely source of the increased p-tau217 originates from peripheral tau that are released from denervated muscle fibers.
To further prove their point, they measure a novel tau fragment in the serum using commercial immunoassay, brain derived tau (BD-tau) along with p-tau 217 in a cohort of ALS (n=139), AD (n=101) and disease controls (n=75). They also performed pre-analytical magnetic bead BD-tau immunoprecipitation (BD-tau IP). In their direct immunoassay, they found serum BD-tau and p-tau217 were elevated in both AD and ALS compared to controls. Whereas, following BD-tau-IP, serum BD-tau and p-tau 217 were observed exclusively in AD. The authors also proved the BD-tau and peripheral tau (big tau) species in brains and muscle samples by targeted mass spectrometry.
This study is interesting and aims at resolving the long-standing biomarker related question about the source of elevated p-tau in the blood in motor neuron diseases such as ALS. However, the authors don't provide concrete evidence that the source of elevated p-tau in the serum of ALS is derived from peripheral tau. Some of the mass spectrometric experiments to establish the peripheral tau in muscles and brain tissue also need further clarification as detailed below.

1. While the BD-tau assay on the commercial platform captures the brain derived small tau isoforms, the authors need to define more lucidly what is the pre-analytical BD-tau IP. Are they enriching for BD-tau using the antibody and then performing Simoa or are they preclearing the BD-tau? The process needs clarification as readers who are not familiar with immunoprecipitation process would think this preanalytical process is meant to remove the BD-tau from the serum.
We thank the reviewer for this important comment. We agree that our introduction may be misleading to readers unfamiliar with immunoprecipitation (IP), as it could be interpreted as a preclearing step aimed at removing BD-tau from the serum. Nevertheless, the BD-Tau-IP is a selective enrichment procedure, not a preclearing step. Briefly, the 2B8 antibody, which binds specifically to the exon 4-5 junction epitope present exclusively in BD-tau isoforms, is covalently coupled to magnetic beads. When incubated with serum samples, the functionalized beads selectively capture BD-tau protein from

the sample, while non-brain-derived tau species (high molecular weight peripheral tau isoforms or big tau), which lack the exon 4-5 epitope, remain in the supernatant and are discarded. The captured BD-tau is subsequently eluted from the beads by heat denaturation and the eluate, now enriched for BD-tau species, is used for quantification of BD-tau and p-tau217 on the Simoa platform. In other words, the BD-Tau-IP does not remove BD-tau from the sample, on the contrary, it selectively concentrates BD-tau isoforms while eliminating interfering peripheral tau species, thereby improving the specificity of the downstream Simoa measurement.
Notably, our results indicate that the commercial BD-tau Simoa assay alone does not fully discriminate BD-tau from peripheral big tau in serum, which underscores why the pre-analytical IP enrichment step is necessary to achieve isoform-specific BD-tau quantification. This distinction has been clarified in the revised introduction (pages 4-5) and Methods section (pages 17-18).

2. Page 7-8: the sections describing the pre-analytical results compared to direct measurements are very confusing to understand. The authors have also performed p-tau 217 analysis in both the methods and the description of the data is not clearly written. There is semi-automated format and manual BD-tau IP, that is not defined in the method section.

We thank the reviewers for this important observation. We acknowledge that the presentation of the "two-step approach" methods and results was not sufficiently clear in the original manuscript, and we have made the following substantive changes to address this concern.

1) In the Results section, we have reorganised the description of serum BD-tau and p-tau217 findings into four clearly labelled and logically ordered subsections:

- A first subsection dedicated (pages 5-7) to direct one-step Simoa measurements (serum BD-tau, serum p-tau217, CSF BD-tau, and CSF t-tau), in which results for all biomarkers measured by direct immunoassay are now presented together and clearly labelled as 'one-step approach' throughout. We also added a brief concluding sentence explicitly stating that direct Simoa measurement of both serum BD-tau and p-tau217 does not discriminate ALS from disease controls or AD from ALS with high accuracy, to set the stage for the two-step approach results.
- A second subsection (page 7) dedicated to the two-step approach including semi-automated BD-Tau-IP, in which results for both BD-tau and BD-p-tau217 after BD-Tau-IP are now explicitly presented in parallel, highlighting that, in contrast to direct measurements, serum BD-tau and BD-p-tau217 elevations were observed exclusively in the AD group after BD-Tau-IP, with no significant differences between ALS and disease controls for either biomarker.
- A third subsection (page 8) on comparative diagnostic accuracy (one-step vs. two-step approach with semi-automated BD-Tau-IP), now presented as a dedicated paragraph with separate reporting of ROC curve results for blood BD-tau and p-tau217 across all three diagnostic comparisons, to allow direct comparison of the two analytical approaches.
- A fourth subsection (pages 8-9) on the two-step approach with manual BD-Tau-IP, in which the main findings of the two manual runs are briefly summarised, and readers are referred to the Supplementary Results for full details, avoiding redundancy in the main text. Moreover, here we discussed the inter-method reproducibility of the two-step approach.

2) In the Methods section (pages 17-18) and supplementary methods (pages 8-10) sections we have added dedicated paragraphs clearly defining the key differences between the manual and semi-automated BD-Tau-IP protocols. The rationale for presenting the results of the semi-automated protocol in the main text is now explicitly stated: we analysed the larger and more representative sample across cohorts by means of the two-step approach including semi-automated BD-Tau-IP.
Both protocols used the same BD-tau antibody-functionalized magnetic beads and the same Simoa-based downstream quantification, but differed in starting serum volume, elution volume, and dilution factors applied prior to Simoa analysis. Also, the first and the second run of manual IP used different starting serum volume, elution volume, and dilution factors. Moreover, in the manual protocol, all pipetting and washing steps were performed by hand using standard laboratory equipment. In the semi-automated protocol, the same immunoprecipitation principle was applied, but the pipetting, washing, and bead handling steps were performed using a CyBio FeliX liquid handling instrument (Analytik Jena, Germany), enabling higher throughput and improved procedural standardization.
All the methodological details for both protocols are provided in the Supplementary Methods as well as in the new supplementary table 12.

3. Page 8: The preanalytical process was done in a smaller subset compared to direct Simoa measurements. Any particular reason why? Was sample availability the reason for the choice.

Sample availability was the primary reason underlying the smaller cohort size for the two-step approach analyses compared to direct Simoa measurements. Indeed, the two-step approach with BD-Tau-IP requires a relatively large starting serum volume per patient (up to 1,000 µL for the first manual IP and 800 µL for the semi-automated IP), which substantially limits the number of samples that can be processed. Moreover, in the same cohort, serum samples had already been used for direct Simoa measurements of multiple biomarkers (BD-tau, p-tau 181, p-tau 271, tau, NfL) for this study and our previous one (Abu-Rumeileh et al. Nature Communications 2025), which progressively reduced the available volume for subsequent BD-Tau-IP experiments. As a result, BD-Tau-IP procedures were performed in subsets of patients for whom sufficient residual serum volume was available. We acknowledge this as a limitation of the current study and have added a statement in the discussion of the revised manuscript (page 15).

4. Page 9: Mass spec measurements analysis of BD-tau and big tau in brain and muscle samples are not properly defined and there are many caveats that need to be corrected.

a. While serum samples were tested with tau IP, searching through the supplementary methods shows no enrichment strategies were employed for these tissue samples. Total brain or muscle homogenates (10 µg protein) was digested with enzymes and specific tau peptides for small tau and big tau were targeted on the mass spec. The authors claim they found no BD-tau peptide in the muscle biopsies from ALS and disease controls (n=5/5), only observing one big tau peptide form exon 4a in these muscle tissues.
The authors found both big tau and BD-tau specific peptide in the brains, which is quite different to what has been published in the literature about big tau.
The authors do not provide evidence of other tau peptides in their measurements (N-terminus, mid-domain, microtubule binding region), making it hard to understand the total tau profile in brain and muscles.

We thank the reviewer for this important methodological comment.
First, we respectfully disagree with the reviewer's interpretation that the co-presence of BD-tau and big tau peptides in brain tissue is inconsistent with the literature. In fact, this finding is fully supported by well-established literature. Indeed, it has been recognized that big tau is also expressed in specific CNS neuronal populations and CNS regions (Boyne 1995, Nothias 1995; Fischer & Baas, Trends Neurosci 2020; Fischer, Front Mol Neurosci 2022; Fischer & Baas, Front Mol Neurosci 2025). This interpretation is further corroborated by the very recent comprehensive immunoprecipitation-MS analysis of Koppisetti et al., who demonstrated that in human brain cortex, small tau (BD-tau) constitutes approximately >99% of total tau, while big tau accounts for the remaining ~0.3%. Importantly, Koppisetti et al. found no disease-specific differences in big tau content in brain tissue across AD, ALS and control subjects. This paper has been recently accepted for publication in Annals of Neurology (Koppisetti et al., Annals Neurol 2026, in press); the accepted version is currently available at https://www.biorxiv.org/content/10.1101/2025.09.15.676127v2.
The detection of a minor big tau fraction alongside the predominant BD-tau pool in human brain cortex is therefore not anomalous. The above-mentioned data are fully consistent with our own observations of both BD-tau and big tau peptides in brain tissue of AD, ALS and control cases.

Second, we acknowledge that our mass spectrometry analysis was intentionally focused on two specific tau species, BD-tau (small tau, with exon 4/5 junction peptide) and big tau (with exon 4a peptide), without profiling the full tau peptide spectrum including N-terminal, mid-domain, and microtubule binding region (MTBR) peptides. This was a deliberate choice reflecting the primary aim of our study, which is to develop and validate BD-tau and BD-p-tau217 as new biomarkers in AD in the comparison to ALS and especially to better understand our findings on serum using BD-Tau-IP, by characterizing the distribution of BD-tau and peripherally-derived big tau isoforms in clinically relevant biofluids and tissues. A comprehensive atlas of tau peptide distribution across tissues was beyond the scope of the present work.

Regarding the broader tau peptide profile across multiple tissues and diagnostic groups, we refer the reviewer to the study by Koppisetti et al., which performed an exhaustive IP-MS characterization of tau peptides, including N-terminal, mid-domain, MTBR, and exon 4a-L and exon 4a-S specific peptides, across multiple CNS and PNS tissues in ALS and control subjects and across brain tissue in AD, ALS and controls subjects (Table 2 of the paper Koppisetti et al.2026). Specifically, this paper demonstrated that common tau peptides spanning the N-terminus, proline-rich region, and MTBR are uniformly distributed across brain, spinal cord, and peripheral nerve, and serve as stable internal reference peptides for normalization.
In this paper big tau expression was higher in the spinal cord compared to brain, and further increased in sciatic nerve, confirming the central-to-peripheral gradient of big tau expression. The analysis by Koppisetti et al. focused on the regional distribution of tau isoforms across tissues rather than on disease-specific differences in peptide profiles.
Given the comprehensive characterization already provided by Koppisetti et al., we believe that replicating this full peptide profiling in our dataset would not substantially advance the specific aims of the present study.

However, we note that among the peptides monitored in our updated study, IATPRGAAPPGQK covers the proline-rich region (mid-domain) and serves as normalization reference, partially addressing the concern regarding mid-domain coverage. N-terminal peptides and MTBR peptides including 3R/4R-specific sequences were not targeted, as profiling these domains was beyond the scope of the present study. For this comprehensive characterization we refer the reviewer to Koppisetti et al. (Table 2 of the paper).

Nonetheless, in direct response to the requests of Reviewers 3 and 4, we have substantially strengthened our mass spectrometry data by performing pre-analytical immunoprecipitation-based tau enrichment prior to target mass spectrometric analysis, using a pan-tau antibody and a p-tau217-specific antibody, to provide a more robust and specific characterization of BD-tau (small tau) versus big tau species and their phosphorylated forms across different tissues.
Importantly, for big tau we monitored two independent peptides to ensure specificity of detection, as requested from the reviews. We also included two common tau peptides in our analysis. For further methodological details regarding the peptide monitoring, please refer to the point C below.
We have also extended the analysis to include spinal cord samples from ALS patients and controls, enabling a direct comparison of tau isoform profiles across brain, spinal cord, and skeletal muscle. For further details see answer to question 4, point E.
The full methodological details and results of this enrichment strategy are described in detail in the response to question 4, point D below, to which we refer the reviewer.
Detailed results from the p-tau217-IP followed by targeted mass spectromectric analysis in muscle samples are reported in answer 5 below.
Taken together, these new IP-MS data across brain, spinal cord, and muscle provide a coherent and biologically consistent picture of BD-tau and big tau isoform distribution that strongly supports the specificity of our BD-tau and BD-p-tau217 assays for BD-tau signals.
Our own observations are completely in agreement with the findings of Koppisetti et al. Notably, Koppisetti et al. cite our previous work (Abu-Rumeileh et al., Nat Commun 2025) as independent evidence for elevated p-tau in ALS muscle, further reinforcing the convergence of findings across the two studies.
We hope these additions fully address the reviewer's concerns, while maintaining the clinical and translational focus of the study on BD-tau biomarker development in AD and ALS.

b. The choice of peptides for the mass spec is very puzzling. Using Asp-N and Lys-C they derive the DEAAGHVTQARMVSK that is exon 4- exon 5 bridging peptide and is specific for small tau. They claim repeatedly trypsin cleavage peptides does not cover the exon 4-5 bridging region that is technically not correct.
We thank the reviewer for this important remark. We acknowledge that the wording was not correct, and other digestion methods can provide suitable fragments to cover the BD-tau specific exon 4 to 5 bridging region. The selection was based on in silico digestions and pre-experiments testing different enzymes and enzyme combinations. The combination of Asp-N and Lys-C showed the best results in terms of data completeness while yielding specific peptides for BD-tau, big tau and common peptide to all described tau isoforms. The peptide bridging the exon 4–5 junction, generated by trypsin digestion, has 38 amino acids (QAAAQPHTEIPEGTTAEEAG IGDTPSLEDEAAGHVTQAR) and a mass of 3955.85 Da, which is quite large and adversely affects reliability in our setup We have corrected the statement in the methods (page 20) and in the discussion (page 13) to reflect that there is no fixed technical constraint.

Using AspN and LysC combination reduces the N-terminal inserts diversity (0N, 1N and 2N isoforms) and provides only one unique peptide that the authors have targeted using their PRM mass spec method.
AspN combined with trypsin (DEAAGHVTQAR) would further validate their findings for small tau in the muscle tissue.
We thank the reviewer for this comment. The primary aim of this study was to develop and validate BD-tau and BD-p-tau217 as new biomarkers in AD in the comparison to ALS. To support this purpose, the targeted mass spectrometry method was designed to specifically discriminate BD-tau and big tau. Neither the discrimination between 0N, 1N and 2N isoforms nor between 3R and 4R isoforms was intended in this study.
Moreover, we would like to highlight that DEAAGHVTQAR is contained within our measured peptide DEAAGHVTQARMVSK, which spans the same exon 4-5 junction and provides equivalent isoform specificity.
The additional validation suggested by the reviewer would require a separate digestion with AspN and trypsin, doubling the starting material required, which is not feasible given the limited availability of muscle samples and the ~1mg total protein required for immunoprecipitation followed by targeted mass spectrometry approaches. Future studies with larger tissue availability could consider this additional validation approach in peripheral tissues.

c. Why monitor only one peptide from big tau? The exon 4a is large (255 amino acid) and the authors should monitor more than one peptide to ensure they have covered the big tau.

We thank the reviewer for the suggestions. We totally agree that data for the detection of big tau benefit from monitoring more than one peptide, especially for quantitative information. We have improved the targeted MS method (page 20) based on this comment to include a second peptide from exon 4a (DTEGGRHAPELLK), in addition to the previously monitored exon 4a peptide (VSTEIPASEPDGPSVGRAK), and two peptides common to all tau isoforms (LTFRENAK and IATPRGAAPPGQK) for normalization purposes.
The location of all monitored peptides within the BD-tau and big tau sequences is illustrated in the updated Figure 3A, which shows the exon 4a-specific peptides for big tau (yellow), the BD-tau-specific bridging peptide spanning the exon 4–5 junction (red), and the two common tau peptides (grey). Moreover, detailed data of the analysed peptides are reported in supplementary table 15.

d. The concentration of tau protein in the periphery is 3-4 orders of magnitude lower compared to brain (Dugger, Brittany N. et al JAD, 2016, Hamsafar Y et al Neurosci. Letters 2023). The authors claim that they do not find BD-tau specific peptide in the muscles compared to big tau (exon 4a) peptide should be validated using tau specific enrichment, as low abundance of tau protein in complex protein digest from peripheral tissue such as muscle may have hindered sufficient signal to noise for BD-tau peptide detection. Using any commercial antibody (Tau-1, HT7, etc.) should be able to enrich tau in these samples and ratio of BD-tau/big tau signals would be more confident unlike what is currently presented.

We thank the reviewer for this important methodological point. We agree that the low concentration of tau in peripheral tissues compared to brain tissue represents a significant analytical challenge, and that direct mass spectrometry analysis without prior tau enrichment may be insufficient to confidently detect and quantify BD-isoforms in muscle tissue. We therefore agree with the reviewer's suggestion to perform pre-analytical tau-specific enrichment prior to targeted MS analysis.
To address this, we performed immunoprecipitation-based tau enrichment prior to targeted mass spectrometry analysis on brain from AD, ALS and controls as well as spinal cord and muscle samples from ALS patients and controls.

The enrichment was performed using a new semi-automated IP protocol using functionalized magnetic beads conjugated to a pan-tau antibody. Pan-tau-IP was performed on total protein from brain (AD n=4, ALS n=7, disease controls n=5), spinal cord (ALS n=6, disease controls n=5) and muscle (ALS n=5, disease controls n=5) protein extracts. Given the expected markedly lower tau concentration in peripheral tissues (muscle) compared to central nervous system tissues (Koppisetti et al., 2026, Dugger, Brittany N. et al JAD, 2016, Hamsafar Y et al Neurosci. Letters 2023), the amount of total protein used as starting material for pan-tau IP was adjusted accordingly based on published tau concentration estimates across tissues (Koppisetti et al., 2026, Dugger, Brittany N. et al JAD, 2016, Hamsafar Y et al Neurosci. Letters 2023). For the IP we used functionalized magnetic beads with pan-Tau antibody 7E5 directed to amino acids 156-165 (RGAAPPGQKGQA) of 2N4R Tau (catalogue number 847-0102006301, Roboscreen GmbH) as previously described (Morgado et al. 2025, 2025).
The eluates were subsequently subjected to targeted MS analysis for BD-tau and big tau following the digestion method as described in the previous version of the paper. Importantly, big tau was monitored using two independent peptides to ensure detection specificity, rather than a single peptide, and, in addition, two peptides common to all tau isoforms were included. For further methodological details please refer to the response to question 4, point C.

The results obtained following tau enrichment confirm and substantially extend our previous findings and those from Koppisetti et al. across three tissue compartments. Both BD-tau and big tau peptides were detected in all samples regardless of diagnosis and in all tissues. The highest BD-tau/t-tau ratios were detected in the frontal cortex, with BD-tau clearly predominating over big tau and a stable BD-tau/big tau ratio across diagnostic groups.
In spinal cord, an intermediate pattern was observed, with a trend toward to higher relative contribution of big tau compared to brain but with BD-tau remaining detectable, in keeping with the central-to-peripheral gradient described by Koppisetti et al.
In muscle, big tau was the dominant species in both ALS patients and controls, with minimal BD-tau signal. Indeed, big tau /t-tau ratio showed the highest values in muscle and the lowest in the frontal cortex.
To quantitatively characterize this isoform distribution across tissue compartments, we calculated the BD-tau/big tau ratios as suggested by the reviewer. These ratios demonstrate that the relative abundance of BD-tau to big tau decreases along the CNS- peripheral tissues (muscle) axis with a clear central-to-peripheral gradient, with brain and spinal cord both showing higher ratios with considerable overlap between them and muscle consistently showing the lowest ratio.
Raw intensities for all tau species indicate a trend toward increase of BD-tau, big tau and t-tau levels in the frontal cortex of AD patients compared to disease controls and ALS patients. Likewise, tau intensities tended to be higher in the spinal cord of ALS patients than in controls. Raw intensities for tau species in muscle showed no significant differences between ALS patients and disease controls for any of the measured tau species, consistent with big tau predominance in muscle reflecting a tissue-intrinsic biological property rather than a disease-specific phenomenon.

These findings on muscle tissue extends the gradient originally described by Koppisetti et al., who examined sciatic nerve as a peripheral tissue, to skeletal muscle, demonstrating that the central-to-peripheral shift from BD-tau predominance to big tau predominance is preserved at the level of skeletal muscle. These results confirm that BD-tau expression, while predominantly CNS-derived, extends across the neuroaxis and is detectable at minimal levels in peripheral muscle tissue.

Taken together, these data confirm that the original failure to detect BD-tau in muscle was a technical limitation, due to insufficient signal-to-noise as a consequence of the low abundance of tau in peripheral tissues (as correctly anticipated by the reviewer) and that BD-tau is indeed present in muscle, albeit at minimal levels relative to big tau. This strongly supports the conclusion that the BD-tau signal measured in serum using the two-step approach reflects primarily BD-tau and is not substantially confounded by peripheral muscle-derived tau.

We added the methods of the new pan-tau-IP followed by targeted mass spectrometry in methods (pages 19-21), supplementary methods (pages 11-13) and the respective results (pages 9-10) and discussion (pages 13-14) in the manuscript.
We also modified the figure 4 to display the results from the immunoprecipitation + MS experiments.
We added a new supplementary figure 3 to show tau raw intensity values.

e. Why only muscle and brain tissues, ALS impact upper and lower motor neurons. Is it possible for the authors to test any other tissues (nerves) to demonstrate the changes they observe in BD-tau and big tau are also present in these ALS affected tissues?

We thank the reviewer for this important comment. We fully agree that ALS affects both upper motor neurons (UMNs) in the primary motor cortex and lower motor neurons (LMNs) in the anterior horn of the spinal cord, and that ideally both tissue compartments should be investigated to characterize the distribution of BD-tau and big tau isoforms across ALS-affected neuronal populations.
Regarding peripheral nerve tissue, we acknowledge that nerve biopsies from ALS patients and disease controls were not available for our study. However, peripheral nerve involvement in ALS is generally considered secondary and less consistent compared to the motor neuron degeneration, and nerve biopsies are usually not part of the routine diagnostic workup in ALS.
To directly address the reviewer's concern regarding motor neuron involvement and also in accordance with the suggestions of reviewer 4, we have included spinal cord tissue for a subset of 6 ALS cases and 4 disease controls, of whom we included brain samples. We added these data in the method section (page 19) and in the supplementary table 13.

The spinal cord, in particular the ventral/anterior horn, contains the lower motor neurons that are primarily and consistently affected in ALS, and has been extensively used in neuropathological studies of ALS to characterize TDP-43 pathology, motor neuron loss (Brettschneider et al., Ann Neurol 2013; Saberi et al., Neurol Clin 2015) as well as mass spectrometry-based proteomic studies in ALS (Oeckl, et al. Acta Neuropathol 2020). Together, these studies establish the spinal cord as the most relevant and accessible post-mortem tissue for investigating lower motor neuron pathology in ALS.

For the specific MS analyses on spinal cord samples please refer to point D. Importantly, our data are in line with those of Koppisetti et al., showing that the spinal cord contains both BD-tau and big tau peptides. The BD-tau/big tau ratio in spinal cord was higher than in muscle and showed a trend toward a higher relative contribution of big tau compared to brain, with considerable overlap between spinal cord and brain values. This intermediate pattern makes the spinal cord an informative tissue for investigating the central-to-peripheral gradient of tau isoform distribution in the context of ALS pathology.

Regarding the brain samples, we would like to clarify that all ALS brain samples included in our study were derived from the frontal cortex, specifically including the primary motor cortex. This region harbours the upper motor neurons, whose degeneration is a defining neuropathological feature of ALS and has been characterised in detail in post-mortem studies (Brettschneider et al., Ann Neurol 2013; Saberi et al., Neurol Clin 2015). This has been already reported in the method section.
Thus, our combined analysis of frontal cortex and spinal cord tissue directly addresses UMN and LMN pathology in ALS, respectively, providing a comprehensive assessment of BD-tau and big tau distribution across both motor neuron populations affected in ALS.

However, we refer the reviewer to Koppisetti et al. (Annals Neurol 2026), who characterized BD-tau and big tau distribution in sciatic nerve, brachial plexus and dorsal root ganglion from ALS cases and controls, demonstrating that peripheral nerve tissues are enriched in big tau compared to CNS tissues, consistent with our findings in skeletal muscle. We discussed this point also in the discussion (pages 13-14).

f. For the quantitation the authors need to use common tau peptides to normalize their PRM intensities. In Figure 4 as well as in the discussion Page 13 the inference of increased levels of BD-tau in C9ORF72-associated ALS could be strengthened if they observed both increased total tau and BD-tau. Normalization with total tau would further indicate BD-tau expression is increased in these patients.
We thank the reviewer for this methodological suggestion. Regarding the normalization of PRM intensities using common tau peptides, we agree that this represents an important analytical refinement.
To address this point, we have added two common tau peptides to the PRM workflow (please refer to point 4C). These peptides were used to normalize PRM intensities for the BD-tau and big tau-specific peptides, enabling direct comparison of BD-tau/t-tau and big tau/t-tau ratios across tissues and diagnostic groups. Additionally, the BD-tau/big tau ratio was calculated to directly reflect the relative isoform composition within each sample.
We modified the methods (page 20), results (pages 9-10), the discussion (pages 13-14) and the figure 4 accordingly, including these ratios, as requested by Reviewers 3 and 4. Additionally the raw PRM tau intensities are provided in a supplementary figure 3.

Regarding the observation on C9ORF72-associated ALS samples, we wish to inform the reviewer that, following IP-based tau enrichment in the revised experiments, the previously reported apparent elevation of BD-tau in C9ORF72-associated ALS samples was not confirmed in the updated analysis. The updated Figure 4 therefore no longer reports this finding, and the corresponding statement in the Discussion has been removed.
We believe the original observation was likely influenced by technical limitations of direct digest MS in low-abundance peripheral tissue, and that the IP-enriched data are more reliable.

5. In the page 14 the authors state that this paper clarifies the distinct biological origins of BD-tau and big tau across different tissues and disease states. However, the sample numbers are low per group to make any conclusions.

We thank the reviewer for this important comment. We acknowledge that the number of post-mortem tissue samples analysed is limited, as is inherent to studies using human post-mortem brain tissue, where availability is constrained by ethical, logistical, and neuropathological quality requirements. Nevertheless, our sample size is consistent with prior MS-based studies investigating biomarker distribution in human post-mortem CNS and peripheral tissues (Oeckl, et al., 2020; Koppisetti et al., 2026). However, we fully acknowledge the reviewer's concern. Our tissue experiments were designed as a mechanistic validation study, primarily aimed at providing direct biological evidence for the tissue-level origin of the biomarker alterations observed in serum and CSF.
We recognize that the limited sample size precludes definitive conclusions regarding tissue-specific tau isoform distribution, and we have explicitly acknowledged this as a limitation of the study in the revised manuscript (page 15).

To partially address this limitation, we have added post-mortem spinal cord tissue for a subset of 6 ALS cases and 4 disease controls in the revised manuscript and increasing the total number of tissue samples analysed. Taken together, our tissue data provide preliminary biological support for the tissue-level mechanisms underlying the serum and CSF biomarker findings and lay the groundwork for future larger-scale post-mortem studies in this area.

Furthermore, while BD-tau IP in serum seems to indicate the source of elevated p-tau 217 and BD-tau in AD, however the authors have not performed the experiments to show that big tau and phosphorylated big tau could be the potential source in ALS serum. The plasma BD-tau and its consequent increase in prodromal AD and clinical AD compared to controls without amyloid positivity has been well established (Gonzalez-ortiz, F. et al Brain 2022; Gonzalez-ortiz, F. et al Nature Comm 2024, Montoliu-Gaya, Laia et al Nature Aging 2025), however the source of p-tau 217 elevation using commercial immunoassays in ALS need explanation – is it derived from small tau within the CNS or is it derived from the periphery (muscles) that are primarily made up of big tau? This has to be addressed by the authors in this manuscript before this can be considered for publication in Nature Communication.

We thank the reviewer for this important question.

Regarding the central origin of BD-tau and BD-p-tau217 in AD, we refer to the well-established literature cited by the reviewer, with which our BD-Tau-IP serum data are fully consistent. We added the suggested references to our paper.

Here, we would like to the clarify the source of blood p-tau 217 elevation in ALS by combining several lines of experimental evidence from the present study, our previous work (Abu-Rumeileh, Nature Communications 2025) and other studies.

First, Abu Rumeileh et al. (Nature Communications 2025), Cousins et al. (Annals of Neurology 2022), Vacchiano et al. (JNNP 2023), Janelidze et al. (Alzheimer's & Dementia 2025) showed that blood p-tau217 and p-tau181 were elevated in ALS but none of the CSF p-tau biomarkers were increased in ALS compared with controls. This blood-CSF dissociation strongly suggests that the source of elevated blood p-tau in ALS is peripheral rather than central, since a genuinely CNS-derived tau elevation would be expected to be reflected in CSF as well.
These previous observations are fully consistent with our hypothesis that peripheral tissues, rather than the brain, are the primary sources of elevated blood p-tau in ALS. Moreover, our previous phosphoproteomic mass spectrometric analysis of muscle tissue from ALS patients demonstrated the presence of p-tau217 in muscle, confirming that peripheral muscle is a potential source of elevated p-tau217 and p-tau 181 in ALS serum (Abu-Rumeileh, Nature Communications 2025). These aspects have been already discussed in the introduction of the present paper (pages 3-4) as well as in Abu-Rumeileh et al. 2025.

In the initial version of the present manuscript, targeted mass spectrometry analysis of muscle tissue from ALS patients and disease controls without prior tau enrichment detected only big tau isoforms and not BD-tau, suggesting that muscle exclusively expresses big tau. However, as correctly pointed out by reviewers 3 and 4, the low abundance of tau in peripheral tissues compared to brain may have hindered sufficient signal-to-noise for BD-tau peptide detection in direct digest approaches, which is now confirmed by the detection of only minimal BD-tau signal even after IP enrichment, consistent with the very low abundance of this isoform in muscle tissue.

Indeed, we performed tau-specific enrichment prior to MS analysis as suggested and previously mentioned. In detail, we performed a pan-tau IP on brain, spinal cord and muscle samples, followed by targeted mass spectrometry analysis of the IP eluates, to distinguish BD-tau from big tau isoforms. For the detailed results of pan-tau IP followed by MS please refer to answer 4, Point D of reviewer 3 and to the manuscript. Relevant for the present topic was the finding that big tau was the dominant isoform in muscle samples of both ALS patients and controls, with only a minimal BD-tau signal detectable. Notably, this pattern was consistent across ALS patients and controls, indicating that big tau predominance in muscle reflects a biological property of the tissue rather than a disease-specific phenomenon.

To better address this issue, we also performed a pre-analytical immunoprecipitation with a p-tau217-specific antibody on total protein from muscle protein extracts (ALS n=4, controls n=4), followed by targeted mass spectrometry to detect big tau and BD-tau.
Due to limited brain and spinal cord sample availability, we were able to perform only the pan-tau IP in these samples but not also the p-tau217-IP.
For the p-tau217-IP we used functionalized magnetic beads with a p-tau217 polyclonal antibody (#44-744, Thermo Fisher Scientific, Invitrogen, USA). The specificity of this antibody has been previously confirmed and its successful application in post-mortem AD brain tissue has been demonstrated (Ellis 2024, Kavanagh 2025). Further details concerning pan-tau IP and p-tau217 IP methods are reported in methods (pages 20-21) and in supplementary information (pages 11-13). IP eluates were then analysed by means of targeted mass spectrometry for the detection of BD-tau and big tau.

P-tau217-IP followed by MS on muscle tissue detected exclusively big tau and not BD-tau, directly demonstrating that quantitatively relevant phosphorylation at T217 in muscle occurs only on big tau and not on BD-tau species.
This finding is consistent with the pan-tau IP results, which show that while a minimal amount of BD-tau is detectable in muscle after enrichment, this minor BD-tau fraction is not phosphorylated at T217, in contrast to the abundant big tau species, which carry T217 phosphorylation.
Whilst the absence of a brain tissue positive control for the p-tau217 IP represents a limitation, the successful detection of big p-tau217 in muscle samples confirms that the IP enrichment performed accurately. Furthermore, the specificity of the antibody (#44-744, Thermo Fisher Scientific) used for p-tau217-IP has been confirmed and independently validated in post-mortem human brain tissue (Ellis et al. 2024, Kavanagh et al. 2025), providing indirect evidence that our IP-MS assay would have captured BD-p-tau217 had it been present in muscle.

All these findings provide a comprehensive explanation for the apparent elevation of both p-tau217 and BD-tau in ALS serum measured by commercial Simoa immunoassays. Since BD-tau is not phosphorylated at T217 in muscle tissue, the p-tau217 signal detected by direct Simoa assays in ALS serum reflects peripheral big p-tau217 released from degenerating muscle fibres. Similarly, the elevation of serum BD-tau detected by the commercial BD-tau Simoa assay in ALS is driven predominantly by peripheral big tau, as this assay does not discriminate between BD-tau and big tau isoforms, and by a minor contribution of non-phosphorylated BD-tau released from muscle tissue.
Our two-step approach, BD-tau IP followed by either Simoa p-tau217 or Simoa BD-tau measurement, directly resolves this confound by selectively removing peripheral big tau and peripheral big p-tau217. Following BD-tau IP, neither BD-tau nor p-tau217 levels were elevated in ALS compared to controls, in contrast to the significant elevations observed with direct assays, confirming the peripheral origin of the signal detected by commercial assays in ALS.
The absence of elevation in ALS compared to controls for both blood markers contrasts with the robust biomarker elevations observed in AD after BD-Tau-IP, where AD pathology drives significantly increased release of cerebral BD-p-

tau217 and BD-tau. Taken together, these findings validate our BD-tau IP + Simoa strategy as a specific tool for isolating and quantifying specifically BD-tau species in blood.

One important question that warrants explicit discussion is whether the elevated BD-tau detected by commercial Simoa assays in ALS serum could reflect, at least in part, degeneration of spinal cord motor neurons rather than peripheral muscle origin. This is a legitimate concern, given that our pan-tau-IP followed by MS demonstrates that BD-tau is indeed present in spinal cord tissue of both ALS patients and controls. However, several lines of evidence argue against spinal cord motor neuron degeneration as the primary driver of serum BD-tau elevation in ALS with the Simoa assay. Indeed, following BD-tau-IP the serum BD-tau and p-tau217 levels were not elevated in ALS patients compared to controls across the entire cohort. If motor neuron degeneration in the spinal cord was a significant source of BD-tau release into the bloodstream, one would expect at least a significant elevation of these markers after BD-Tau-IP in ALS, which is not observed. Of note, our MS data show a trend toward higher tau levels in the spinal cord of ALS patients at the tissue level, which however does not translate into detectable BD-tau elevation in blood after BD-Tau-IP. This strongly suggests that the BD-tau elevation measured by commercial assays in ALS serum does not reflect genuine CNS-derived BD-tau release from degenerating motor neurons, but rather peripheral big tau being captured non-specifically by the commercial assay.

Notably, low concentrations of both BD-tau and p-tau217 after the two-step approach were still detectable in serum of ALS patients and controls alike. We interpret these residual concentrations as reflecting the physiological baseline release of BD-tau and BD-p-tau217 into the bloodstream, present in all individuals regardless of diagnosis. Specifically, the residual BD-tau concentrations in serum likely reflect a combined contribution from the CNS as the primary source and from muscle tissue as a minor source, consistent with the detection of small amount of non-phosphorylated BD-tau in muscle after pan-tau IP and MS analysis. On the other hand, the residual BD-p-tau217 concentrations in serum of ALS cases most likely originate exclusively from the CNS, given that BD-tau is not phosphorylated at T217 in muscle tissue, as demonstrated by our experiments.

Future studies directly quantifying big p-tau217 and BD-p-tau217 in brain and spinal cord tissue will be important to further characterize the regional distribution of phosphorylated tau isoforms and their respective contributions to blood signals in ALS and AD.

Moreover, although the convergent tissue-specific mass spectrometry and biomarker findings strongly support a peripheral origin of the serum p-tau217 signal in ALS, direct proteomic confirmation of circulating BD-p-tau217 and big p-tau217 species in serum remains an important objective for future studies. Such analyses remain technically challenging due to the very low abundance of tau peptides in blood and the complexity and dynamic range of the blood proteome. Future developments in targeted proteomics workflows, including improved immunoenrichment strategies in combination with high-sensitivity mass spectrometric detection methods, may enable direct validation of BD-tau and big tau species in biofluids.

These new methods (pages 19-20), results (pages 9-10) and the respective discussion (pages 13-14) have been added to the manuscript. We modified also Figure 6 (previously Figure 5).

Reviewer #4 (Remarks to the Author):

Review Comments: Abu-Rumeileh et al., "Selective detection of tau isoforms across brain, muscle and blood in Alzheimer´s disease and amyotrophic lateral sclerosis"

In this study "Selective detection of tau isoforms across brain, muscle and blood in Alzheimer´s disease and amyotrophic lateral sclerosis", Abu-Rumeileh et al. examined the diagnostic utility of BD-tau specific immunoassays for improving specificity of tau-based biomarkers for detection of central (i.e AD) vs peripheral neurodegenerative processes (i.e. ALS). First, they measured serum and CSF brain-derived tau (BD-tau) in ALS (n=139), AD (n=101), and disease controls(n=75) using commercial Simoa immunoassay and found that serum BD-tau was increased in AD> ALS> disease controls whereas CSF BD-tau was increased in AD compared to ALS and disease controls with no difference between the latter two groups. Contrarily, CSF total tau was increased in AD and ALS compared to disease controls. Second, using a "two-step" approach involving an initial BD-tau IP to enrich for brain-derived species followed by immunoassay to serum BD-tau and ptau-217. Whereas pre-IP, serum BD-tau and ptau-217 were increased in AD and ALS compared to disease controls, post-IP, both BD-tau and ptau-217 were only increased in AD suggesting that enrichment of BD-tau increases sensitivity for AD. Finally, the authors examine peptides representative of BD-tau and peripheral tau (big tau) using mass spec in frontal cortex

and muscle tissue from AD, ALS, and controls. In brain, relative levels of BD- and big tau were elevated in AD and a subset of ALS while in muscle, mild increases in big tau were detected in ALS and a disease control.

This study addresses an important question related to how central and peripheral sources of tau contribute to biofluid measures and begins to determine how analytic strategies can be optimized to improve specificity of tau measures for AD.

The strength of the study includes 1) the application of an established BD-tau assay towards a large cohort of ALS (n= 139) and AD (n= 101), 2) stratification of ALS cases based on AD co-pathology, and 3) the use of orthogonal approaches for detecting BD-tau (i.e immunoassay, BD-tau IP enrichment, mass spec). However, the cohort characterization could be improved to provide more clinical insight into disease controls and the authors don't provide concrete evidence that the source of elevated p-tau in the serum of ALS is derived from peripheral tau. Some of the mass spectrometric experiments to establish the peripheral tau in muscles and brain tissue also need further clarification as detailed below.

We are very grateful to the reviewer for the positive comments.

Major points

1. The authors find that serum BD-tau is increased in ALS compared to disease controls after accounting for AD co-pathology. As tau may derive from brain vs peripheral sources and ALS is a disease defined by both upper and lower motor neuron pathology, this work could be strengthened by providing more phenotypic data related to ALS cases, particularly cognitive involvement and UMN vs LMN burden to determine whether BD-tau in ALS is associated with cognitive/ UMN involvement.

We thank the reviewer for this important suggestion. Detailed phenotypic data of the ALS cohort from Halle and Milan have been previously published in Abu-Rumeileh et al. (Nature Communications 2025), to which we refer for a comprehensive clinical characterization. The present study included a subset of the original cohort (123 of 130 patients) based on the availability of biosamples for the new biomarker analyses.
To address the reviewer's concern, we have added, a new supplementary table (Supplementary Table 11) with the phenotypic data of this subgroup, including newly acquired data on cognitive involvement assessed by the Edinburgh Cognitive and Behavioural ALS Screen (ECAS) and the Montreal Cognitive Assessment (MoCA), available for the Milan ALS subgroup (ECAS: n=58; MoCA: n=39), as well as data on upper and lower motor neuron (UMN/LMN) burden, which were available for the entire Halle and Milan cohorts (n=123). UMN and LMN involvement was obtained from medical records and was assessed by examining for the presence or absence of signs at each spinal level (bulbar, cervical, thoracic, and lumbosacral regions; UMN thoracic was not assessed), following the methodology described by Cousins et al. (Ann Neurol 2022).
Regarding cognitive involvement, we found no significant associations between ECAS or MoCA scores and serum BD-tau measured by direct Simoa assay and after BD-Tau-IP. Regarding motor neuron involvement, we found no significant associations between serum BD-tau measured by direct Simoa assay and after BD-Tau-IP and UMN or LMN burden at any spinal level after correction for multiple comparisons. These findings are consistent with our central hypothesis that elevated serum BD-tau measured with direct assay reflects a peripheral muscular source rather than central motor neuron degeneration in ALS.
We also added these issues in the methods (pages 16-17) and results (page 9) in the manuscript.

The disease control cohort should also be better characterized with regard to underlying diagnoses, particularly neurological diagnoses. It is indicated that disease controls were screened clinically, neuroradiologically and by CSF analyses but these criteria should be more clearly defined. For instance, non-neurodegenerative conditions such as multiple sclerosis, traumatic brain injury can predispose to neuronal injury and tau leak.

We thank the reviewer for this important comment. The control group included in the present study is the same cohort previously described and published in Abu-Rumeileh et al. (Nature Communications 2025), to which we refer for a detailed characterization. As reported in that study, non-neurodegenerative disease controls were defined in the first version of the present manuscript as individuals lacking any clinical, neuroradiological, or CSF evidence of neurodegenerative disease.
To address the reviewer's concern more specifically, we have added a more detailed characterization of the control group in the revised manuscript (see supplemementary information page 7). Inclusion criteria for disease controls were: (1) individuals lacking any clinical evidence of neurodegenerative disease; (2) absence of structural brain or spinal cord disease on MRI; (3) absence of CSF evidence of CNS inflammatory disease, including normal white blood cell count and absence of oligoclonal bands; (4) normal Alzheimer's disease CSF biomarkers according to in-house reference values;

and (5) normal serum NfL levels according to in-house reference values (as a sensitive and disease-agnostic filter against subclinical neuronal injury, regardless of the underlying diagnosis).
The disease control group (n=75) comprised individuals with: primary psychiatric disorders, including a subset with mild cognitive symptoms (n=29); subjective cognitive impairment with normal cognitive testing (n=8); first unprovoked seizure or epilepsy without structural brain disease (n=5); primary headache (n=4); idiopathic non-inflammatory polyneuropathy (n=6); non-neurodegenerative movement disorders (e.g. functional movement disorder, facial hemispasm, focal dystonia) (n=8); Horner syndrome (n=2); cranial or peripheral nerve palsies (n=3); and other minor neurological diagnoses (n=10) not associated with neuronal injury.

Importantly, no cases of multiple sclerosis or traumatic brain injury were included in the control cohort, directly addressing the reviewer's specific concerns regarding conditions that may predispose to neuronal injury and tau release.

In addition, it would be helpful to understand whether this group represents subsets that may serve as AD- or ALS mimic conditions.

We thank the reviewer for this important point. We acknowledge that some diagnoses within the disease control group could theoretically represent conditions that partially overlap with AD or ALS clinically. On one side, non-inflammatory polyneuropathy (n=6) and cranial or peripheral nerve palsies (n=3) may occasionally be considered an ALS mimic in the differential diagnosis. On the other side, the subset of subjects with primary psychiatric disorders and mild cognitive symptoms (n=15) and those with subjective cognitive impairment (n=8), could in principle represent a differential diagnosis for AD.
However, as detailed above, all disease control subjects were systematically screened using a multimodal approach including normal MRI, normal CSF inflammatory parameters, normal AD biomarker profile, and normal serum NfL levels, which effectively excludes both preclinical AD and early ALS as well as other neurodegenerative diseases in these individuals.
Furthermore, we would like to point out that the primary purpose of the disease control group in this study was to provide a non-neurodegenerative comparator for the assessment of serum BD-tau and p-tau217 levels in ALS and AD, rather than to investigate differences between specific control diagnoses. Given the small number of subjects in individual diagnostic subgroups that could theoretically act as AD or ALS mimics, any subgroup-specific comparison would be statistically underpowered and would not provide meaningful additional insight into the main findings of the paper. We have further clarified the composition of the disease control group in the revised supplementary methods (page 7).

2. The study utilizes postmortem frontal cortex tissue from AD (n=4), ALS (n=7, 3 C9, 4 sALS) and disease control (n= 5) for mass spec of small tau and big tau species. However, the postmortem intervals for a number of the tissues are quite prolonged > 48 hours which applies to 2 AD, 3 ALS, 3 disease controls. Since cytoskeletal proteins (i.e. tubulin, neurofilament) are known to have reduced staining intensity by 48 hr and lose signal by immunoblot even earlier < 24hr, this caveat should be highlighted in the data. Although sample size is too low to stratify based on PMI, the authors could consider designating samples with long PMI >48hr by asterisk or other indicator.

We thank the reviewer for raising this important methodological point. We fully agree that prolonged post-mortem intervals (PMI) may affect the integrity of cytoskeletal proteins, including tau isoforms, and that this represents a relevant caveat for the interpretation of our mass spectrometry data.
Following the reviewer's suggestion, we have designated samples with a PMI >48 hours with an ¥ in the supplementary table 13, allowing the reader to visually identify these cases and interpret the data accordingly.
Furthermore, we have explicitly acknowledged the potential impact of prolonged PMI on tau protein integrity as a limitation of the study in the revised Discussion section (page 15), alongside the limited sample size of neuropathological samples, which precluded formal statistical stratification by PMI.

3. Given the differences in age across cohorts, the BD-tau data was adjusted for age but it is unclear why sex adjustment was not also made. The authors should show data suggesting no effect of sex on biomarker levels though their prior study showed sex affected ptau-181 and ptau-217 measures.
We thank the reviewer for this important observation and acknowledge the lack of clarity in the original manuscript. We note that this issue was also raised by the handling editor, who requested sex-disaggregated reporting of biomarker data, as detailed in our response to the handling editor.
To clarify: in the present cohort, serum and CSF BD-tau, CSF t-tau, BD-tau after BD-Tau-IP, and p-tau217 after BD-Tau-IP were not significantly influenced by sex, as shown in the supplementary table 6, and adjustment for sex was therefore not required for analyses concerning these biomarkers. However, serum p-tau217 measured by direct Simoa assay was significantly higher in males than in females (supplementary table 6), consistent with our previous findings reported in Abu-Rumeileh et al. (Nature Communications 2025). We acknowledge that this was not sufficiently specified in the

original manuscript and have clarified this point in the revised version of the results (page 5). Sex-disaggregated biomarker levels and the results of statistical comparisons between males and females are provided in the supplementary table 6.
We added the analyses of serum p-tau217 by direct Simoa assay, which were performed after adjustment for both age and sex as reported in Abu-Rumeileh et al. (Nature Communications 2025), confirming the same diagnostic and group-level findings of the previous paper (page 6).
Of note, BD-p-tau217 measured after BD-Tau-IP was not influenced by sex (page 7 of the result section, page 3 of supplementary information), which further supports the notion that the BD-Tau-IP step removes peripheral big-p-tau217, which may contribute to the sex effect observed in direct serum p-tau217 measurements.

4. The serum BD-tau measures were performed on a larger cohort of ALS (n= 139), AD (n= 101), and disease controls (n=75). However, CSF analysis was only performed on a subset of samples available. The authors should comment on how the subset was chosen as well as rationale for using smaller cohort.

We thank the reviewer for this comment. CSF BD-tau measurements were performed in a subset of 143 individuals from the Halle and Milan cohorts (61 ALS, 51 A and 31 disease controls), representing those participants for whom sufficient residual CSF volume was available after prior use in previous studies, including Abu-Rumeileh et al. (Nature Communications 2025). This subset was selected solely on the basis of sample availability, without any clinical or biological selection criteria, and is therefore not expected to introduce systematic bias in the CSF analyses. We acknowledge the smaller CSF cohort size as a limitation in the revised manuscript (page 15).

5. While the BD-tau assay on the commercial platform captures the brain derived small tau isoforms, the authors need to define more clearly the purpose of the pre-analytical BD-tau IP. Are they enriching for BD-tau using the antibody and then performing Simoa or are they preclearing the BD-tau? The process needs clarification as readers who are not familiar with immunoprecipitation process would think this preanalytical process is meant to remove the BD-tau from the serum.

We thank the reviewer for this important point of clarification. This question overlaps substantially with question 1 of Reviewer 3. For completeness, we provide a brief summary here: the BD-Tau-IP is a selective enrichment procedure, not a preclearing step. The anti-BD-tau antibody mAb 2B8, coupled to magnetic beads, selectively captures BD-tau isoforms from serum via their exon 4-5 junction epitope, while peripheral big tau, which lacks this epitope, remains in the supernatant and is discarded. The BD-tau-enriched eluate is then used for downstream Simoa quantification of BD-tau and/or p-tau217. In other words, the procedure concentrates BD-tau while eliminating interfering peripheral tau species, thereby improving specificity of the downstream measurement. Notably, our results indicate that the commercial BD-tau Simoa assay alone does not fully discriminate BD-tau from peripheral big tau, underscoring why this enrichment step is necessary. For the full methodological description please refer to the detailed response to question 1 of Reviewer 3.

On pages 7-8, the authors performed p-tau 217 analysis using Simoa only and BD-tau IP methods but the description of the data is not clearly written. The difference between the semi-automated IP format and manual BD-tau IP should also be better defined in the methods section.
We thank the reviewer for this comment. This question overlaps substantially with question 2 of Reviewer 3. In brief, we have made the following substantive changes to address this:
In the Results section, we reorganised the description of serum BD-tau and p-tau217 findings into four clearly labelled subsections: (1) direct one-step approach (Simoa measurements, including p-tau217) (2) two-step approach with semi-automated BD-Tau-IP followed by BD-tau and p-tau 217 quantification, (3) comparative diagnostic accuracy (one-step vs. two-step approach), and (4) two-step approach with manual BD-Tau-IP.
In the Methods section and supplementary methods, we added dedicated paragraphs clearly defining the key differences between the manual and semi-automated protocols, including starting serum volume, elution volume, dilution factors, and degree of automation. The rationale for presenting the semi-automated protocol results in the main text, being the largest and most representative cohort, is now explicitly stated.
For the full detailed response please refer to answer to question 2 of Reviewer 3.

In addition, the preanalytical process was done in a smaller cohort subset compared to direct Simoa measurements. Any particular reason why? Was sample availability the reason for the choice?
This question overlaps to the comment 3 raised by Reviewer 3. Sample availability was the primary reason underlying the smaller cohort size for the two-step approach analyses compared to direct Simoa measurements. Indeed, the two-step approach with BD-Tau-IP requires a relatively large starting serum volume per patient (up to 1,000 µL for the first manual IP and 800 µL for the semi-automated IP), which substantially limits the number of samples that can be processed. Moreover, in the same cohort, serum samples had already been used for direct Simoa measurements of multiple

biomarkers (BD-tau, p-tau 181, p-tau 271, tau, NfL) for this study and our previous one (Abu-Rumeileh et al. Nature Communications 2025), which progressively reduced the available volume for subsequent BD-Tau-IP experiments. As a result, BD-Tau-IP procedures were performed in subsets of patients for whom sufficient residual serum volume was available. We acknowledge this as a limitation of the current study and have added a statement in the discussion of the revised manuscript. For further details, please refer to comment 3 by Reviewer 3.

6. Page 9: Mass spec analysis of BD-tau and big tau in brain and muscle samples are not properly defined and multiple aspects of the data has to be explained as described below.

a. While serum samples were tested with BD-tau IP, searching through the supplementary methods shows no enrichment strategies were employed for these tissue samples. 10 µg of total protein from brain or muscle homogenates were digested with enzymes and specific tau peptides for small tau and big tau were targeted on the mass spec. The authors claim they found no BD-tau peptide in the muscle biopsies from ALS (n=5) and disease controls (n=5), only observing one big tau peptide form exon 4a in these muscle tissues. The authors also found both big tau and BD-tau specific peptide in the brains. Without an enrichment step, it would be difficult to conclude absence of BD-tau in muscle.
The concentration of tau protein in the periphery is 1000-fold lower compared to cortical brain tissues (PMID: 26890756; PMID: 37330193, PMID: 37122097). The claim that BD-tau specific peptide is absent in muscle compared to big tau (exon 4a) peptide should be validated using tau specific enrichment. Using any tau specific antibody (HT7 that binds to mid-domain of tau isoforms) should be able to enrich tau in these samples and ratio of BD-tau/big tau signals would be more confident unlike what is currently being presented in Figure 4.

We thank the reviewer for this important methodological point, which is closely related to question 4, point D, raised by Reviewer 3. In summary, we fully agree that pre-analytical tau enrichment was necessary to confidently detect and quantify BD-tau in peripheral tissues. We have therefore performed immunoprecipitation-based tau enrichment using a pan-tau antibody prior to targeted MS analysis on brain, spinal cord, and muscle samples. Following enrichment, both BD-tau and big tau peptides were detectable across all tissue compartments. Importantly, in muscle, big tau was the dominant species in both ALS patients and controls, with only minimal BD-tau signal detectable after enrichment, confirming that the original failure to detect BD-tau in muscle reflected insufficient signal-to-noise rather than true absence. The BD-tau/big tau ratio demonstrated a clear central-to-peripheral gradient, with muscle consistently showing the lowest ratio and brain and spinal cord showing higher but overlapping values. Figure 4 has been updated accordingly. For full methodological details and results please refer to answer to question 4, point D of Reviewer 3.

b. The authors do not provide evidence of other tau peptides in their measurements (N-terminus or proline rich region of tau) to help assess proportion of small tau vs big tau in these tissues. Does the muscle tau contain 3R and 4R (microtubule binding region) repeats?

We thank the reviewer for this comment. Please refer to the full answer to question 4a of reviewer 3, which overlaps with this question. In summary, we acknowledge that our mass spectrometry analysis was intentionally focused on two specific tau species, BD-tau (small tau, with exon 4/5 junction peptide) and big tau (with exon 4a peptide), without profiling the full tau peptide spectrum including N-terminal, mid-domain, and microtubule binding region (MTBR) peptides. This was a deliberate choice reflecting the primary aim of our study, which was to develop and validate BD-tau and BD-p-tau217 as new biomarkers in AD in the comparison to ALS and especially to better understand our findings on serum using BD-Tau-IP, by characterizing the distribution of BD-tau and big tau isoforms in clinically relevant biofluids and tissues.

A comprehensive atlas of tau isoform distribution across tissues was beyond the scope of the present work. For this issue, we refer the reviewer to the study by Koppisetti et al. (Annals of Neurology 2026, in press), which performed an exhaustive IP-MS characterization of tau peptides, including N-terminal, mid-domain, MTBR, and exon 4a-L specific peptides, across multiple CNS and PNS tissues in ALS and control subjects and across brain tissue in AD, ALS and controls subjects (Table 2). The accepted version of this paper is currently available at https://www.biorxiv.org/content/10.1101/2025.09.15.676127v2.

Regarding the specific domains raised by the reviewer, we note that among the peptides monitored in our updated assay, IATPRGAAPPGQK covers the proline-rich region (mid-domain) and serves as a normalization reference, partially addressing the concern regarding mid-domain coverage. However, true N-terminal and MTBR peptides including 3R/4R-specific sequences were not targeted in our assay, as profiling these domains was beyond the scope of the present study.

Regarding the specific question on 3R and 4R isoforms in peripheral tissues, we refer the reviewer to Koppisetti et al. (Annals Neurol 2026, in press), who detected both 3R and 4R specific peptides in high molecular weight bands from PNS

samples including sciatic nerve and brachial plexus, demonstrating that big tau in peripheral nervous tissues contains microtubule binding repeats. Notably, Koppisetti et al. also quantified 3R and 4R isoform expression across the nervous system, observing a significant decrease in 4R isoform levels in spinal cord and sciatic nerve compared to brain, while 3R isoform levels remained unaltered across these tissues. Whether the same 3R/4R distribution applies to skeletal muscle big tau was not directly addressed by Koppisetti et al. and remains to be investigated.

c. The choice of peptides for the mass spectrometry analysis seems limited. Using Asp-N and Lys-C they derive the DEAAGHVTQARMVSK that is exon 4- exon 5 bridging peptide and is specific for small tau. However, they only monitor and target single peptide from the exon 4a of big tau. The exon 4a is quite large and the authors should monitor more than one peptide to ensure they have covered the big tau sequence.
This question overlaps with question 4b of reviewer 3. We agree that data for the detection of big tau benefit from monitoring more than one peptide. We have improved the targeted MS method based on this comment to include a second peptide from exon 4a (DTEGGRHAPELLK), in addition to the previously monitored exon 4a peptide (VSTEIPASEPDGPSVGRAK), and two peptides common to all tau isoforms (LTFRENAK and IATPRGAAPPGQK) for normalization purposes. The location of all monitored peptides within the BD-tau and big tau sequences is illustrated in the updated Figure 3A, which shows the exon 4a-specific peptides for big tau (yellow), the BD-tau-specific bridging peptide spanning the exon 4–5 junction (red), and the common tau peptides (grey).

In the discussion the authors cite Koppisetti, R. et. al biorxiv (2025) that mentions another big tau isoform that they call exon 4a-long and distinct from exon 4a. Do the authors find any peptides from exon 4a-long in any of their tissue samples?

We thank the reviewer for this important question arising from the most recently published results. The design of the targeted mass spectrometry approach utilized in this study was independent from the results presented by Koppisetti et al. and did not specifically target peptides for exon 4a-long (4a-L) or for exon 4a-short (4a-S) as these authors did. However, to directly address the question of the reviewer, we additionally analysed two muscle samples after pan-tau-IP using an untargeted, data-dependent mass spectrometry approach, incorporating the newly described exon 4a-L sequence into the protein database search space.
Using this approach, we identified two peptides specific to exon 4a-L, spanning the junction from exon 4 to the exon 4a-L and spanning from the additional amino acids in exon 4a-long to exon4a. Notably we also identified the peptide specific for exon-4a-S spanning the direct exon 4 to exon 4a junction without the additional amino acids from exon 4a-long. These findings indicate that a mixture of both exon 4a-L and exon 4a-S is expressed in muscle.
Importantly, this result is fully consistent with Koppisetti et al., who demonstrated that exon 4a-L is the predominant form of big tau (>95%) across human nervous tissues, further validating that our targeted assay captures the biologically dominant big tau isoform.
The measurement methods have been added to the methods (pages 20-21) and supplementary methods (page 13-14). The results have been added to the manuscript (page 10), supplementary results (page 6) and an additional supplementary figure have been included (Supplementary Figure 4).

Furthermore, we would like to highlight that the two big tau peptides monitored in our targeted PRM assay (VSTEIPASEPDGPSVGRAK and DTEGGRHAPELLK) are located within the exon 4a sequence shared between both 4a-L and 4a-S isoforms, and are therefore present in both big tau variants described by Koppisetti et al. as can be inferred from the sequence alignment shown in Figure 1C of Koppisetti et al. 2026. We added this information in the methods (page 20).

d. ALS impacts upper and lower motor neurons. Is it possible for the authors to test spinal cords and nerves to demonstrate the changes they observe in BD-tau and big tau are also present in these ALS affected tissues. Although muscle may be a source for big tau in the periphery, nerve should also be explored.
Please refer to the full answer to question 4E of Reviewer 3, which addresses the same topic in detail. In summary, we have included spinal cord samples from ALS and disease controls in our updated analysis, directly addressing lower motor neuron pathology.
Regarding the brain samples, we would like to clarify that all ALS brain samples included in our study were derived from the frontal cortex, specifically including the primary motor cortex, addressing upper motor neuron pathology.
Together, these two tissue compartments provide a comprehensive assessment of BD-tau and big tau distribution across both UMN and LMN populations affected in ALS. Regarding peripheral nerve tissue, we acknowledge this as a limitation, nerve samples were unfortunately not available in our cohort. However, we refer the reviewer to Koppisetti et al. (Annals Neurol 2026, in press), who characterized BD-tau and big tau distribution in sciatic nerve, brachial plexus and dorsal root ganglion from ALS cases and controls, demonstrating that peripheral nerve tissues are enriched in big tau compared to CNS tissues, consistent with our findings in skeletal muscle. We added this information in the discussion.

e. The authors need to use common tau peptides to normalize their target mass spec peptide intensities that will strengthen some of their claims that indicate BD-tau expression is increased in these ALS patients.
Please refer to question 4F of reviewer 3. In short, we included common tau peptides in an updated PRM method for the additionally conducted experiments. Results are presented as BD-tau/t-tau, big tau/t-tau and BD-tau/big tau-ratios. The raw PRM intensities are now presented in Supplementary Figure 3.

7. The authors in the discussion claim this paper clarifies the distinct biological origins of BD-tau and big tau across different tissues and disease states. However, the sample numbers are too low per group to make any conclusions.
Please refer to answer to question 5 of reviewer 3. 5.
We acknowledge that the number of post-mortem tissue samples analysed is limited, as is inherent to studies using human post-mortem brain tissue, where availability is constrained by ethical, logistical, and neuropathological quality requirements. Nevertheless, our sample size is consistent with prior MS-based studies investigating biomarker distribution in human post-mortem CNS and peripheral tissues (Oeckl, et al., 2020; Koppisetti et al., 2026). However, we fully acknowledge the reviewer's concern. Our tissue experiments were designed as a mechanistic validation study, primarily aimed at providing direct biological evidence for the tissue-level origin of the biomarker alterations observed in serum and CSF.
We recognize that the limited sample size precludes definitive conclusions regarding tissue-specific tau isoform distribution, and we have explicitly acknowledged this as a limitation of the study in the revised manuscript.
To partially address this limitation, we have added post-mortem spinal cord tissue for a subset of 6 ALS cases and 4 disease controls in the revised manuscript and increasing the total number of tissue samples analysed. Taken together, our tissue data provide preliminary biological support for the tissue-level mechanisms underlying the serum and CSF biomarker findings and lay the groundwork for future larger-scale post-mortem studies in this area.

Furthermore, BD-tau IP in serum seems to indicate the source of elevated p-tau 217 and BD-tau in AD is centrally derived. The plasma BD-tau increase in late stages of AD compared to controls has been well established (Gonzalez-ortiz, F. et al Brain 2022; Montoliu-Gaya, Laia et al Nature Aging 2025). However, the source of p-tau 217 elevation using commercial immunoassays in ALS need explanation – is it derived from small tau from brain or other nerves within the CNS or is it derived from the periphery (muscles) that are primarily made up of big tau (PNS tau)? This has to be addressed by the authors with experimental evidence to clarify the source of big tau and phosphorylated big tau in ALS serum by the authors in this manuscript.

We thank the reviewer for this important comment. Regarding the central origin of BD-tau and BD-p-tau217 in AD, we refer to the well-established literature cited by the reviewer, with which our serum data with the two-step approach are fully consistent.
Regarding the source of elevated serum p-tau217 and BD-tau in ALS, we provide here a brief summary of the experimental evidence, with full details in the response to question 5 of Reviewer 3, which largely overlaps with this comment.
Several converging lines of evidence support a peripheral muscular origin rather than a CNS origin of blood p-tau217 elevation in ALS. First, blood p-tau217 and p-tau181 are elevated in ALS while CSF p-tau biomarkers are not, suggesting a peripheral rather than central source.
Second, pan-tau-IP followed by MS on muscle tissue demonstrated that big tau is the dominant tau species in muscle, with only minimal BD-tau detectable. Third, and most critically, p-tau217-IP followed by MS on muscle detected only big p-tau217 and no BD-p-tau217, directly demonstrating that quantitatively relevant T217 phosphorylation in muscle occurs only on big tau isoforms. Finally, following BD-Tau-IP, neither serum BD-tau nor p-tau217 were elevated in ALS compared to controls, confirming that the elevations observed with direct commercial assays reflect peripheral big tau cross-reactivity rather than genuine CNS-derived BD-tau release.
However, while the tissue-specific mass spectrometry data together with the biomarker findings strongly suggest that the elevated serum p-tau217 signal observed in ALS originates predominantly from peripheral sources, direct proteomic characterization of circulating BD-p-tau217 and big p-tau217 species in serum will be required to definitively confirm this hypothesis.
Please refer to answer to question 5 of Reviewer 3 for the full experimental details and discussion.

RESPONSE TO REVIEWERS' COMMENTS

Reviewer #1

The authors adequately addressed my comments.

We sincerely thank the reviewer for the careful evaluation of our manuscript and for the positive assessment of our revised version.

Reviewer #2

I co-reviewed this manuscript with one of the reviewers who provided the listed reports. This is part of the Nature Communications initiative to facilitate training in peer review and to provide appropriate recognition for Early Career Researchers who co-review manuscripts.

We thank the reviewer for the careful evaluation of our work and for the time devoted to reviewing our manuscript.

Reviewer #3

Review Comments: Abu-Rumeileh et al., "Tau isoforms mapping across tissues and biofluids reveals a signature distinguishing Alzheimer´s disease from amyotrophic lateral sclerosis"

The authors of the study have now thoroughly addressed most of the points raised by the reviewer and have provided details regarding their approach to measure BD-tau and BD-p-tau217 in the tissues investigated. Especially the mass spectrometry results are very convincing, and details provided for all the workflows will be very useful for future validations and reproducibility. The authors clearly demonstrate that in AD brain derived tau and the corresponding brain derived p-tau217 is highly specific for changes associated with AD. Monitoring BD-p-tau217 not only increases the sensitivity to detect changes due to AD pathology, but is no more influenced by the peripheral tau proteoform contribution in other degenerative diseases such as amyotrophic lateral sclerosis (ALS) that may involve different disease mechanism beyond amyloid-beta driven tau pathology. They also provide evidence for diverse tau isoforms in the periphery (big tau) such as muscle biopsies and spinal cord tissues that are in line with current literature.

This study addresses an important question related to how central and peripheral sources of tau contribute to biofluid measures and begins to determine how analytic strategies can be optimized to improve specificity of tau measures for AD.

We sincerely thank the reviewer for the careful re-evaluation of our manuscript and for the positive comments on our revised work. We greatly appreciate the recognition that the additional mass spectrometry experiments, methodological clarifications, and revised analyses have substantially strengthened the manuscript. We are also grateful for the thoughtful and constructive remaining suggestions, which have helped us to further improve the clarity and presentation of our findings. Below, we address each comment in detail.

The reviewer feels the manuscript could benefit from few things:

1. New BD-tau and BD-p-tau217 assays from NULISA and other vendors are also becoming available. Could they be also useful and have an advantage when it comes to high-throughput for measuring these biomarkers.

We thank the reviewer for this important and timely comment. We agree that emerging NULISA-based assays for BD-tau and BD-pTau217, as well as assays developed on other platforms, may offer important advantages for large-scale biomarker studies, including high analytical sensitivity, low sample-volume requirements, automation, multiplexing, and high-throughput processing.

Published evidence is currently most advanced for NULISA-based BD-p-tau assays. Jiang et al. recently reported that NULISA BD-pTau217 showed slightly higher performance compared with Simoa ALZpath p-tau217 for the classification of amyloid pathology and improved identification of tau positivity among amyloid-positive individuals (Jiang et al., 2026). Similarly, Trelle et al. showed that, within the NULISA CNS panel, BD-p-tau217 had numerically higher accuracy than total p-tau217 for detecting amyloid-PET positivity, although this difference did not reach statistical significance (Trelle et al., 2026).

To our knowledge, no peer-reviewed study has yet directly compared non-phosphorylated NULISA BD-tau with total tau, either in AD or in other neurological conditions. Moreover, published NULISA studies of BD-p-tau species have primarily focused on cohorts characterized according to AD-related amyloid or tau pathology. Neither NULISA BD-tau nor BD-ptau217 has, to our knowledge, been independently evaluated in ALS or other disorders characterized by substantial peripheral tau release. Therefore, their ability to exclude interference from muscle- or other peripherally derived big tau species in these conditions remains to be established.

We consider NULISA and our two-step approach with BD-Tau-IP to be complementary rather than competing strategies. NULISA provides clear advantages in terms of automation, scalability, multiplexing, and sample-volume requirements. In contrast, BD-Tau-IP physically enriches BD-tau before downstream measurement. This enables the enriched fraction to be analysed using different analytical platforms, potentially permits multiple tau analytes to be measured from the same eluate, and allows direct comparison of biomarker measurements before and after selective BD-tau enrichment. Previous work combining pre-analytical Tau-IP with fully automated Lumipulse p-tau181 measurements supports the feasibility of coupling Tau-IP to different downstream immunoassay platforms (Morgado et al., 2025). In the present study, the biological interpretation of our two-step results was further supported by orthogonal tissue mass spectrometry and by direct evaluation in ALS, a disease associated with peripheral big tau and big p-tau217 release. However, BD-Tau-IP currently requires larger sample volumes and additional processing steps, has lower throughput, and introduces further requirements for standardization of immunoprecipitation recovery and elution.

Future head-to-head studies comparing NULISA-based BD-tau assays with BD-Tau-IP across AD, ALS, and other conditions associated with peripheral tau release will be important to define their relative analytical and clinical performance.

We have expanded the Discussion to provide a balanced consideration of these emerging high-throughput approaches and their comparison with our two-step approach (page 13).

2. While the authors do highlight that peripheral tau and big p-tau contribution could be the reason for the serum tau increase in ALS independent of AD-pathology, do they predict ALS patients would benefit from the development of such assay and diagnose pathological associated with lower motor neurons.

We thank the reviewer for this important observation. We agree that distinguishing BD-tau from peripheral tau species may also have relevant implications for patients with ALS. Previous studies have linked elevated blood p-tau181 and/or p-tau217 to LMN-predominant phenotypes and clinical or electrophysiological measures of denervation (Cousins et al., 2022; Vacchiano et al., 2023; Verde et al., 2023; Janelidze et al., 2025; Kawarabayashi et al., 2026). Blood p-tau181 was also associated with blood creatine kinase in Verde et al., whereas both serum p-tau181 and p-tau217 were associated with blood troponin in our previous multicentre study (Abu-Rumeileh et al., 2025). Moreover, increased p-tau181 and p-tau217 immunoreactivity was observed in atrophic muscle fibres (Verde et al., 2023; Abu-Rumeileh et al., 2025).

These converging findings as well as the data of the present study support an association among blood p-tau, LMN-related denervation, and muscle pathology. Accordingly, dedicated assays selectively targeting big tau or big p-tau species could potentially serve as biomarkers of denervation-associated muscle pathology in ALS, with possible applications in phenotypic stratification, non-invasive assessment of denervation burden, longitudinal disease monitoring, and evaluation of treatment response.

We note that big tau assays remain to be developed, as the isoform-specific identification of big tau in the present study was achieved by mass spectrometry in tissue, and the feasibility of selectively quantifying big tau species in blood has yet to be demonstrated.
In addition, circulating peripheral tau should not yet be interpreted as a direct measure of motor neuron loss, as it may originate from both peripheral motor axons and denervated muscle fibres. Thus, its clinical utility in ALS will require longitudinal validation against clinical and electrophysiological measures of denervation in larger studies, as well as evaluation in other neuromuscular disorders.

In addition, BD-tau-selective assays may benefit patients with ALS by reducing interference from peripheral tau species and facilitating the identification of concomitant AD pathology, as illustrated in Figure 6.
We have added these considerations to the Discussion (page 15).

3. The mass spec data is now very convincing and much improved, however the z-scored heatmaps do not do justice to the distribution of tau isoforms across the different tissues. Scatter plots of the ratios of BD-tau and big tau compared to total tau for all the tissues investigated would be better as they provide how these two tau isoforms change from CNS to PNS.

We thank the reviewer for this helpful suggestion and agree that scatter plots provide a more transparent representation of the distribution of tau isoforms across tissues. Accordingly, we have added scatter plots showing the individual BD-tau/t-tau and big tau/t-tau ratios in frontal cortex, spinal cord, and muscle (new Figure 4). These plots now constitute the primary visualization of the mass spectrometry data and more directly demonstrate the progressive shift from BD-tau/t-tau predominance in the CNS to big-tau/t-tau predominance in peripheral tissue.

We have also added supplementary Table 12 and supplementary Figure 4 to report the median and interquartile range of both ratios for each tissue. Given the exploratory nature and limited sample size of the tissue analyses, these data are presented descriptively without formal between-group statistical testing.

We would like to retain the z-scored heatmaps as a main figure (now Figure 5), as they provide information not contained in Figure 4. Panel 5C displays an independent experiment, the p-tau217-immunoprecipitation; Figure 4 is restricted to pan-tau immunoprecipitation. Moreover, panel 5B provides unsupervised hierarchical clustering of all individual samples, showing that tissue of origin, and not diagnosis, drives the structure of the data. We therefore consider the two figures complementary. Should the Reviewer and the Editor prefer, we would of course be happy to move Figure 5 to the Supplementary Material.

P-tau217 IP results are interesting and highlights the peripheral tissues indeed have big tau with minimal contribution from exon4-5 specific tau isoform (small tau/BD-tau).

We thank the reviewer for the positive comment.

4. In light of the pan-tau IP from the muscles tissues and the author noticing exon 4a-L and exon 4a-S tau isoforms, it would be more appropriate for the authors to change the tau schematic to include both the big tau isoforms (exon 4a-L and exon 4a-S) in figure 3.

We thank the reviewer for this helpful suggestion. We agree that the figure should better reflect the untargeted proteomic findings. Accordingly, Figure 3 has been revised to include both the exon 4a-L and exon 4a-S big tau isoforms. The peptide colour coding has also been expanded to distinguish peptides shared by both big tau isoforms from isoform-specific peptides. Representative MS/MS spectra confirming the exon 4a-L- and exon 4a-S-specific peptides are provided in Supplementary Figure 3. The figure legend has been modified accordingly.

The untargeted pan-tau IP proteomics results are interesting as they provide evidence for exon 4a-S isoforms. What is the relative contribution of the exon 4a-L and exon 4a-S in the muscles?

We thank the Reviewer for this comment. Regarding the relative abundance of exon 4a-L and exon 4a-S in muscle, our untargeted data-dependent acquisition (DDA) mass spectrometry analysis was designed to establish the targeted assay and assess the presence of both splice variants, rather than quantify their relative contribution. The number of samples analyzed for this purpose is not sufficient to draw a conclusion on the ratio of exon 4a-L and exon 4a-S.
In addition, peptides specific for exon 4a-L and exon 4a-S differ in ionisation and behaviour in the mass spectrometer, which affects their relative detectability; consequently, spectral counts and precursor intensities cannot be used to calculate molar ratios of the two isoforms in the absence of labelled reference peptides.
Moreover, we note that our targeted PRM assay does not resolve this question either, as it was designed for pan-tau and big tau–defining peptides and does not include proteotypic peptides discriminating the two big tau variants, nor stable isotope-labelled internal standards. Reliable relative quantification would require a dedicated targeted assay built specifically for this purpose, which we regard as a priority for a future study. We have clarified this limitation in the Results (page 9) and in the Discussion (page 14).

Reviewer #4

The authors have substantially addressed all major initial concerns raised in the prior review. In particular, they have clarified the characteristics of their disease control cohort with defined inclusion criteria as well as provided correlations between ALS cognitive and UMN/LMN phenotype to serum BD-tau which support their interpretation of BD-tau. The restructuring of the Methods to distinguish and clarify direct Simoa, semi-automated IP, and manual IP workflows. The mass spec work has also been improved by adding tau enrichment to enable detection of BD-tau for normalization, inclusion of additional big-tau peptides, including a subset of paired spinal cord to analyses, and identified postmortem samples with prolonged PMI to inform interpretation.

We sincerely thank the reviewer for the thoughtful re-evaluation of our manuscript and for the very positive assessment.
