# Supplementary material for: Tau isoform mapping across tissues and biofluids reveals a signature distinguishing Alzheimer’s disease from amyotrophic lateral sclerosis
Source: Nat Commun. 2026 Oct 22;17:10745. doi: 10.1038/s41467-026-78390-3 (PMC13691104; doi:10.1038/s41467-026-78390-3)
Supplement: Supplementary file 3 [file 41467_2026_78390_MOESM3_ESM.pdf]

nature portfolio

Corresponding author(s): Prof. Markus Otto

Last updated by author(s): Aug 24, 2026

Reporting Summary

Nature Portfolio wishes to improve the reproducibility of the work that we publish. This form provides structure for consistency and transparency in reporting. For further information on Nature Portfolio policies, see our Editorial Policies and the Editorial Policy Checklist.

Statistics

For all statistical analyses, confirm that the following items are present in the figure legend, table legend, main text, or Methods section.

n/a	Confirmed	
☐	☒	The exact sample size (n) for each experimental group/condition, given as a discrete number and unit of measurement
☐	☒	A statement on whether measurements were taken from distinct samples or whether the same sample was measured repeatedly
☐	☒	The statistical test(s) used AND whether they are one- or two-sided<br>*Only common tests should be described solely by name; describe more complex techniques in the Methods section.*
☐	☒	A description of all covariates tested
☐	☒	A description of any assumptions or corrections, such as tests of normality and adjustment for multiple comparisons
☐	☒	A full description of the statistical parameters including central tendency (e.g. means) or other basic estimates (e.g. regression coefficient) AND variation (e.g. standard deviation) or associated estimates of uncertainty (e.g. confidence intervals)
☐	☒	For null hypothesis testing, the test statistic (e.g. F, t, r) with confidence intervals, effect sizes, degrees of freedom and P value noted<br>*Give P values as exact values whenever suitable.*
☒	☐	For Bayesian analysis, information on the choice of priors and Markov chain Monte Carlo settings
☒	☐	For hierarchical and complex designs, identification of the appropriate level for tests and full reporting of outcomes
☐	☒	Estimates of effect sizes (e.g. Cohen's d, Pearson's r), indicating how they were calculated

Our web collection on statistics for biologists contains articles on many of the points above.

Software and code

Policy information about availability of computer code

Data collection	We collected data in Excel (Microsoft)
Data analysis	We used IBM SPSS Statistics V.21 (IBM), GraphPad Prism V.7 (GraphPad Software, La Jolla, California, USA), R software V.4.0.2 (R foundation, Vienna, Austria), Skyline v25.1.0.237 (MacCoss Laboratory, University of Washington, Seattle, WA, USA) and Maxquant (Version 2.6.1.0, Max Planck Institute of Biochemistry, Martinsried, Germany)

For manuscripts utilizing custom algorithms or software that are central to the research but not yet described in published literature, software must be made available to editors and reviewers. We strongly encourage code deposition in a community repository (e.g. GitHub). See the Nature Portfolio guidelines for submitting code & software for further information.

Data

Policy information about availability of data

All manuscripts must include a data availability statement. This statement should provide the following information, where applicable:

- Accession codes, unique identifiers, or web links for publicly available datasets
- A description of any restrictions on data availability
- For clinical datasets or third party data, please ensure that the statement adheres to our policy

The biomarker data underlying all figures and tables in this study are provided in the Source Data file. Source data are provided with this paper. Mass spectrometry data generated in this study have been also deposited in the ProteomeXchange Consortium database via the PRIDE partner repository under the identifier PXD079838 (pan-tau-IP-PRM) [http://proteomecentral.proteomexchange.org/cgi/GetDataset?ID=PXD079838], PXD079878 (p-tau217-IP-PRM) [http://

proteomecentral.proteomexchange.org/cgi/GetDataset?ID=PXD079878] and PXD079895 (pan-tau-IP untargeted) [http://proteomecentral.proteomexchange.org/cgi/GetDataset?ID=PXD079895] 51,52. Individual-level clinical data are under restricted access because they contain patient information that cannot be fully anonymised under the EU General Data Protection Regulation and the terms of the ethics approvals. Access can be requested in writing from the corresponding author (markus.otto@uk-halle.de); requests will be reviewed by the investigators and the respective institutions, answered within eight weeks, and granted for non-commercial academic research for ten years following publication.

Research involving human participants, their data, or biological material

Policy information about studies with human participants or human data. See also policy information about sex, gender (identity/presentation), and sexual orientation and race, ethnicity and racism.

Reporting on sex and gender	Sex was not a primary variable in the study design. Original study design was aimed at evaluating the diagnostic accuracy of serum and CSF biomarkers and comparing measurement techniques across diagnostic groups. However, sex was recorded for all participants as part of routine demographic data collection. Sex was obtained from medical records and reflects legally registered sex. Self-reported gender was not assessed. Sex data are reported in table 1. Sex information and consent for reporting and sharing individual-level data have been obtained. We performed a post-hoc examination of sex differences in biomarker levels across the whole cohort (females n=157, males n=180). Serum p-tau217 levels were significantly higher in males than in females (p=0.005), whereas no significant sex-related differences were observed for the other investigated biomarker levels. These results are reported in the manuscript. Biomarker levels disaggregated by sex are reported in the supplementary table 6. Given that sex was found to influence serum p-tau217 levels, sex and age were included as covariates in all multivariable diagnostic models for this biomarker. Fully sex-disaggregated analyses of diagnostic accuracy were not performed, as the study was not designed a priori to assess sex-based differences in biomarker performance. The observed sex effect on p-tau217 levels is acknowledged accordingly in the manuscript.
Reporting on race, ethnicity, or other socially relevant groupings	these variables were not collected and not analysed
Population characteristics	For all ALS, AD cases and controls we collected data on age and sex. In ALS patients from Halle and Milan (n=123), we collected, when available, clinic-demographical data and scores and laboratory results: age at blood sampling, sex, body mass index (BMI), disease duration at blood sampling (time between symptom onset and blood sampling), site of symptom onset26, clinical phenotype, King's stage at blood sampling, ALS Functional Rating Scale-Revised (ALSFRS-R) score and disease progression rate at blood sampling, Edinburgh Cognitive and Behavioural ALS Screen (ECAS) total score at blood sampling, blood levels of creatine kinase (CK), troponin T, myoglobin. Clinical phenotypes comprehended classic, bulbar, upper motor neuron (UMN)-predominant (including also primary lateral sclerosis) and LMN-predominant (including flail arm syndrome, flail leg syndrome and progressive muscular atrophy) ALS. The disease progression rate at blood sampling was calculated as follows: (48–ALSFRS-R score at the time of sampling)/time between symptom onset and blood sampling.
Recruitment	In this retrospective multicentre cohort study, we included ALS and AD (dementia or mild cognitive impairment) cases and non-neurodegenerative disease controls from four different cohorts (total n=315). The case selection was based on the availability of serum samples from subjects with the target conditions and complete information for diagnostic criteria application. Participants were recruited at the Department of Neurology, Martin-Luther-University Halle-Wittenberg, Halle (Saale), Germany (Halle cohort, n=147), Department of Neurology and Laboratory of Neuroscience, IRCCS Istituto Auxologico Italiano, Milan, Italy (Milan cohort, n=90), at the Division for Neurodegenerative Diseases, Neurology Department, Mannheim Center for Translational Medicine, University Medicine Mannheim, Mannheim, Germany (Mannheim cohort 1, n=28), or at the Department of Geriatric Psychiatry, Central Institute of Mental Health, Mannheim, Germany (Mannheim cohort 2, n=50).<br>The Halle cohort included 58 ALS patients, 59 AD patients, and 30 disease controls; the Milan cohort included 65 ALS patients, 17 AD patients, and 8 disease controls; the Mannheim 1 cohort included 16 ALS patients and 12 disease controls; and the Mannheim 2 cohort included 25 AD patients and 25 controls.<br>ALS diagnosis was made according to the Revised El Escorial criteria. The diagnosis of AD was supported by the analysis of CSF core biomarkers, according to the National Institute on Aging and Alzheimer's Association recommendations. Non-neurodegenerative disease controls comprised subjects lacking any clinical, neuroradiological and CSF evidence of neurodegenerative disease.<br>To further investigate the potential impact of age on biomarkers, we included also serum samples from 22 healthy controls aged less than 50 years with normal Mini-Mental State Examination scores, no history of neurological disease and normal serum NfL levels according to our in-house cut-off values. No CSF AD core biomarker assessment was performed in these cases, but given their age, the occurrence of incidental AD co-pathology should be considered unlikely.
Ethics oversight	The study was conducted according to the revised Declaration of Helsinki and Good Clinical Practice guidelines. Informed written consent was obtained from participants and/or their relatives. The study of biosamples and case data was approved by the ethics committees of the Martin-Luther-University Halle-Wittenberg (approval number 2021-101), IRCCS Istituto Auxologico Italiano (approval number 2023_03_21_18), University Medicine Mannheim (approval numbers 2017-589N-MA and 2012-254N-MA) and University of Ulm (approval number 20/10). The study followed the STROBE statement guidelines.

Note that full information on the approval of the study protocol must also be provided in the manuscript.

Field-specific reporting

Please select the one below that is the best fit for your research. If you are not sure, read the appropriate sections before making your selection.

☒ Life sciences ☐ Behavioural & social sciences ☐ Ecological, evolutionary & environmental sciences

For a reference copy of the document with all sections, see nature.com/documents/nr-reporting-summary-flat.pdf

Life sciences study design

All studies must disclose on these points even when the disclosure is negative.

Sample size	No statistical method was used to predetermine sample size. Sample sizes for the biomarker analyses were determined by the availability of serum and CSF samples from participants with the target conditions and with complete clinical information in the four participating cohorts and correspond to a previously published multicentre cohort. Sample sizes for the tissue experiments were determined by the availability of post-mortem brain and spinal cord material and of diagnostic muscle biopsies, which is inherently limited. The resulting group sizes are comparable to or larger than those of previous studies addressing tau isoform distribution in human tissue and were sufficient to detect the between-tissue differences reported here; the tissue findings are explicitly described as preliminary mechanistic evidence.
Data exclusions	No data have been excluded.
Replication	Intra-assay and inter-assay coefficients of variation were below 10% and 15% respectively for all immunoassay measurements. The two-step approach was performed in three independent runs (two manual and one semi-automated) in partly overlapping subgroups, and the biomarker distributions across diagnostic groups were reproduced in all three runs; inter-method reproducibility between the manual and semi-automated protocols was additionally assessed by Spearman correlation and Bland-Altman analysis. All attempts at replication were successful. Absolute concentrations differ between protocols because of the different starting volumes, elution volumes and dilution factors and are therefore reported separately rather than pooled. For the manual BD-Tau-IP, intra-assay CV was 7.2 % and inter-assay CV was 9.5 %. For the semi-automated BD-Tau-IP, intra-assay CV was 6.8 % and inter-assay CV was 8.7 %.
Randomization	Allocation of samples in the ELISA plates was random, also the mass spectrometric analysis of samples was random.
Blinding	All biomarker analyses were measured blind to allocation and diagnose. All mass spectrometric analyses were performed blind to allocation and diagnose.

Reporting for specific materials, systems and methods

We require information from authors about some types of materials, experimental systems and methods used in many studies. Here, indicate whether each material, system or method listed is relevant to your study. If you are not sure if a list item applies to your research, read the appropriate section before selecting a response.

Materials & experimental systems

n/a	Involved in the study	
☐	☒	Antibodies
☐	☐	Eukaryotic cell lines
☐	☐	Palaeontology and archaeology
☐	☐	Animals and other organisms
☐	☒	Clinical data
☐	☐	Dual use research of concern
☐	☐	Plants

Methods

n/a	Involved in the study	
☐	☐	ChIP-seq
☐	☐	Flow cytometry
☐	☐	MRI-based neuroimaging

Antibodies

Antibodies used	The magnetic bead–based immunoprecipitation of BD-tau protein from blood (BD-Tau-IP) used a phosphorylation-insensitive BD-tau antibody mAb 2B8 (847-010200760, Roboscreen GmbH, Germany), which binds to amino acids 121–128 of the 2N4R tau protein at the junction between exon 4 and exon 5, with half of the binding site spanning the C-terminus of exon 4 and the other half the N-terminus of exon 5.<br><br>For the pan-Tau-IP we used functionalized magnetic beads with pan-Tau antibody 7E5 (catalogue number 847-0102006301, Roboscreen GmbH) (Morgado 2025, Morgado 2025).<br><br>For the ptau217-IP we used functionalized magnetic beads with p-tau217 polyclonal antibody (#44-744, Thermo Fisher Scientific, Invitrogen, U.S.A).<br><br>The commercial Simoa assays are stated with their kit item numbers in Methods and supplementary Methods.
Validation	The binding of antibody 2B8 is completely inhibited by a recombinant peptide encompassing the exon 4–5 junction (E4–E5), but not by a peptide spanning the exon 4–4a junction (E4–E4a), indicating a high specificity for BD-tau. See please as reference: oboscreen website: https://www.roboscreen.com/index.php?eID=dumpFile&t=f&f=486&token=d83f35c261e96658ae96017c6485afa75380da51<br><br>Moreover the above mentioned antibodies (mAb 2B8 and 7E5) have been previously validated in a preanalytical Tau-IP, see:<br><br>Morgado, B. et al. Assessment of immunoprecipitation with subsequent immunoassays for the blood-based diagnosis of Alzheimer's disease. Eur. Arch. Psychiatry Clin. Neurosci. 275, 2215–2227 (2025). https://doi.org/10.1007/s00406-023-01751-2

Morgado, B. et al. Evaluation of a fully automated assay for the measurement of plasma pTau217 and a composite score integrating the ratio Aβ1–42/1–40 as biomarkers of Alzheimer's disease. Eur. Arch. Psychiatry Clin. Neurosci. (2025). https://doi.org/10.1007/s00406-025-02123-8

The specificity of the p-tau217 antibody has been confirmed and its successful application in post-mortem AD brain tissue has been demonstratedm see:

Ellis, M. J. et al. Identification of high-performing antibodies for the reliable detection of tau proteoforms by Western blotting and immunohistochemistry. Acta Neuropathol. 147, 87 (2024). https://doi.org/10.1007/s00401-024-02729-7
Kavanagh, T. et al. The interactome of tau phosphorylated at T217 in Alzheimer's disease human brain tissue. Acta Neuropathol. 149, 44 (2025). https://doi.org/10.1007/s00401-025-02881-8

Furthermore, in order to support our findings in blood, we decided to also study BD-tau and big tau peptides in brain, spinal cord and muscle biopsies by mass spectrometry, a highly specific and reliable method.

Eukaryotic cell lines

Policy information about cell lines and Sex and Gender in Research

Cell line source(s): *State the source of each cell line used and the sex of all primary cell lines and cells derived from human participants or vertebrate models.*

Authentication: *Describe the authentication procedures for each cell line used OR declare that none of the cell lines used were authenticated.*

Mycoplasma contamination: *Confirm that all cell lines tested negative for mycoplasma contamination OR describe the results of the testing for mycoplasma contamination OR declare that the cell lines were not tested for mycoplasma contamination.*

Commonly misidentified lines (See ICLAC register): *Name any commonly misidentified cell lines used in the study and provide a rationale for their use.*

Palaeontology and Archaeology

Specimen provenance: *Provide provenance information for specimens and describe permits that were obtained for the work (including the name of the issuing authority, the date of issue, and any identifying information). Permits should encompass collection and, where applicable, export.*

Specimen deposition: *Indicate where the specimens have been deposited to permit free access by other researchers.*

Dating methods: *If new dates are provided, describe how they were obtained (e.g. collection, storage, sample pretreatment and measurement), where they were obtained (i.e. lab name), the calibration program and the protocol for quality assurance OR state that no new dates are provided.*

☐ Tick this box to confirm that the raw and calibrated dates are available in the paper or in Supplementary Information.

Ethics oversight: *Identify the organization(s) that approved or provided guidance on the study protocol, OR state that no ethical approval or guidance was required and explain why not.*

Note that full information on the approval of the study protocol must also be provided in the manuscript.

Animals and other research organisms

Policy information about studies involving animals; ARRIVE guidelines recommended for reporting animal research, and Sex and Gender in Research

Laboratory animals: *For laboratory animals, report species, strain and age OR state that the study did not involve laboratory animals.*

Wild animals: *Provide details on animals observed in or captured in the field; report species and age where possible. Describe how animals were caught and transported and what happened to captive animals after the study (if killed, explain why and describe method; if released, say where and when) OR state that the study did not involve wild animals.*

Reporting on sex: *Indicate if findings apply to only one sex; describe whether sex was considered in study design, methods used for assigning sex. Provide data disaggregated for sex where this information has been collected in the source data as appropriate; provide overall numbers in this Reporting Summary. Please state if this information has not been collected. Report sex-based analyses where performed, justify reasons for lack of sex-based analysis.*

Field-collected samples: *For laboratory work with field-collected samples, describe all relevant parameters such as housing, maintenance, temperature, photoperiod and end-of-experiment protocol OR state that the study did not involve samples collected from the field.*

Ethics oversight: *Identify the organization(s) that approved or provided guidance on the study protocol, OR state that no ethical approval or guidance was required and explain why not.*

Note that full information on the approval of the study protocol must also be provided in the manuscript.

Clinical data

Policy information about clinical studies
All manuscripts should comply with the ICMJE guidelines for publication of clinical research and a completed CONSORT checklist must be included with all submissions.

Clinical trial registration	our study is not a clinical trial
Study protocol	our study is not a clinical trial
Data collection	our study is not a clinical trial
Outcomes	our study is not a clinical trial

Dual use research of concern

Policy information about dual use research of concern

Hazards

Could the accidental, deliberate or reckless misuse of agents or technologies generated in the work, or the application of information presented in the manuscript, pose a threat to:

No	Yes	
☐	☐	Public health
☐	☐	National security
☐	☐	Crops and/or livestock
☐	☐	Ecosystems
☐	☐	Any other significant area

Experiments of concern

Does the work involve any of these experiments of concern:

No	Yes	
☐	☐	Demonstrate how to render a vaccine ineffective
☐	☐	Confer resistance to therapeutically useful antibiotics or antiviral agents
☐	☐	Enhance the virulence of a pathogen or render a nonpathogen virulent
☐	☐	Increase transmissibility of a pathogen
☐	☐	Alter the host range of a pathogen
☐	☐	Enable evasion of diagnostic/detection modalities
☐	☐	Enable the weaponization of a biological agent or toxin
☐	☐	Any other potentially harmful combination of experiments and agents

Plants

Seed stocks	*Report on the source of all seed stocks or other plant material used. If applicable, state the seed stock centre and catalogue number. If plant specimens were collected from the field, describe the collection location, date and sampling procedures.*
Novel plant genotypes	*Describe the methods by which all novel plant genotypes were produced. This includes those generated by transgenic approaches, gene editing, chemical/radiation-based mutagenesis and hybridization. For transgenic lines, describe the transformation method, the number of independent lines analyzed and the generation upon which experiments were performed. For gene-edited lines, describe the editor used, the endogenous sequence targeted for editing, the targeting guide RNA sequence (if applicable) and how the editor was applied.*
Authentication	*Describe any authentication procedures for each seed stock used or novel genotype generated. Describe any experiments used to assess the effect of a mutation and, where applicable, how potential secondary effects (e.g. second site T-DNA insertions, mosiacism, off-target gene editing) were examined.*

ChIP-seq

Data deposition

☐ Confirm that both raw and final processed data have been deposited in a public database such as GEO.

☐ Confirm that you have deposited or provided access to graph files (e.g. BED files) for the called peaks.

Data access links<br>*May remain private before publication.*	*For "Initial submission" or "Revised version" documents, provide reviewer access links. For your "Final submission" document, provide a link to the deposited data.*
Files in database submission	*Provide a list of all files available in the database submission.*
Genome browser session<br>(e.g. UCSC)	*Provide a link to an anonymized genome browser session for "Initial submission" and "Revised version" documents only, to enable peer review. Write "no longer applicable" for "Final submission" documents.*

Methodology

Replicates	*Describe the experimental replicates, specifying number, type and replicate agreement.*
Sequencing depth	*Describe the sequencing depth for each experiment, providing the total number of reads, uniquely mapped reads, length of reads and whether they were paired- or single-end.*
Antibodies	*Describe the antibodies used for the ChIP-seq experiments; as applicable, provide supplier name, catalog number, clone name, and lot number.*
Peak calling parameters	*Specify the command line program and parameters used for read mapping and peak calling, including the ChIP, control and index files used.*
Data quality	*Describe the methods used to ensure data quality in full detail, including how many peaks are at FDR 5% and above 5-fold enrichment.*
Software	*Describe the software used to collect and analyze the ChIP-seq data. For custom code that has been deposited into a community repository, provide accession details.*

Flow Cytometry

Plots

Confirm that:

☐ The axis labels state the marker and fluorochrome used (e.g. CD4-FITC).

☐ The axis scales are clearly visible. Include numbers along axes only for bottom left plot of group (a 'group' is an analysis of identical markers).

☐ All plots are contour plots with outliers or pseudocolor plots.

☐ A numerical value for number of cells or percentage (with statistics) is provided.

Methodology

Sample preparation	*Describe the sample preparation, detailing the biological source of the cells and any tissue processing steps used.*
Instrument	*Identify the instrument used for data collection, specifying make and model number.*
Software	*Describe the software used to collect and analyze the flow cytometry data. For custom code that has been deposited into a community repository, provide accession details.*
Cell population abundance	*Describe the abundance of the relevant cell populations within post-sort fractions, providing details on the purity of the samples and how it was determined.*
Gating strategy	*Describe the gating strategy used for all relevant experiments, specifying the preliminary FSC/SSC gates of the starting cell population, indicating where boundaries between "positive" and "negative" staining cell populations are defined.*

☐ Tick this box to confirm that a figure exemplifying the gating strategy is provided in the Supplementary Information.

Magnetic resonance imaging

Experimental design

Design type	*Indicate task or resting state; event-related or block design.*
Design specifications	*Specify the number of blocks, trials or experimental units per session and/or subject, and specify the length of each trial or block (if trials are blocked) and interval between trials.*

Behavioral performance measures — *State number and/or type of variables recorded (e.g. correct button press, response time) and what statistics were used to establish that the subjects were performing the task as expected (e.g. mean, range, and/or standard deviation across subjects).*

Acquisition

Imaging type(s) — *Specify: functional, structural, diffusion, perfusion.*

Field strength — *Specify in Tesla*

Sequence & imaging parameters — *Specify the pulse sequence type (gradient echo, spin echo, etc.), imaging type (EPI, spiral, etc.), field of view, matrix size, slice thickness, orientation and TE/TR/flip angle.*

Area of acquisition — *State whether a whole brain scan was used OR define the area of acquisition, describing how the region was determined.*

Diffusion MRI ☐ Used ☐ Not used

Preprocessing

Preprocessing software — *Provide detail on software version and revision number and on specific parameters (model/functions, brain extraction, segmentation, smoothing kernel size, etc.).*

Normalization — *If data were normalized/standardized, describe the approach(es): specify linear or non-linear and define image types used for transformation OR indicate that data were not normalized and explain rationale for lack of normalization.*

Normalization template — *Describe the template used for normalization/transformation, specifying subject space or group standardized space (e.g. original Talairach, MNI305, ICBM152) OR indicate that the data were not normalized.*

Noise and artifact removal — *Describe your procedure(s) for artifact and structured noise removal, specifying motion parameters, tissue signals and physiological signals (heart rate, respiration).*

Volume censoring — *Define your software and/or method and criteria for volume censoring, and state the extent of such censoring.*

Statistical modeling & inference

Model type and settings — *Specify type (mass univariate, multivariate, RSA, predictive, etc.) and describe essential details of the model at the first and second levels (e.g. fixed, random or mixed effects; drift or auto-correlation).*

Effect(s) tested — *Define precise effect in terms of the task or stimulus conditions instead of psychological concepts and indicate whether ANOVA or factorial designs were used.*

Specify type of analysis: ☐ Whole brain ☐ ROI-based ☐ Both

Statistic type for inference — *Specify voxel-wise or cluster-wise and report all relevant parameters for cluster-wise methods.*

(See Eklund et al. 2016)

Correction — *Describe the type of correction and how it is obtained for multiple comparisons (e.g. FWE, FDR, permutation or Monte Carlo).*

Models & analysis

n/a	Involved in the study
☐	☐ Functional and/or effective connectivity
☐	☐ Graph analysis
☐	☐ Multivariate modeling or predictive analysis

Functional and/or effective connectivity — *Report the measures of dependence used and the model details (e.g. Pearson correlation, partial correlation, mutual information).*

Graph analysis — *Report the dependent variable and connectivity measure, specifying weighted graph or binarized graph, subject- or group-level, and the global and/or node summaries used (e.g. clustering coefficient, efficiency, etc.).*

Multivariate modeling and predictive analysis — *Specify independent variables, features extraction and dimension reduction, model, training and evaluation metrics.*
